# Metal-free synthesis of 1,4-benzodiazepines and quinazolinones from hexafluoroisopropyl 2-aminobenzoates at room temperature†

**DOI:** 10.1039/d1ra00324k

**Published:** 2021-01-28

**Authors:** Jiewen Chen, En Liang, Jie Shi, Yinrong Wu, Kangmei Wen, Xingang Yao, Xiaodong Tang

**Affiliations:** Guangdong Provincial Key Laboratory of New Drug Screening, School of Pharmaceutical Sciences, Southern Medical University 1023 South Shatai Road, Baiyun District Guangzhou 510515 P. R. China shijie7542@163.com tangxdong@smu.edu.cn

## Abstract

Herein, we describe the novel reactivity of hexafluoroisopropyl 2-aminobenzoates. The metal-free synthesis of 1,4-benzodiazepines and quinazolinones from hexafluoroisopropyl 2-aminobenzoates has been developed at room temperature. These procedures feature good functional group tolerance, mild reaction conditions, and excellent yields. The newly formed products can readily be converted to other useful N-heterocycles. Moreover, the products and their derivatives showed potent anticancer activities *in vitro* by MTT assay.

## Abstract 2

A metal-free synthesis of 1,4-benzodiazepines and quinazolinones from hexafluoroisopropyl 2-aminobenzoates has been developed at room temperature.

## Graphical abstract

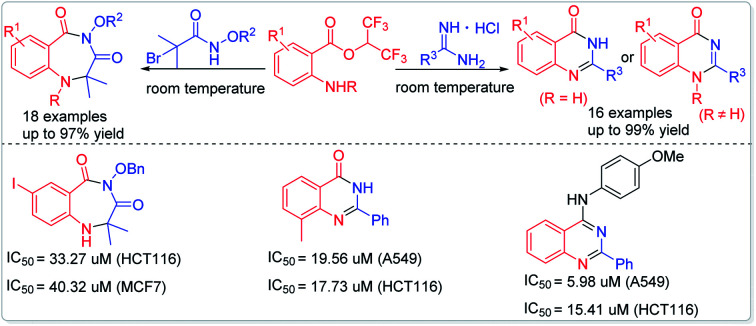

Benzodiazepines (BDZs), especially 1,4-benzodiazepines, are privileged motifs in pharmaceuticals.^1^ For examples, oxazepam is used to treat anxiety disorders or alcohol withdrawal symptoms; triazolam is used to treat insomnia (Scheme 1). Until now, some synthetic approaches to 1,4-benzodiazepine skeletons have been developed include isocyanide-based multicomponent reactions,^2^ cycloadditions,^3^ metal-catalyzed tandem reactions,^4^ and redox-neutral [5+2] annulation with *o*-aminobenzaldehydes.^5^ However, these procedures have some limitations involving unavailable materials, several steps, harsh reaction conditions and low yields. α-Haloamides are widely used to synthesize N-heterocycles.^6^ Recently, some groups reported the synthesis of 1,4-benzodiazepines with α-haloamides.^7^ Kim and coworkers developed a [4+3]-annulation reaction between α-haloamides and isatoic anhydrides for 1,4-benzodiazepines, but the reaction required 80 °C reaction temperature and provided an unsatisfactory yield.^7*a*^ Singh's group reported a two-step method to construct 1,4-benzodiazepines from α-haloamides and anthranils, but the anthranils are not readily available substrates and the second step also required 80 °C reaction temperature.^7*b*^ Quinazolinones are a significant class of heterocycles that widely occur in natural products and pharmaceuticals (Scheme 1).^8^ These compounds exhibit a range of biological activities including anticancer, antibacterial, antiinflammatory, antifungal, *etc.* Due to their significant value, the synthesis of quinazolinones has attracted considerable attention. The reported synthetic methods can be summarized as: (i) condensation of 2-aminobenzamides with carbonyl compounds;^9^ (ii) oxidative cyclization of primary alcohols with 2-aminobenzamides or 2-aminobenzonitriles;^10^ (iii) metal-catalyzed coupling/cyclization reactions;^11^ and (iv) palladium-catalyzed carbonylation reactions.^12^ But these synthetic methods also had some disadvantages. Thus, it is highly desirable to develop new available reagents for synthesizing the useful N-heterocycles such as benzodiazepines and quinazolinones with good yields under mild reaction conditions.

**Scheme 1:**
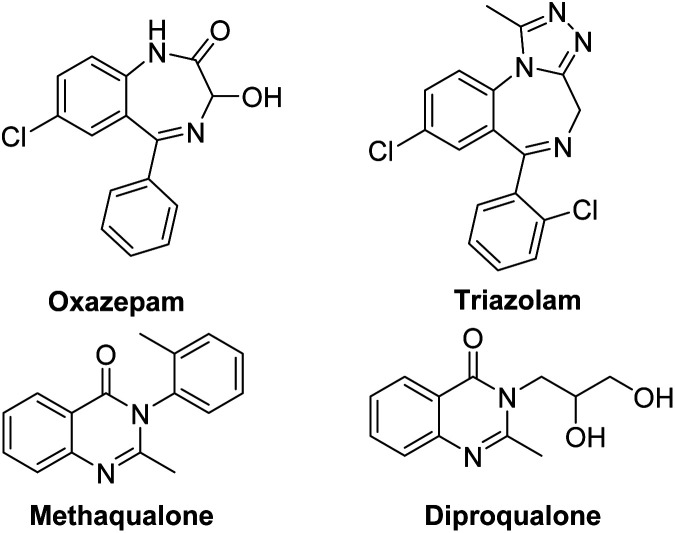
Structures of representative 1,4-benzodiazepine and quinazolinone drugs.

In the past few years, 2-aminobenzoates have been used for the synthesis of N-heterocycles *via* [4+*n*] cyclization (Scheme 2a).^13^ However, harsh reaction conditions such as high reaction temperatures and strong bases or acids were required to effect alkoxy leaving. When we tried to synthesize benzodiazepines or quinazolinones with methyl or *tert*-butyl 2-aminobenzoates, we failed. Recently, hexafluoroisopropanol (HFIP) has attracted a lot of attention when used as solvent or substrate, due to its special properties.^14^ When we used isatoic anhydrides as substrates and NEt_3_ as base in HFIP at room temperature, we unexpectedly discovered that hexafluoroisopropyl 2-aminobenzoates were completely formed. We supposed hexafluoroisopropyl 2-aminobenzoates were good synthons for the synthesis of N-heterocycles. Herein, we report metal-free procedures for the synthesis of 1,4-benzodiazepines and quinazolinones from hexafluoroisopropyl 2-aminobenzoates at room temperature with excellent yields (Scheme 2b).

**Scheme 2:**
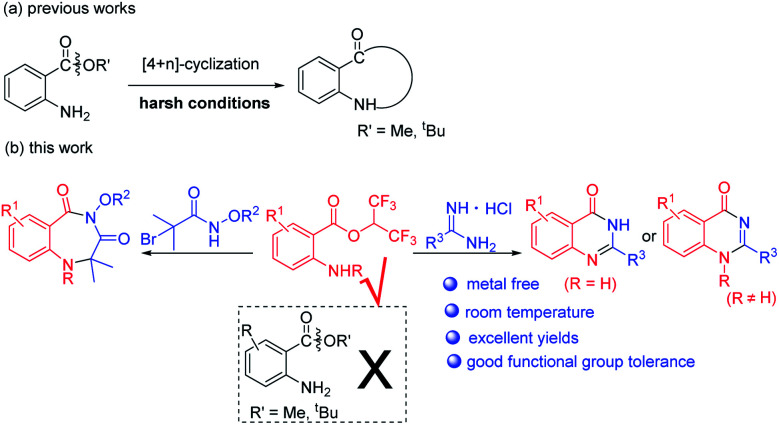
Synthesis of N-heterocycles from 2-aminobenzoates.

We examined the annulation reaction with hexafluoroisopropyl 2-aminobenzoate (1a) and α-bromoamide (2a) as the model substrates. Initially, when the reaction was performed with 1 equiv. of Et_3_N in HFIP at room temperature for 0.5 h, 3a was formed, but cyclization product 4a was not obtained. We thought the transformation from 3a to the product 4a needing to release one molecule of HFIP, and the transformation maybe be inhibited when HFIP was used as solvent. So we removed the solvent HFIP under vacuum and added 2.0 mL DMF to react for 0.5 h. Pleasingly, the desired product 4a was obtained in 68% yield (Table 1, entry 1). Then, a series of bases were checked, and Cs_2_CO_3_ seemed to be the best choice (Table 1, entries 2–7). When using NaHCO_3_ and K_2_CO_3_ as bases, 3a was obtained, but it cannot be converted to the product 4a. The reaction cannot take place without base (Table 1, entry 8). When we replaced the HFIP with another solvent (DMSO, DMA, MeCN, toluene), the reaction cannot occur (Table 1, entries 9–12). The transformation from 3a to product 4a with different solvents was also investigated; the results showed that other solvents, such as DMA, DMSO, MeCN and dioxane, NMP, toluene, were less effective (Table 1, entries 13–16) or ineffective (Table 1, entries 17 and 18). A gram-scale reaction was performed to give product 4a in 92% yield (Table 1, entry 19). We also tried to use a mixture of HFIP and other solvent in order to directly form the desired cyclic compound in a one-pot manner, but the yields was low.

**Table 1:** Optimization of reaction conditions^a^

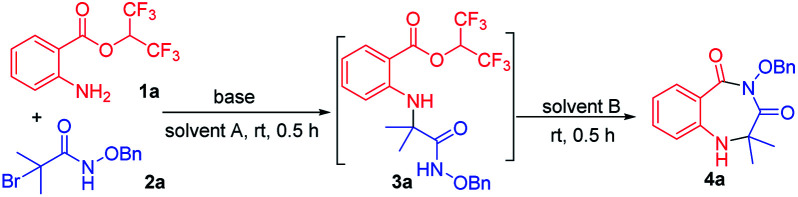				
Entry	Base	Solvent A	Solvent B	Yield (%)
1	Et_3_N	HFIP	DMF	68
2	Cs_2_CO_3_	HFIP	DMF	97
3	NaHCO_3_	HFIP	DMF	n.d.
4	K_2_CO_3_	HFIP	DMF	n.d.
5	DBU	HFIP	DMF	61
6	NaOH	HFIP	DMF	93
7	DIPEA	HFIP	DMF	63
8	—	HFIP	DMF	0
9	Cs_2_CO_3_	DMSO	—	0
10	Cs_2_CO_3_	DMA	—	0
11	Cs_2_CO_3_	MeCN	—	0
12	Cs_2_CO_3_	Toluene	—	0
13	Cs_2_CO_3_	HFIP	DMA	71
14	Cs_2_CO_3_	HFIP	DMSO	41
15	Cs_2_CO_3_	HFIP	MeCN	40
16	Cs_2_CO_3_	HFIP	Dioxane	38
17	Cs_2_CO_3_	HFIP	NMP	n.d.
18	Cs_2_CO_3_	HFIP	Toluene	n.d.
19^b^	Cs_2_CO_3_	HFIP	DMF	92

a Reaction conditions: unless otherwise noted, all reactions were performed with 1a (0.3 mmol), 2a (0.3 mmol), and base (0.3 mmol) in solvent A (3.0 mL) at room temperature for 0.5 h, then the solvent A was removed under vacuum and solvent B (2.0 mL) added to react for 0.5 h. Isolated yield.

b Yield on a 3.0 mmol scale.

After determining the optimized reaction conditions, the scope of the cyclization reaction for 1,4-benzodiazepines was examined (Table 2). Various 5-substituted 2-aminobenzoates bearing halo groups (F, Cl, Br, I) and electron-donating groups (CH_3_, OCH_3_) smoothly underwent cyclization reaction to furnish desired products in good to excellent yields (Table 2, 4b–4g). The nitro group was tolerated in the transformation, but the yield was low (Table 2 and 4h). The reactions also proceeded in the case of 4-substituted 2-aminobenzoates with high yields (Table 2, 4i–4j). When 3-substituted 2-aminobenzoates were employed as substrates, the yields were relatively low because the steric hindrance was unfavorable in intramolecular nucleophilic attack (Table 2, 4k–4l). Furthermore, 4,5-dimethoxy 2-aminobenzoate and 2-(methylamino)benzoate afforded the expected products 4m and 4n in 77% and 64% yields, respectively. In addition, α-bromoamides with diverse *N*-protecting groups (–OCH_3_, –OEt, –O^*t*^Bu, –allyloxy) showed good compatibility, delivering the corresponding products in 61–83% yields (Table 2, 4o–4r). Unfortunately, mono-substituted α-bromohydroxamates, unsubstituted α-bromohydroxamates, and *N*-alkylated bromoacetamides did not react under the current reaction conditions.

**Table 2:** Substrate scope for the synthesis of 1,4-benzodiazepines^a^

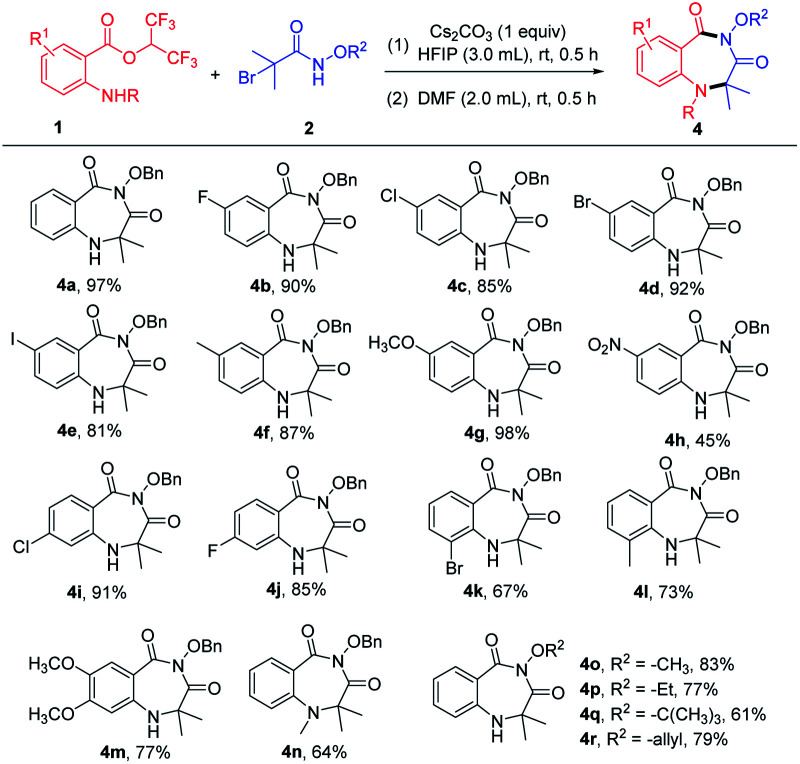

a Reaction conditions: 1 (0.3 mmol), 2 (0.3 mmol), and Cs_2_CO_3_ (0.3 mmol) in solvent HFIP (3.0 mL) at room temperature for 0.5 h, then the HFIP was removed under vacuum and added DMF (2.0 mL) to continue to react for 0.5 h. Isolated yield.

When hexafluoroisopropyl 2-aminobenzoates reacted with amidines hydrochloride in the presence of base, quinazolinones were produced. Then, we optimized the reaction conditions to enhance the yields of the quinazolinones (see the ESI† for more details). With optimum conditions in hand, substrate scope for the synthesis of quinazolinones was next investigated (Table 3). Hexafluoroisopropyl 2-aminobenzoates bearing diverse groups at the amino *para*-position, including F, Cl, Br, I, CH_3_, OCH_3_ and NO_2_ were all compatible with this procedure to afford the cyclization products in excellent yields (Table 3, 6b–6h). In addition, various 4-substituted, 3-substituted and 4,5-disubstituted 2-aminobenzoates reacted well with benzamidine hydrochloride, and the corresponding product yields ranged 86% to 99% (Table 3, 6i–6m). Notably, methyl protected 2-aminobenzoates were also transformed to the product 6n in 99% yield. To our delight, this protocol was also applied to acetamidine hydrochloride and 1,1-dimethylguanidine hydrochloride affording the target products in 97% and 81%, respectively (Table 3, 6o–6p).

**Table 3:** Substrate scope for the synthesis of quinazolinones^a^

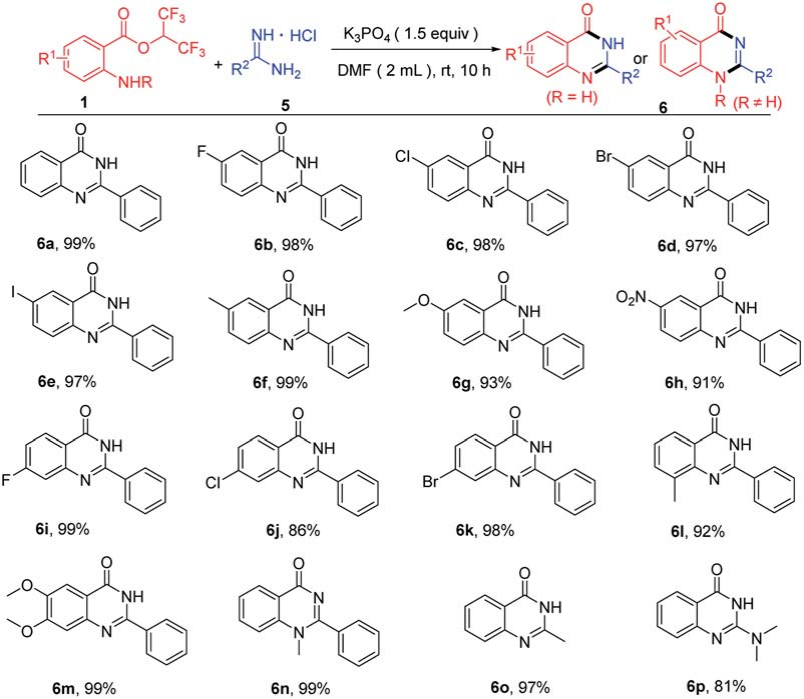

a Reaction conditions: 1 (0.3 mmol), 5 (0.36 mmol), K_3_PO_4_ (0.45 mmol), DMF (2.0 mL) at room temperature for 10 h. Isolated yields.

To probe the reaction mechanism, several preliminary experiments were conducted (Scheme 3). Under standard reaction conditions, methyl 2-aminobenzoates 7 reacted with 2a to provide compound 8 in 92% yield, and 4a was not detected. This control experiment indicated the importance of hexafluoroisopropyl (Scheme 3, eqn (1)). Treatment of methyl 2-aminobenzoates 7 with 5a in the presence of K_3_PO_4_ did not furnish any product 6a, and 3a did not convert at all (Scheme 3, eqn (2)). On the basis of the control experiments and previous reports, we propose a possible mechanism. First, aza-oxyallyl cation A is formed from α-bromoamide with Cs_2_CO_3_.^15^ Whereafter, aza-oxyallyl cation A combines with 1a to produce compound 3a.^15*b*^ The product 4a is obtained *via* intramolecular nucleophilic substitution, releasing a molecule of hexafluoroisopropanol. The nucleophilic attack of 5a onto 1a provides the intermediate B. Subsequently, product 6a is formed by intramolecular nucleophilic addition/deamination cyclization.

**Scheme 3:**
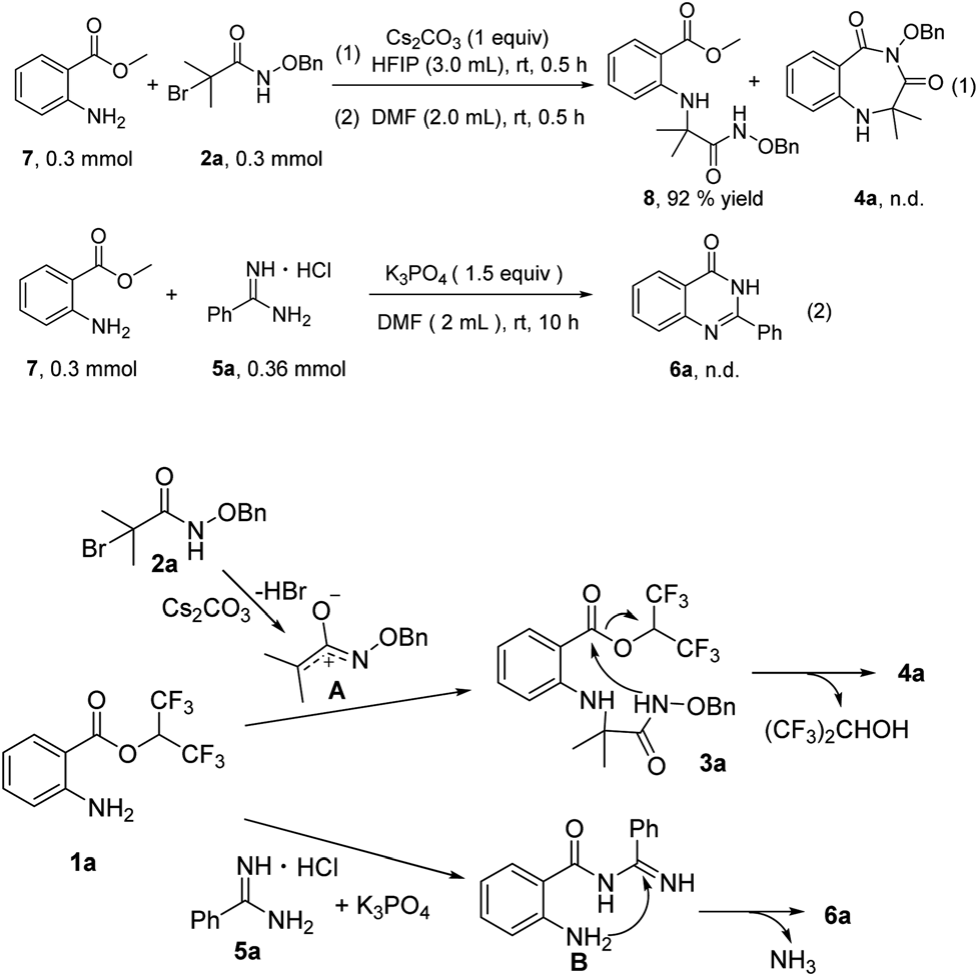
Control experiments and possible reaction mechanism.

In order to address the potential synthetic application of our methods, the transformations of the obtained 1,4-benzodiazepines and quinazolinones were performed (Scheme 4). Compound 9 was formed from 4a through cleavage of the N–O bond with Mo(CO)_6_ (Scheme 4, eqn (1)). The quinazolinones can be transformed into substituted quinazolines with anilines or phenols as nucleophilic reagents in the presence of BOP and DBU (Scheme 4, eqn (2) and (3)).

**Scheme 4:**
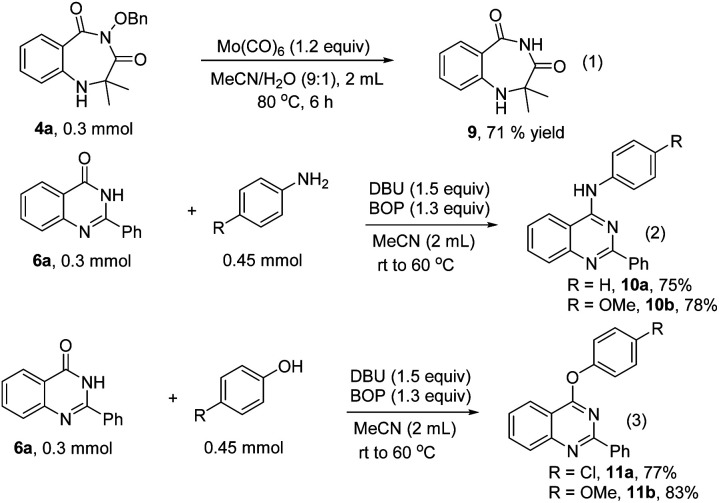
Derivatization of products.

We next investigated the cytotoxicity of the products and their derivatives against cancer cell lines (A549, HCT116 and MCF7) by MTT assay, with 5-fluorouracil (5-FU) as the positive control. To our delight, some products and their derivatives exhibited potent inhibitory activities, and some of them showed better inhibitory activities than 5-Fu (Table 4). These results revealed that our methods had potential applications in discovering new lead compounds with anti-tumor activities.

**Table 4:** Biological applications

Compounds	IC_50_ (μM)		
	A549	HCT116	MCF7
4e	64.69 ± 7.35	33.27 ± 5.84	40.32 ± 0.49
6c	35.11 ± 3.40	26.61 ± 1.26	58.12 ± 3.45
6d	52.32 ± 2.85	23.58 ± 1.50	81.32 ± 2.80
6f	82.89 ± 10.34	59.59 ± 1.60	38.52 ± 1.83
6g	67.00 ± 8.24	32.90 ± 0.60	42.54 ± 3.79
6l	19.56 ± 1.16	17.73 ± 2.32	25.00 ± 5.30
10a	14.79 ± 1.15	26.31 ± 3.95	29.70 ± 0.09
10b	5.98 ± 0.42	15.41 ± 4.41	21.12 ± 1.06
11a	68.54 ± 3.70	17.84 ± 3.13	75.84 ± 2.50
11b	94.76 ± 1.14	25.14 ± 5.31	67.13 ± 3.65
5-Fu	>100	13.03 ± 2.80	29.58 ± 12.86

In summary, we have developed novel and simple approaches for the synthesis of 1,4-benzodiazepines and quinazolinones from hexafluoroisopropyl 2-aminobenzoates with α-bromoamides or amidines hydrochloride. These protocols feature readily available starting materials, mild reaction conditions, good functional group tolerance, and excellent yields. In addition, the newly obtained products and their derivatives showed potent anticancer activities *in vitro* by MTT assay. Further studies on the synthesis of other N-heterocycles from hexafluoroisopropyl 2-aminobenzoates are in progress.

## Conflicts of interest

There are no conflicts to declare.

## Supplementary Material

RA-011-D1RA00324K-s001
