# Supplementary material for: Metal-free synthesis of 1,4-benzodiazepines and quinazolinones from hexafluoroisopropyl 2-aminobenzoates at room temperature
Source: RSC Adv. 2021 Jan 28;11(9):4966–70. doi: 10.1039/d1ra00324k (PMC8694548; doi:10.1039/d1ra00324k)
Supplement: RA-011-D1RA00324K-s001 [file RA-011-D1RA00324K-s001.pdf]

Electronic Supplementary Material (ESI) for RSC Advances.
This journal is © The Royal Society of Chemistry 2021

Supporting Information

Metal-Free Synthesis of 1,4-Benzodiazepines and Quinazolinones from Hexafluoroisopropyl 2-Aminobenzoates at Room Temperature

Jiewen Chen,‡ En Liang,‡ Jie Shi,* Yinrong Wu, Xingang Yao and Xiaodong Tang*

Guangdong Provincial Key Laboratory of New Drug Screening, School of Pharmaceutical Sciences, Southern Medical University, 1023 South Shatai Road, Baiyun District, 510515, China; *E-mail*: shijie7542@163.com; tangxdong@smu.edu.cn

Table of Contents

General Information....................[2]
Experimental Section for Products **4**....................[3]
Experimental Section for Products **6**....................[3]
Cell Culture and Evaluation of the Antiproliferative Activity....................[3]
Characterization Data for All Products....................[3]
References....................[11]
NMR Spectra for All Compounds....................[12]

General Information

^{1}H and ^{13}C NMR spectra were recorded on BRUKER DRX-400 spectrometer with $CDCl_3$ as the solvent. ^{1}H shifts were referenced to $CDCl_3$ at 7.26 ppm. ^{13}C shifts were referenced to $CDCl_3$ at 77 ppm. IR spectra were obtained with an infrared spectrometer on either potassium bromide pellets or liquid films between two potassium bromide pellets. GC-MS data were obtained using electron ionization. HRMS was carried out on a high-resolution mass spectrometer (LCMS-IT-TOF). TLC was performed using commercially available 100−400 mesh silica gel plates (GF_{254}). Unless otherwise stated, all reagents and solvents were purchased from commercial suppliers and used without further purification. α-Bromoamide were prepared according to literature.[1]

General procedure for synthesis of hexafluoroisopropyl 2-aminobenzoates **3**: A round bottom flask equipped with a magnetic stirrer bar was charged with the substituted isatoic anhydrides (3 mmol) in HFIP (6 mL). NEt_3 (3 mmol) was added and the reaction was stirred at room temperature for 1 h. The mixture was quenched with aqueous solution of $NaHCO_3$ (20 mL) and extracted with EtOAc (3 × 20 mL). The combined organic layers were dried over anhydrous $MgSO_4$ and the solvent was removed under vacuum. The products can be used without further purification.

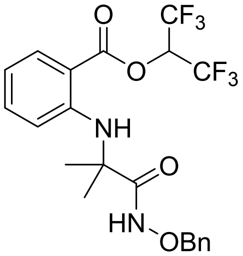

1,1,1,3,3,3-Hexafluoropropan-2-yl 2-((1-((benzyloxy)amino)-2-methyl-1-oxopropan-2-yl)amino)benzoate (3a)[1]

^{1}H NMR (400 MHz, $CDCl_3$) δ 8.96 (s, 1H), 8.02 (dd, J = 8.1, 1.6 Hz, 1H), 7.64 (s, 1H), 7.45 – 7.35 (m, 1H), 7.32 – 7.28 (m, 5H), 6.78 (ddd, J = 8.1, 7.1, 1.0 Hz, 1H), 6.62 (d, J = 8.6 Hz, 1H), 6.01 – 5.90 (m, 1H), 4.86 (s, 2H), 1.58 (s, 6H). ^{13}C NMR (100 MHz, $CDCl_3$) δ 172.2, 164.6, 149.2, 136.2, 134.8, 132.2, 129.3, 128.8, 128.6, 128.5, 117.2, 115.0, 108.6, 78.1, 67.0 – 65.6 (m), 56.9, 26.1.

Table 1. Optimization of the reaction conditions [a]

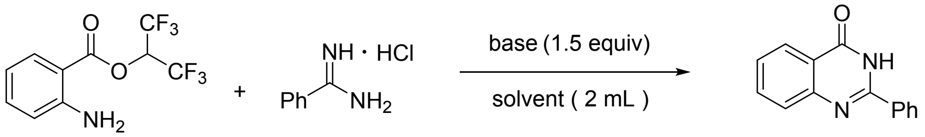

1a **5a** **6a**

Entry	Base	Solvent	Yield [%]
1	NEt_3	DMF	53
2	Na_2CO_3	DMF	92
3	K_2CO_3	DMF	96
4	Cs_2CO_3	DMF	97
5	K_3PO_4	DMF	99
6	DBU	DMF	95
7	$NaHCO_3$	DMF	89
8	-	DMF	0
9	K_3PO_4	MeCN	98
10	K_3PO_4	toluene	81
11	K_3PO_4	dioxane	90
12	K_3PO_4	THF	97
13	K_3PO_4	CH_2Cl_2	68

14	K_3PO_4	DMSO	94
15[b]	K_3PO_4	DMF	83

[a] Reaction conditions: unless otherwise noted, all reactions were performed with **1a** (0.3 mmol), **5a** (0.36 mmol), base (0.45 mmol) in solvent (2.0 mL) at room temperature for 10 h. Isolated yield.
[b] 1 equiv K_3PO_4 was used.

Experimental Section for Products 4

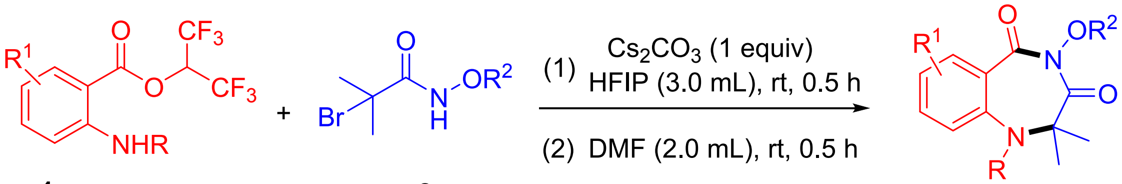

General procedure for products **4**: 2-aminobenzoates **1** (0.3 mmol), α-bromoamide **2** (0.3 mmol), Cs_2CO_3 (0.3 mmol), HFIP (3.0 mL), were added to a 25 mL tube with magnetic stirrer bar. The reaction mixture was stirred at room temperature for 0.5 h, the HFIP was removed under vacuum and added DMF (2.0 mL) to continue to react for 0.5 h. The mixture was quenched with aqueous solution of $NaHCO_3$ (10 mL) and extracted with EtOAc (3 × 10 mL). The combined organic layers were dried over anhydrous $MgSO_4$ and the solvent was removed under vacuum. The crude product was purified by column chromatography (EtOAc/petroleum ether) on silica gel.

Experimental Section for Products 6

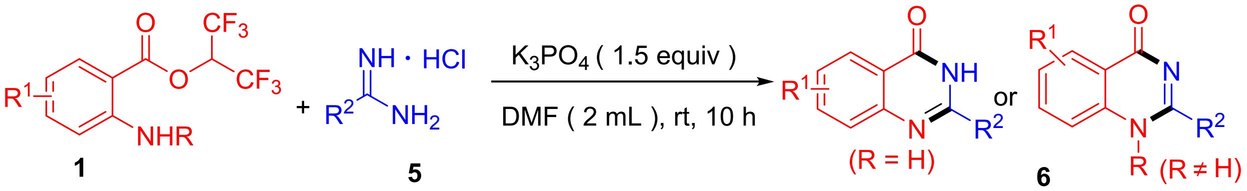

General procedure for products **6**: 2-aminobenzoates **1** (0.3 mmol), amidines hydrochloride **5** (0.36 mmol), K_3PO_4 (0.45 mmol), DMF (2.0 mL), were added to a 25 mL tube with magnetic stirrer bar. The reaction mixture was stirred at room temperature for 10 h. The mixture was quenched with aqueous solution of $NaHCO_3$ (10 mL) and extracted with EtOAc (3 × 10 mL). The combined organic layers were dried over anhydrous $MgSO_4$ and the solvent was removed under vacuum. The crude product was purified by column chromatography (EtOAc/petroleum ether) on silica gel.

Cell Culture and Evaluation of the Antiproliferative Activity

The compounds were evaluated for their in vitro cytotoxicity against the human cancer cell lines HCT116, A549 and MCF7 by 3-(4,5-dimethylthiazol-2-yl)-2,5 -diphenyltetrazolium Bromide (MTT) assay. The cancer cell lines were purchased from American Type Culture Collection (ATCC). A549、 HCT116、MCF7 cells were maintained in RPMI-1640 medium (Gibco). The medium for all cell lines were supplemented with 10% fetal bovine serum (FBS, Invitrogen, Carlsbad, CA) and 1% penicillin-streptomycin (Life Technologies, USA) and maintained in a humidified incubator at 37 °C adjusted to 5% CO_2. Cells were seeded into 96-well plates at a density of 5000 cells/well. On the next day, medium containing the new compounds at different concentrations was added into per well for at least three cell doublings and incubated at 37°C for another 48 h, with 5-Fluorouracil (FU) as the positive control. At the indicated time, the culture medium was replaced with 100 μL medium containing 10% MTT solution (5 mg/mL in PBS) and further incubated for 4 h. The absorbance was detected with a microplate reader at a wavelength of 570 nm. The IC_{50} values were calculated by nonlinear regression analysis using GraphPad Prism 8.0.

Characterization Data for All Products

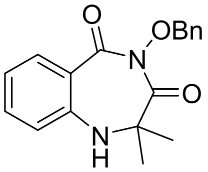

4-(Benzyloxy)-2,2-dimethyl-1,2-dihydro-3*H*-benzo[*e*][1,4]diazepine-3,5(4*H*)-dione (4a)[1]
Yellow solid (90.3 mg, 97%); m.p.=120-122 °C. ^{1}H NMR (400 MHz, $CDCl_3$) δ 8.23 (dd, J = 8.2, 1.4 Hz, 1H), 7.54 (dd, J = 7.3, 2.0 Hz, 2H), 7.41 – 7.30 (m, 4H), 6.99 (t, J = 7.6 Hz, 1H), 6.76 (d, J = 7.7 Hz, 1H), 5.03 (s, 2H), 1.43 (s, 6H).

^{13}C NMR (100 MHz, $CDCl_3$) δ 171.7, 163.5, 144.7, 134.4, 134.4, 133.2, 129.8, 128.7, 128.3, 120.6, 119.8, 117.9, 77.7, 57.5, 24.6. IR (KBr): 2384, 2345, 1726, 1663, 1608, 1494, 1136, 772 cm^{-1}.

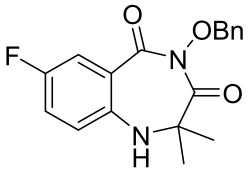

4-(Benzyloxy)-7-fluoro-2,2-dimethyl-1,2-dihydro-3*H*-benzo[*e*][1,4]diazepine-3,5(4*H*)-dione (4b)[2]
Light yellow solid (88.7 mg, 90%); m.p.=96-97 °C. ^1H NMR (400 MHz, $CDCl_3$) δ 7.88 (dd, J = 9.8, 2.9 Hz, 1H), 7.55 – 7.49 (m, 2H), 7.34 – 7.29 (m, 3H), 7.14 – 7.06 (m, 1H), 6.76 (dd, J = 8.8, 4.5 Hz, 1H), 5.01 (s, 2H), 1.39 (s, 6H). ^{13}C NMR (100 MHz, $CDCl_3$) δ 171.8, 162.5 (d, J = 2 Hz), 156.9 (d, J = 239 Hz), 141.2, 134.2, 129.8, 128.8, 128.3, 122.4 (d, J = 24 Hz), 121.6 (d, J = 7 Hz), 118.1, 117.8, 77.7, 57.8, 24.6. IR (KBr): 3357, 2379, 1722, 1663, 1498. 1285, 1213, 1154 cm^{-1}.

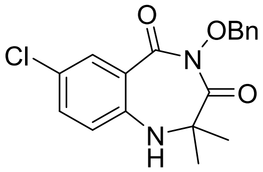

4-(Benzyloxy)-7-chloro-2,2-dimethyl-1,2-dihydro-3*H*-benzo[*e*][1,4]diazepine-3,5(4*H*)-dione (4c)[2]
Brown solid (87.9 mg, 85%); m.p.=141-144 °C. ^1H NMR (400 MHz, $CDCl_3$) δ 8.17 (d, J = 2.5 Hz, 1H), 7.51 (dd, J = 6.6, 2.9 Hz, 2H), 7.34 – 7.30 (m, 3H), 7.28 – 7.24 (m, 1H), 6.73 (d, J = 8.6 Hz, 1H), 5.01 (s, 2H), 1.41 (s, 6H). ^{13}C NMR (100 MHz, $CDCl_3$) δ 171.2, 162.5, 143.3, 134.4, 134.2, 132.1, 129.8, 128.8, 128.3, 125.6, 121.3, 118.5, 77.8, 57.4, 24.4. IR (KBr): 3358, 1720, 1662, 1485, 1392, 1286, 1216, 1150, 699 cm^{-1}.

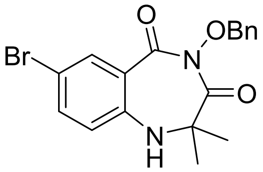

4-(Benzyloxy)-7-bromo-2,2-dimethyl-1,2-dihydro-3*H*-benzo[*e*][1,4]diazepine-3,5(4*H*)-dione (4d)[2]
Light yellow solid (107.4 mg, 92%); m.p.=136-137 °C. ^1H NMR (400 MHz, $CDCl_3$) δ 8.32 (d, J = 2.4 Hz, 1H), 7.52 – 7.49 (m, 2H), 7.40 (dd, J = 8.6, 2.5 Hz, 1H), 7.35 – 7.30 (m, 3H), 6.68 (d, J = 8.6 Hz, 1H), 5.00 (s, 2H), 1.41 (s, 6H). ^{13}C NMR (100 MHz, $CDCl_3$) δ 171.1, 162.5, 143.7, 137.1, 135.1, 134.2, 129.8, 128.9, 128.3, 121.6, 118.8, 112.4, 77.8, 57.3, 24.4. IR (KBr): 2377, 1718, 1662, 1479, 1390, 1285, 1149, 698 cm^{-1}.

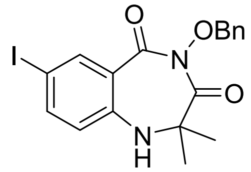

4-(Benzyloxy)-7-iodo-2,2-dimethyl-1,2-dihydro-3*H*-benzo[*e*][1,4]diazepine-3,5(4*H*)-dione (4e)
Light yellow solid (106.0 mg, 81%); m.p.=126-128 °C. ^1H NMR (400 MHz, $CDCl_3$) δ 8.49 (d, J = 2.0 Hz, 1H), 7.56 (dd, J = 8.5, 2.1 Hz, 1H), 7.52 – 7.49 (m, 2H), 7.34 – 7.30 (m, 3H), 6.55 (d, J = 8.5 Hz, 1H), 5.00 (s, 2H), 1.41 (s, 6H). ^{13}C NMR (100 MHz, $CDCl_3$) δ 171.0, 162.4, 144.2, 142.6, 141.1, 134.1, 129.8, 128.8, 128.3, 121.7, 119.2, 81.5, 77.8,57.1,24.4. IR (KBr): 3366, 2386, 1718,1661, 1473, 1387, 1285, 697 cm^{-1}. HRMS (ESI) m/z: $[M+H]^+$ Calcd for $C_{18}H_{18}IN_2O_3$ 437.0357; Found 437.0351.

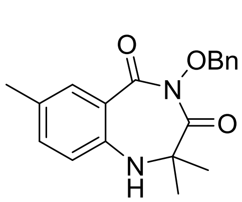

4-(Benzyloxy)-2,2,7-trimethyl-1,2-dihydro-3*H*-benzo[*e*][1,4]diazepine-3,5(4*H*)-dione (4f)[2]

Yellow solid (84.7 mg, 87%); m.p.=101-103 °C. ^{1}H NMR (400 MHz, $CDCl_3$) δ 8.01 (d, J = 2.1 Hz, 1H), 7.54 (dd, J = 7.3, 2.3 Hz, 2H), 7.34 – 7.30 (m, 3H), 7.17 (dd, J = 8.2, 2.1 Hz, 1H), 6.69 (d, J = 8.1 Hz, 1H), 5.01 (s, 2H), 2.30 (s, 3H), 1.39 (s, 6H). ^{13}C NMR (100 MHz, $CDCl_3$) δ 172.0, 163.5, 142.7, 135.6, 134.4, 132.5, 130.0, 129.7, 128.6, 128.2, 120.0, 117.7, 77.6, 57.6, 24.6, 20.3. IR (KBr): 3356, 1718, 1663, 1505, 1293, 1218, 1150, 699 cm^{-1}.

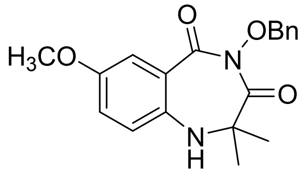

4-(Benzyloxy)-7-methoxy-2,2-dimethyl-1,2-dihydro-3*H*-benzo[*e*][1,4]diazepine-3,5(4*H*)-dione (4g)
Brown solid (100.0 mg, 98%); m.p.=108-110 °C. ^{1}H NMR (400 MHz, $CDCl_3$) δ 7.66 (d, J = 3.0 Hz, 1H), 7.54 (dd, J = 7.3, 2.3 Hz, 2H), 7.35 – 7.30 (m, 3H), 6.99 (dd, J = 8.8, 3.0 Hz, 1H), 6.72 (d, J = 8.8 Hz, 1H), 5.02 (s, 2H), 3.80 (s, 3H), 1.38 (s, 6H). ^{13}C NMR (100 MHz, $CDCl_3$) δ 172.4, 163.2, 153.8, 139.1, 134.4, 129.7, 128.7, 128.2, 123.7, 121.7, 118.7, 113.8, 77.6, 58.0, 55.6, 24.4. IR (KBr): 3363, 1715, 1658, 1500, 1290, 1221, 1143, 747 cm^{-1}. HRMS (ESI) m/z: $[M+H]^+$ Calcd for $C_{19}H_{21}N_2O_4$ 341.1496; Found 341.1487.

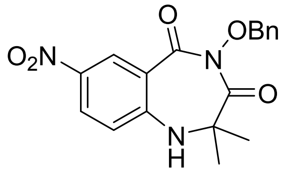

4-(Benzyloxy)-2,2-dimethyl-7-nitro-1,2-dihydro-3*H*-benzo[*e*][1,4]diazepine-3,5(4*H*)-dione (4h)[2]
Yellow solid (48.0 mg, 45%); m.p.=178-179 °C. ^{1}H NMR (400 MHz, $CDCl_3$) δ 9.13 (d, J = 2.5 Hz, 1H), 8.18 (dd, J = 9.0, 2.5 Hz, 1H), 7.49 (dd, J = 6.4, 2.5 Hz, 2H), 7.33 – 7.29 (m, 3H), 6.83 (d, J = 9.0 Hz, 1H), 5.04 (s, 2H), 1.52 (s, 6H). ^{13}C NMR (100 MHz, $CDCl_3$) δ 169.3, 162.2, 148.6, 140.6, 133.9, 130.6, 130.0, 129.1, 128.8, 128.4, 119.7, 115.6, 78.1, 57.0, 24.3. IR (KBr): 2376, 2331, 1718, 1617, 1509, 1333, 698 cm^{-1}.

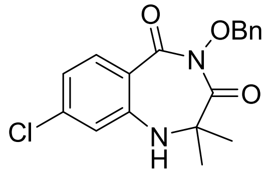

4-(Benzyloxy)-8-chloro-2,2-dimethyl-1,2-dihydro-3*H*-benzo[*e*][1,4]diazepine-3,5(4*H*)-dione (4i)[1]
Light yellow solid (94.1 mg, 91%); m.p.=146-147 °C. ^{1}H NMR (400 MHz, $CDCl_3$) δ 8.15 (d, J = 8.8 Hz, 1H), 7.51 (dd, J = 6.4, 2.7 Hz, 2H), 7.33 – 7.31 (m, 3H), 6.93 (dd, J = 8.8, 1.8 Hz, 1H), 6.81 (d, J = 1.8 Hz, 1H), 5.01 (s, 2H), 1.41 (s, 6H). ^{13}C NMR (100 MHz, $CDCl_3$) δ 171.1, 162.8, 145.5, 140.5, 134.7, 134.2, 129.8, 128.8, 128.3, 120.9, 119.1, 116.0, 77.7, 57.3, 24.5. IR (KBr): 3358, 1719, 1663, 1598, 1273, 1218, 1136, 697 cm^{-1}.

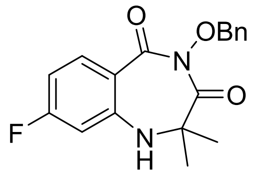

4-(Benzyloxy)-8-fluoro-2,2-dimethyl-1,2-dihydro-3*H*-benzo[*e*][1,4]diazepine-3,5(4*H*)-dione (4j)[2]
White solid (83.7 mg, 85%); m.p.=129-130 °C. ^{1}H NMR (400 MHz, $CDCl_3$) δ 8.24 (dd, J = 9.1, 6.5 Hz, 1H), 7.55 – 7.48 (m, 2H), 7.33– 7.31 (m, 3H), 6.74 – 6.64 (m, 1H), 6.47 (dd, J = 9.8, 2.4 Hz, 1H), 5.01 (s, 2H), 1.42 (s, 6H). ^{13}C NMR (100 MHz, $CDCl_3$) δ 171.0, 166.4 (d, J = 254 Hz), 162.7, 146.8 (d, J = 12 Hz), 136.3 (d, J = 11 Hz), 134.3, 129.8, 128.8, 128.3, 114.2, 108.8 (d, J = 22 Hz), 105.4 (d, J = 24 Hz), 77.7, 57.3, 24.5. IR (KBr): 3359, 1718, 1663, 1614, 1279, 1229, 746,698 cm^{-1}.

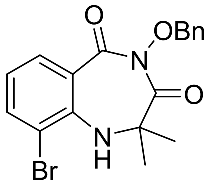

4-(Benzyloxy)-9-bromo-2,2-dimethyl-1,2-dihydro-3*H*-benzo[*e*][1,4]diazepine-3,5(4*H*)-dione (4k)

Yellow oil (78.2 mg, 67%). ^{1}H NMR (400 MHz, $CDCl_3$) δ 8.20 (dd, *J* = 8.2, 1.5 Hz, 1H), 7.70 (dd, *J* = 7.8, 1.5 Hz, 1H), 7.53 (dd, *J* = 7.3, 2.3 Hz, 2H), 7.37 – 7.31 (m, 3H), 6.88 (t, *J* = 7.9 Hz, 1H), 5.03 (s, 2H), 1.46 (s, 6H). ^{13}C NMR (100 MHz, $CDCl_3$) δ 171.7, 162.8, 141.9, 137.4, 134.3, 133.0, 129.8, 128.8, 128.3, 121.0, 119.7, 114.5, 77.8, 58.0, 24.7. IR (KBr): 3394, 1723, 1667, 1502, 1454, 1284, 743 cm^{-1}. HRMS (ESI) m/z: $[M+H]^+$ Calcd for $C_{18}H_{18}BrN_2O_3$ 389.0495; Found 389.0487.

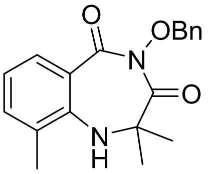

4-(Benzyloxy)-2,2,9-trimethyl-1,2-dihydro-3*H*-benzo[*e*][1,4]diazepine-3,5(4*H*)-dione (4l)[2]

White solid (71.0 mg, 73%); m.p.=82-84 °C. ^{1}H NMR (400 MHz, $CDCl_3$) δ 8.12 (d, *J* = 7.8 Hz, 1H), 7.58 – 7.52 (m, 2H), 7.37 – 7.29 (m, 4H), 6.91 (t, *J* = 7.7 Hz, 1H), 5.03 (s, 2H), 2.23 (s, 3H), 1.44 (s, 6H). ^{13}C NMR (100 MHz, $CDCl_3$) δ 172.0, 163.8, 143.0, 135.4, 134.5, 131.4, 129.7, 128.7, 128.2, 125.7, 120.0, 118.4, 77.6, 57.4, 24.9, 17.5. IR (KBr): 3424, 1717, 1659, 1597, 1468, 1284, 1139, 747 cm^{-1}.

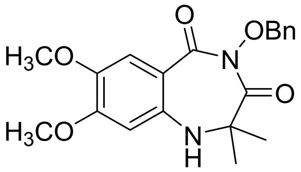

4-(Benzyloxy)-7,8-dimethoxy-2,2-dimethyl-1,2-dihydro-3*H*-benzo[*e*][1,4]diazepine-3,5(4*H*)-dione (4m)[3]

Brown solid (86.6 mg, 77%); m.p.=165-166 °C. ^{1}H NMR (400 MHz, $CDCl_3$) δ 7.61 (s, 1H), 7.53 (dd, *J* = 7.2, 1.9 Hz, 2H), 7.34 – 7.30 (m, 3H), 6.20 (s, 1H), 5.01 (s, 2H), 3.87 (s, 3H), 3.84 (s, 3H), 1.40 (s, 6H). ^{13}C NMR (100 MHz, $CDCl_3$) δ 171.6, 162.8, 154.9, 143.9, 140.9, 134.5, 129.7, 128.7, 128.3, 113.2, 109.6, 101.8, 77.6, 57.4, 56.1, 56.0, 24.4. IR (KBr): 1710, 1650, 1613, 1506, 1383, 1239, 776, 698 cm^{-1}.

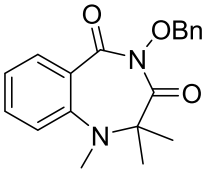

4-(Benzyloxy)-1,2,2-trimethyl-1,2-dihydro-3*H*-benzo[*e*][1,4]diazepine-3,5(4*H*)-dione (4n)

Yellow oil (45.0 mg, 64%). ^{1}H NMR (400 MHz, $CDCl_3$) δ 8.15 (d, *J* = 8.0 Hz, 1H), 7.56 – 7.45 (m, 3H), 7.38 – 7.31 (m, 3H), 7.19 – 7.09 (m, 2H), 5.08 (s, 2H), 2.88 (s, 3H), 1.34 (s, 6H). ^{13}C NMR (100 MHz, $CDCl_3$) δ 175.0, 163.9, 148.9, 134.6, 134.0, 132.2, 129.9, 128.8, 128.3, 125.8, 124.6, 123.3, 77.6, 62.9, 37.7, 24.6. IR (KBr): 2366, 2334, 1663,1224, 1135, 753, 699 cm^{-1}. HRMS (ESI) m/z: $[M+H]^+$ Calcd for $C_{19}H_{21}N_2O_3$ 325.1547; Found 325.1541.

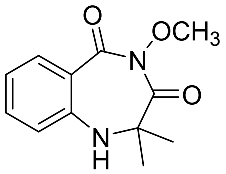

4-Methoxy-2,2-dimethyl-1,2-dihydro-3*H*-benzo[*e*][1,4]diazepine-3,5(4*H*)-dione (4o)[2]

Yellow oil (58.3 mg, 83%). ^{1}H NMR (400 MHz, $CDCl_3$) δ 8.25 (d, *J* = 7.6 Hz, 1H), 7.38 (t, *J* = 8.0 Hz, 1H), 6.99 (t, *J* = 8.0 Hz, 1H), 6.76 (d, *J* = 8.0 Hz, 1H), 3.88 (s, 3H), 1.46 (s, 6H).^{13}C NMR (100 MHz, $CDCl_3$) δ 171.4, 163.2, 144.8, 134.5, 133.2, 120.5, 119.8, 117.7, 63.5, 57.3, 24.6. IR (KBr): 3351, 1719, 1661, 1606, 1294, 1221, 902, 753 cm^{-1}.

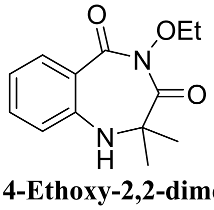

4-Ethoxy-2,2-dimethyl-1,2-dihydro-3*H*-benzo[*e*][1,4]diazepine-3,5(4*H*)-dione (4p)[2]

Yellow oil (57.4 mg, 77%). ^{1}H NMR (400 MHz, $CDCl_3$) δ 8.21 (d, J = 7.4 Hz, 1H), 7.38 – 7.33 (m, 1H), 6.98 – 6.94 (m, 1H), 6.79 (d, J = 8.4 Hz, 1H), 4.04 (q, J = 7.1 Hz, 2H), 1.43 (s, 6H), 1.30 (t, J = 7.1 Hz, 3H). ^{13}C NMR (100 MHz, $CDCl_3$) δ 171.8, 163.6, 144.8, 134.4, 133.1, 120.4, 119.7, 117.6, 71.4, 57.3, 24.6, 13.3. IR (KBr): 3350, 1718, 1661, 1293, 1221, 1136, 925, 752 cm^{-1}.

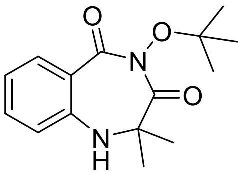

4-(*tert*-Butoxy)-2,2-dimethyl-1,2-dihydro-3*H*-benzo[*e*][1,4]diazepine-3,5(4*H*)-dione (4q)[2]

White solid (50.6 mg, 61%). m.p.=196-197 °C. ^{1}H NMR (400 MHz, $CDCl_3$) δ 8.18 (d, J = 8.0 Hz, 1H), 7.40 – 7.36 (m, 1H), 6.99 – 6.95 (m, 1H), 6.81 (d, J = 8.4 Hz, 1H), 1.68 (s, 3H), 1.23 (s, 9H), 1.19 (s, 3H). ^{13}C NMR (100 MHz, $CDCl_3$) δ 175.6, 166.2, 145.2, 134.3, 133.1, 120.3, 119.6, 118.0, 84.4, 58.2, 28.7, 26.6, 21.7. IR (KBr): 3311, 1727, 1654, 1606, 1293, 1103, 920, 755 cm^{-1}.

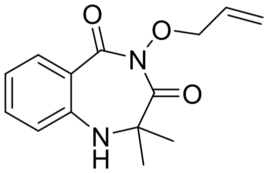

4-(Allyloxy)-2,2-dimethyl-1,2-dihydro-3*H*-benzo[*e*][1,4]diazepine-3,5(4*H*)-dione (4r)[2]

Brown oil (61.7 mg, 79%). ^{1}H NMR (400 MHz, $CDCl_3$) δ 8.22 (d, J = 8.0 Hz, 1H), 7.39 – 7.35 (m, 1H), 7.00 – 6.96 (m, 1H), 6.77 (d, J = 8.0 Hz, 1H), 6.10 – 6.00 (m, 1H), 5.38 – 5.24 (m, 2H), 4.51 (d, J = 6.6 Hz, 2H), 1.45 (s, 6H). ^{13}C NMR (100 MHz, $CDCl_3$) δ 171.8, 163.6, 144.7, 134.4, 133.2, 131.6, 121.1, 120.5, 119.8, 117.8, 76.9, 57.5, 24.8. IR (KBr): 3379, 1715, 1655, 1606, 1293, 1135, 752 cm^{-1}.

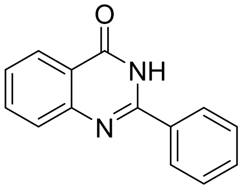

2-Phenylquinazolin-4(3*H*)-one (6a)[4]

White solid (66.0 mg, 99%); m.p.=244-245 °C. ^{1}H NMR (400 MHz, $CDCl_3$) δ 11.83 (br, 1H), 8.33 (d, J = 7.8 Hz, 1H), 8.31 – 8.24 (m, 2H), 7.88 – 7.76 (m, 2H), 7.62 – 7.55 (m, 3H), 7.51 (t, J = 7.4 Hz, 1H). ^{13}C NMR (100 MHz, $CDCl_3$) δ 164.0, 151.9, 149.4, 134.9, 132.7, 131.7, 129.0, 127.9, 127.5, 126.8, 126.3, 120.8. IR (KBr): 2364, 2331, 1709, 1666, 1602, 1342, 1293, 767 cm^{-1}.

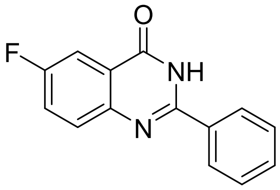

6-Fluoro-2-phenylquinazolin-4(3*H*)-one (6b)[5]

White solid (70.6 mg, 98%); m.p.=240-241 °C. ^{1}H NMR (400 MHz, DMSO-d_6) δ 12.55 (br, 1H), 8.17 (d, J = 7.1 Hz, 2H), 7.85 – 7.79 (m, 2H), 7.73 (t, J = 8.7 Hz, 1H), 7.63 – 7.51 (m, 3H). ^{13}C NMR (100 MHz, DMSO-d_6) δ 161.8, 160.0 (d, J = 244 Hz), 152.0, 145.6, 132.6, 131.5, 130.3, 128.7, 127.8, 123.1(d, J = 24 Hz), 122.3(d, J = 8 Hz), 110.6 (d, J = 23 Hz). IR (KBr): 2362, 2335, 1687, 1610, 1567, 1522, 687 cm^{-1}.

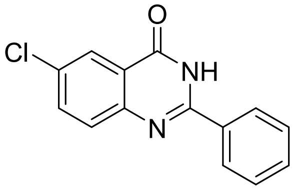

6-Chloro-2-phenylquinazolin-4(3*H*)-one (6c)[4]

White solid (75.5 mg, 98%); m.p.=292-293 °C. ^{1}H NMR (400 MHz, DMSO-d_6) δ 12.66 (br, 1H), 8.17 (d, *J* = 7.5 Hz, 2H), 8.08 (s, 1H), 7.86 (d, *J* = 8.6 Hz, 1H), 7.76 (d, *J* = 8.7 Hz, 1H), 7.60 – 7.53 (m, 3H). ^{13}C NMR (100 MHz, DMSO-d_6) δ 161.4, 152.9, 147.6, 134.8, 132.5, 131.7, 130.9, 129.8, 128.7, 127.9, 125.0, 122.3. IR (KBr): 2364, 2329, 1296, 1673, 1531, 1197, 770, 697 cm^{-1}.

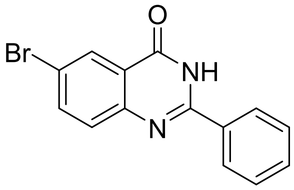

6-Bromo-2-phenylquinazolin-4(3*H*)-one (6d)[4]

White solid (88.5 mg, 97%); m.p.=256-257 °C. ^{1}H NMR (400 MHz, DMSO-d_6) δ 12.66 (br, 1H), 8.22 (d, *J* = 2.4 Hz, 1H), 8.19 – 8.15 (m, 2H), 7.97 (dd, *J* = 8.7, 2.4 Hz, 1H), 7.69 (d, *J* = 8.7 Hz, 1H), 7.62 –7.53 (m, 3H). ^{13}C NMR (100 MHz, DMSO-d_6) δ 161.3, 153.1, 147.9, 137.6, 132.6, 131.8, 130.0, 128.8, 128.1, 128.0, 122.7, 119.1. IR (KBr): 2366, 2329, 1853, 1673, 1610, 1471, 1299, 681 cm^{-1}.

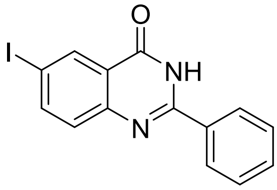

6-Iodo-2-phenylquinazolin-4(3*H*)-one (6e)[6]

White solid (101.3 mg, 97%); m.p.= 236-237 °C. ^{1}H NMR (400 MHz, DMSO-d_6) δ 12.62 (br, 1H), 8.42 (s, 1H), 8.17 (d, *J* = 7.4 Hz, 2H), 8.10 (d, *J* = 1.8 Hz, 1H), 7.64 – 7.49 (m, 4H). ^{13}C NMR (100 MHz, DMSO-d_6) δ 155.7, 143.0, 143.0, 134.3, 132.6, 131.7, 129.7, 128.7, 127.9, 126.5, 122.9, 91.5. IR (KBr): 2386, 2341, 1667, 1610, 1568, 1463, 837 cm^{-1}.

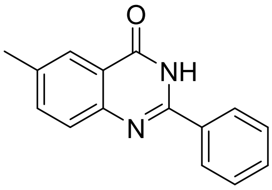

6-Methyl-2-phenylquinazolin-4(3*H*)-one (6f)[4]

Yellow solid (70.2 mg, 99%); m.p.=265-267 °C. ^{1}H NMR (400 MHz, DMSO-d_6) δ 12.40 (s, 1H), 8.17 (d, *J* = 6.9 Hz, 2H), 7.95 (s, 1H), 7.65 (s, 2H), 7.60 – 7.52 (m, 3H), 2.46 (s, 3H). ^{13}C NMR (100 MHz, DMSO-d_6) δ 162.5, 151.8, 147.0, 136.7, 136.2, 133.0, 131.5, 128.9, 127.9, 127.6, 125.5, 120.9, 21.1. IR (KBr): 2367, 2334, 1667, 1599, 1566, 1090, 830, 696 cm^{-1}.

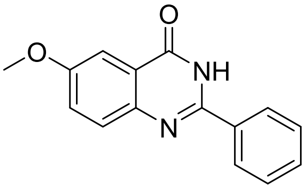

6-Methoxy-2-phenylquinazolin-4(3*H*)-one (6g)[4]

White solid (70.4 mg, 93%); m.p.=246-247 °C. ^{1}H NMR (400 MHz, DMSO-d_6) δ 12.49 (br, 1H), 8.16 (d, *J* = 8.0 Hz, 2H), 7.70 (d, *J* = 8.0 Hz, 1H), 7.55 – 7.53 (m, 4H), 7.46 –7.43 (m, 1H), 3.89 (s, 3H). ^{13}C NMR (100 MHz, DMSO-d_6) δ 162.2, 157.9, 150.3, 143.4, 132.9, 131.3, 129.4, 128.8, 127.7, 124.3, 121.9, 106.0, 55.8. IR(KBr): 2368, 2333, 1668, 1600, 1564, 1532, 1485, 861 cm^{-1}.

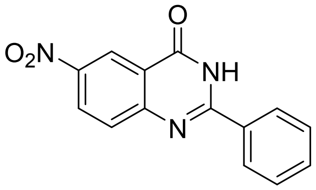

6-Nitro-2-phenylquinazolin-4(3*H*)-one (6h)[4]

White solid (73.0 mg, 91%); m.p.=235-236 °C. ^{1}H NMR (400 MHz, DMSO-d_6) δ 12.76 (br, 1H), 8.26 (d, J = 8.1 Hz, 2H), 8.15 (d, J = 8.0 Hz, 2H), 7.62 – 7.56 (m, 3H), 7.41 (t, J = 7.8 Hz, 1H). ^{13}C NMR (100 MHz, DMSO-d_6) δ 162.0, 153.0, 146.2, 138.0, 132.5, 131.9, 128.8, 128.0, 127.4, 125.8, 122.7, 122.3. IR (KBr): 2379, 2343, 1677, 1568, 1542, 1512, 1475, 761 cm^{-1}.

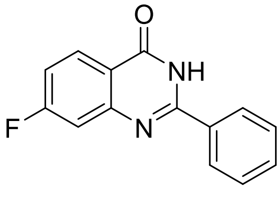

7-Fluoro-2-phenylquinazolin-4(3*H*)-one (6i)[7]
White solid (71.3 mg, 99%); m.p.=255-256 °C. ^{1}H NMR (400 MHz, DMSO-d_6) δ 12.63 (br, 1H), 8.25 – 8.13 (m, 3H), 7.64 – 7.48 (m, 4H), 7.42 – 7.34 (m, 1H). ^{13}C NMR (100 MHz, DMSO-d_6) δ 165.9 (d, J = 250 Hz), 161.6, 153.8, 151.0 (d, J = 14 Hz), 132.5, 131.7, 129. 0 (d, J = 11 Hz), 128.7, 128.0, 118.1, 115.2 (d, J = 23 Hz), 112.6 (d, J = 22 Hz). IR (KBr): 2382, 2343, 1679, 1647, 1457, 1313, 770 cm^{-1}.

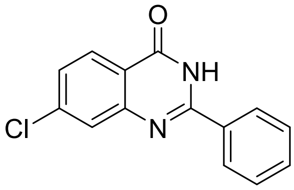

7-Chloro-2-phenylquinazolin-4(3*H*)-one (6j)[4]
White solid (66.2 mg, 86%); m.p.=277-278 °C. ^{1}H NMR (400 MHz, DMSO-d_6) δ 12.66 (br, 1H), 8.18 –8.12 (m, 3H), 7.77 (d, J = 1.8 Hz, 1H), 7.62 – 7.52 (m, 4H). ^{13}C NMR (100 MHz, DMSO-d_6) δ 161.7, 153.9, 150.0, 139.3, 132.5, 131.8, 128.7, 128.0, 126.9, 126.7, 119.9. IR (KBr): 2378, 2340, 1670, 1586, 1548, 1469, 1099, 698 cm^{-1}.

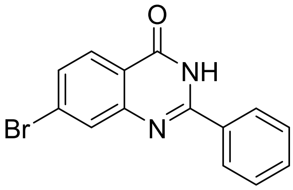

7-Bromo-2-phenylquinazolin-4(3*H*)-one (6k)[5]
White solid (88.5 mg, 98%); m.p.=294-295 °C. ^{1}H NMR (400 MHz, DMSO-d_6) δ 12.67 (br, 1H), 8.17 (d, J = 7.2 Hz, 2H), 8.06 (d, J = 8.5 Hz, 1H), 7.94 (d, J = 1.6 Hz, 1H), 7.67 (dd, J = 8.5, 1.7 Hz, 1H), 7.63 –7.54 (m, 3H). ^{13}C NMR (100 MHz, DMSO-d_6) δ 161.9, 153.8, 150.0, 132.5, 131.8, 129.8, 129.6, 128.7, 128.2, 128.0, 120.2. IR (KBr): 2379, 2342, 1672, 1625, 1572, 1509, 708 cm^{-1}.

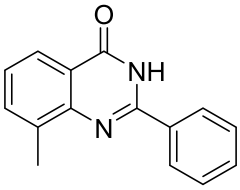

8-Methyl-2-phenylquinazolin-4(3*H*)-one (6l)[7]
White solid (65.2 mg, 92%); m.p.=256-257 °C. ^{1}H NMR (400 MHz, DMSO-d_6) δ 12.52 (br, 1H), 8.23 (d, J = 6.6 Hz, 2H), 7.99 (d, J = 7.6 Hz, 1H), 7.69 (d, J = 6.8 Hz, 1H), 7.57 –7.55 (m, 3H), 7.39 (t, J = 7.4 Hz, 1H), 2.62 (s, 3H). ^{13}C NMR (100 MHz, DMSO-d_6) δ 162.6, 151.1, 147.2, 135.7, 135.0, 133.0, 131.4, 128.7, 127.8, 126.1, 123.6, 121.0, 17.2. IR (KBr): 2379, 2348, 1678, 1618, 1578, 1549, 1459, 764 cm^{-1}.

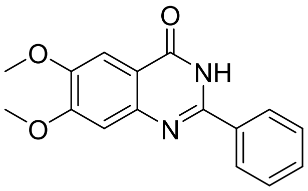

6,7-Dimethoxy-2-phenylquinazolin-4(3*H*)-one (6m)[8]

White solid (83.8 mg, 99%); m.p.=262-263 °C. ^{1}H NMR (400 MHz, DMSO-d_6) δ 12.40 (br, 1H), 8.16 (d, *J* = 6.4 Hz, 2H), 7.59 – 7.46 (m, 4H), 7.21 (s, 1H), 3.93 (s, 3H), 3.89 (s, 3H). ^{13}C NMR (100 MHz, DMSO-d_6) δ 161.7, 154.9, 150.9, 148.7, 144.9, 132.9, 131.1, 128.7, 127.5, 114.1, 108.4, 105.1, 56.1, 55.8. IR (KBr): 2379, 2333, 1659, 1561, 1502, 1447, 1283, 771 cm^{-1}.

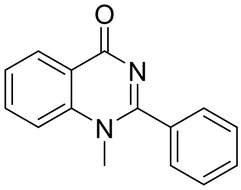

1-Methyl-2-phenylquinazolin-4(1*H*)-one (6n)[9]

Yellow solid (70.1 mg, 99%); m.p.=153-154 °C. ^{1}H NMR (400 MHz, DMSO-d_6) δ 8.15 (d, *J* = 7.9 Hz, 1H), 7.90 (t, *J* = 8.0 Hz, 1H), 7.77 (d, *J* = 8.5 Hz, 1H), 7.69 (dd, *J* = 7.3, 2.1 Hz, 2H), 7.62 – 7.53 (m, 4H), 3.65 (s, 3H). ^{13}C NMR (100 MHz, DMSO-d_6) δ 167.9, 162.2, 141.9, 135.3, 134.1, 130.4, 128.8, 128.6, 127.2, 126.2, 119.9, 117.0, 38.0. IR (KBr): 2372, 2332, 1644, 1601, 1532, 1487, 1394, 764 cm^{-1}.

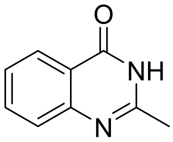

2-Methylquinazolin-4(3*H*)-one (6o)[5]

White soild (46.6 mg, 97%); m.p.=234-235 °C. ^{1}H NMR (400 MHz, DMSO-d_6) δ12.18 (br, 1H), 8.07 (d, *J* = 7.6 Hz, 1H), 7.75 (t, *J* = 7.6 Hz, 1H), 7.56 (d, *J* = 8.0 Hz, 1H), 7.74 (t, *J* = 7.6 Hz, 1H), 2.34 (s, 3H). ^{13}C NMR (100 MHz, DMSO-d_6) δ 161.7, 154.3, 149.0, 134.3, 126.6, 125.9, 125.7, 120.7, 21.5. IR (KBr): 2375, 2338, 1666, 1626, 1474, 1145, 778 cm^{-1}.

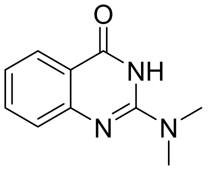

2-(Dimethylamino)quinazolin-4(3*H*)-one (6p)[10]

White solid (46.0 mg, 81%); m.p.=237-238 °C. ^{1}H NMR (400 MHz, DMSO-d_6) δ 11.12 (br, 1H), 7.89 (d, *J* = 7.2 Hz, 1H), 7.55 (t, *J* = 7.1 Hz, 1H), 7.25 – 7.08 (m, 2H), 3.08 (s, 6H). ^{13}C NMR (100 MHz, DMSO-d_6) δ 163.1, 151.3, 151.0, 134.3, 126.0, 124.6, 121.6, 116.4, 37.5. IR (KBr): 2394, 2347, 1697, 1602, 1555, 1156, 756 cm^{-1}.

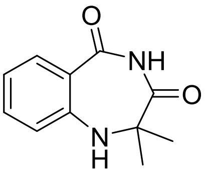

2,2-Dimethyl-1,2-dihydro-3H-benzo[e][1,4]diazepine-3,5(4*H*)-dione (8)[2]

Light yellow solid (43.5 mg, 71%); m.p.=167-168 °C. ^{1}H NMR (400 MHz, DMSO-d_6) δ 10.70 (br, 1H), 7.97 (d, *J* = 8.1 Hz, 1H), 7.37 (t, *J* = 7.5 Hz, 1H), 7.07 (d, *J* = 8.3 Hz, 1H), 6.85 (t, *J* = 7.5 Hz, 1H), 6.60 (br, 1H), 1.30 (s, 6H). ^{13}C NMR (100 MHz, DMSO) δ 173.8, 165.2, 146.9, 134.2, 132.0, 120.2, 118.8, 117.0, 56.7, 23.6. IR (KBr): 3334, 3216, 1687, 1643, 1604, 1484, 1326, 757 cm^{-1}.

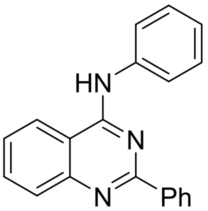

***N*,2-Diphenylquinazolin-4-amine (9a)**[11]

Yellow solid (66.9 mg, 75%); m.p.=153-154 ^{o}C. ^{1}H NMR (400 MHz, $CDCl_3$) δ 8.55 (d, *J* = 7.8 Hz, 2H), 8.01 (d, *J* = 7.8 Hz,, 1H), 7.94 – 7.87 (m, 3H), 7.77 (t, *J* = 7.4 Hz, 1H), 7.61 (br, 1H), 7.55 – 7.41 (m, 6H), 7.20 (t, *J* = 7.4 Hz, 1H). ^{13}C NMR (100 MHz, $CDCl_3$) δ 160.3, 157.3, 150.8, 138.6, 132.9, 130.3, 129.1, 129.0, 128.5, 128.4, 126.1, 124.1, 121.3, 120.3, 113.8. IR (KBr): 2380, 2334, 1696, 1566, 1528, 1445, 756, 711 cm^{-1}.

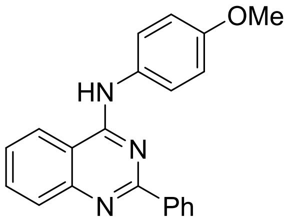

***N*-(4-methoxyphenyl)-2-phenylquinazolin-4-amine (9b)[11]**

Brown solid (76.6 mg, 78%); m.p.=167-168 ^{o}C. ^{1}H NMR (400 MHz, $CDCl_3$) δ 8.52 (d, *J* = 7.1 Hz, 2H), 7.97 (d, *J* = 8.0 Hz, 1H), 7.83 (d, *J* = 7.7 Hz, 1H), 7.74 –7.72 (m, 3H), 7.48– 7.46 (m, 5H), 6.98 (d, *J* = 8.0 Hz, 2H), 3.85 (s, 3H). ^{13}C NMR (100 MHz, $CDCl_3$) δ 160.3, 157.5, 156.4, 150.7, 138.5, 132.7, 131.5, 130.2, 128.9, 128.4, 128.3, 125.9, 123.4, 120.3, 114.1, 113.7, 55.5. IR (KBr): 2370, 2341, 1570, 1524, 1424, 1377, 1245, 764 cm^{-1}.

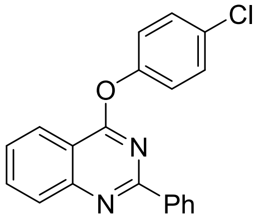

4-(4-Chlorophenoxy)-2-phenylquinazoline (10a)

White solid (76.9 mg, 77%); m.p.=176-177 ^{o}C. ^{1}H NMR (400 MHz, $CDCl_3$) δ 8.37 –8.34 (m, 3H), 8.11 (d, *J* = 8.3 Hz, 1H), 7.91 (t, *J* = 7.6 Hz, 1H), 7.62 (t, *J* = 7.5 Hz, 1H), 7.52 – 7.42 (m, 5H), 7.33 (d, *J* = 8.7 Hz, 2H). ^{13}C NMR (100 MHz, $CDCl_3$) δ 166.5, 159.7, 152.4, 151.1, 137.2, 134.3, 130.9, 130.8, 129.5, 128.5, 128.0, 127.1, 123.5, 123.4, 114.9. IR (KBr): 2377, 2344, 1483, 1381, 1214, 764, 705 cm^{-1}. HRMS (ESI) m/z: $[M+H]^+$ Calcd for $C_{20}H_{14}ClN_2O$ 333.0789; Found 333.0781.

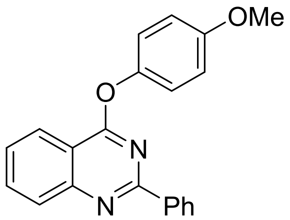

4-(4-Methoxyphenoxy)-2-phenylquinazoline (10b)

White solid (81.8 mg, 83%); m.p.=135-136 ^{o}C. ^{1}H NMR (400 MHz, $CDCl_3$) δ 8.39 –8.35 (m, 3H), 8.07 (d, *J* = 8.4 Hz, 1H), 7.88 (t, *J* = 7.7 Hz, 1H), 7.60 (t, *J* = 7.5 Hz, 1H), 7.43 –7.42 (m, 3H), 7.29 (d, *J* = 8.4 Hz, 2H), 7.03 (d, *J* = 8.9 Hz, 2H), 3.89 (s, 3H). ^{13}C NMR (100 MHz, $CDCl_3$) δ 159.9, 152.9, 150.0, 145.4, 139.1, 130.6, 126.9, 123.5, 121.4, 121.3, 121.0, 119.8, 116.5, 115.7, 108.1, 107.4, 48.6. IR (KBr): 2376, 2337, 1571, 1484, 1379, 1345, 1199, 765 cm^{-1}. HRMS (ESI) m/z: $[M+H]^+$ Calcd for $C_{21}H_{17}N_2O_2$ 329.1285; Found 329.1278.

References

1. Y. Liu, Z. Wu, Y. Wang, Y. Zhang, J. Shao, T. Lu and W. Li, *J. Org. Chem*., 2020, **85**, 7840.
2. E.Kim, C. Y. Lee, S.-G. Kim, *Adv. Synth. Catal*., 2020, **362**, 3594.
3. A. J. Ansari, A.Yadav, A. Mukherjee, E. Sathish, K. Nagesh and R. Singh, *Chem. Commun*., 2020, **56**, 4804.
4. Y. Hu, S. Li, H. Li, Y. Li, J. Li, C. Duanmu and B. Li, *Org. Chem. Front*., 2019, **6**, 2744.
5. F.-C. Jia, Z.-W. Zhou, C. Xu, Y.-D.Wu and A.-X. Wu, *Org. Lett*., 2016, **18**, 2942.
6. S. Mohammed, R. A.Vishwakarma and S. B. Bharate, *J. Org. Chem*., 2015, **80**, 6915.
7. X. Jiang, T. Tang, J.-M.Wang, Z. Chen, Y.-M. Zhu and S.-J. Ji, *J. Org. Chem*., 2014, **79**, 5082.
8. X. Zhang, D.Ye, H. Sun, D. Guo, J. Wang, H. Huang, X. Zhang, H. Jiang and H. Liu, *Green Chem*., 2009, **11**, 1881.
9. H. Hikawa, Y. Ino, H. Suzuki and Y. Yokoyama, *J. Org. Chem*., 2012, **77**, 7046
10. Y. Xu, Q. Xie, W. Li, H. Sun, Y. Wang and L. Shao, *Tetrahedron*, 2015, **71**, 4853.
11. H. Cope, R. Mutter, W. Heal, C. Pascoe, P. Brown, S. Pratt and B. Chen, *Eur. J. Med. Chem.*, 2006, **41**, 1124.

NMR Spectra for All Compounds

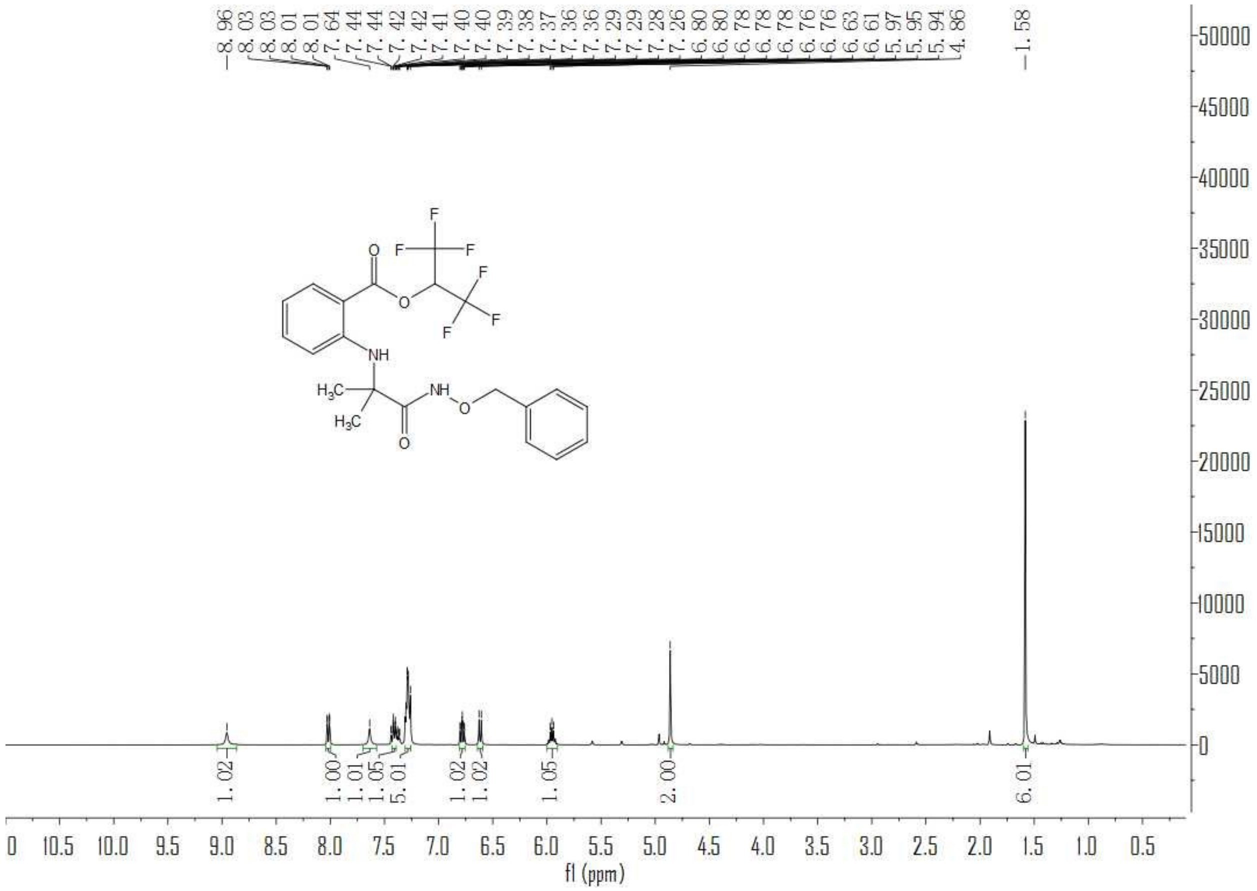

^{1}H NMR spectra (400 MHz, $CDCl_3$) of 3a

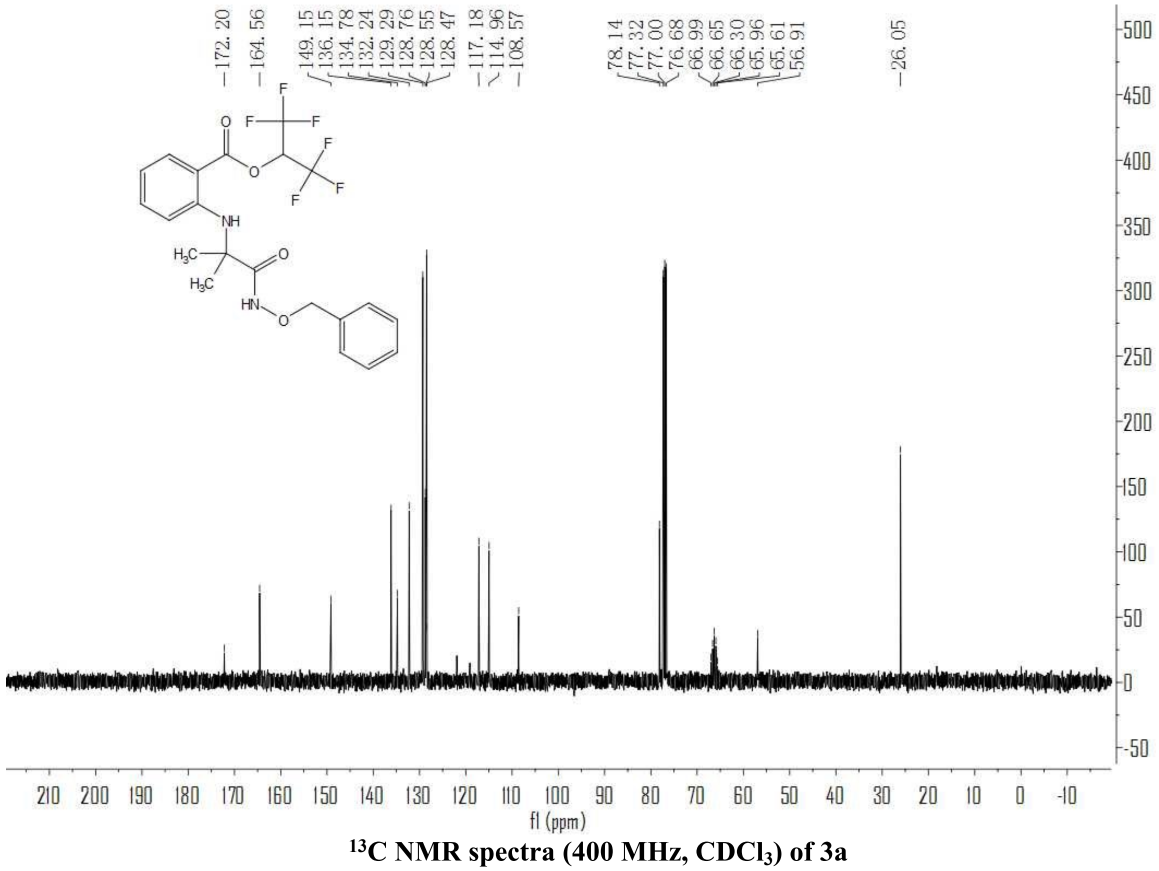

^{13}C NMR spectra (400 MHz, $CDCl_3$) of 3a

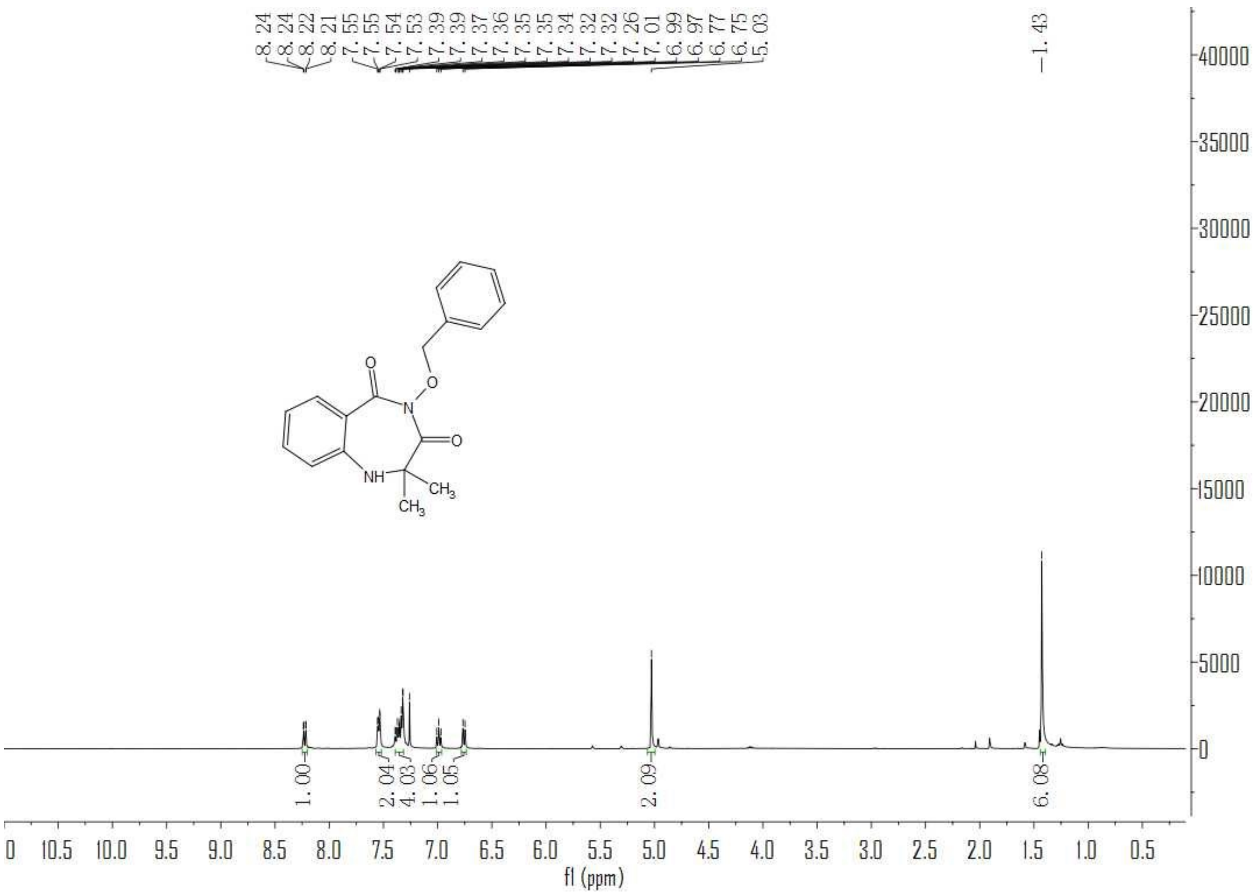

^{1}H NMR spectra (400 MHz, $CDCl_3$) of 4a

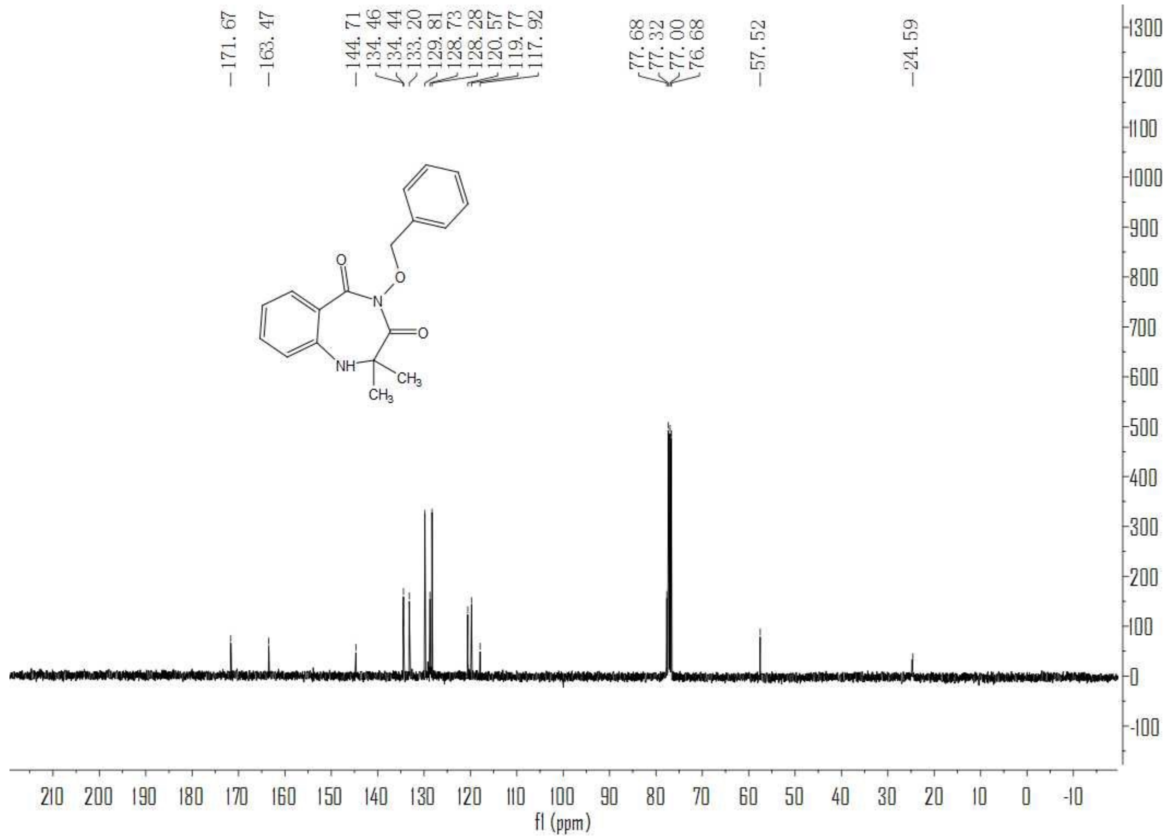

^{13}C NMR spectra (100 MHz, $CDCl_3$) of 4a

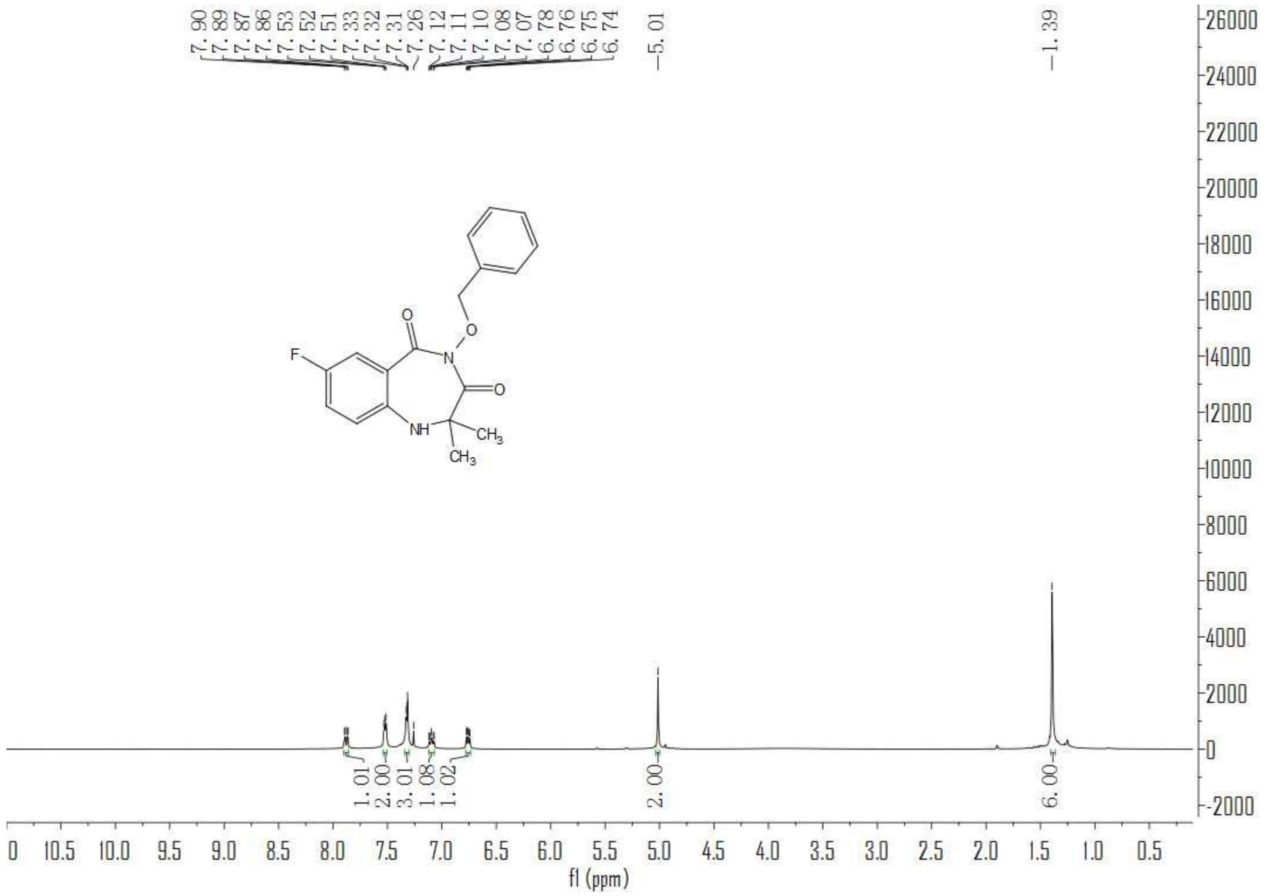

^{1}H NMR spectra (400 MHz, $CDCl_3$) of 4b

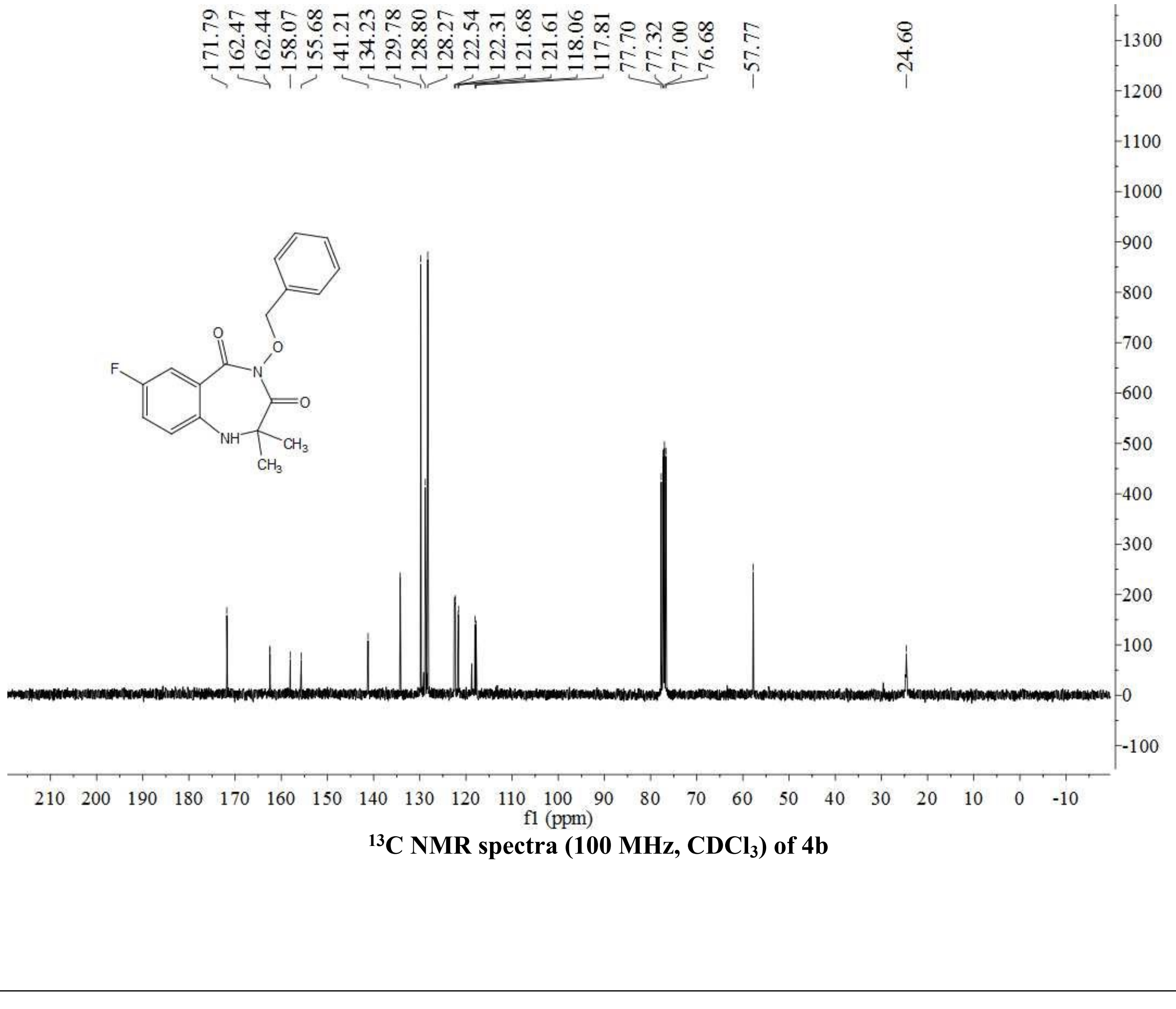

^{13}C NMR spectra (100 MHz, $CDCl_3$) of 4b

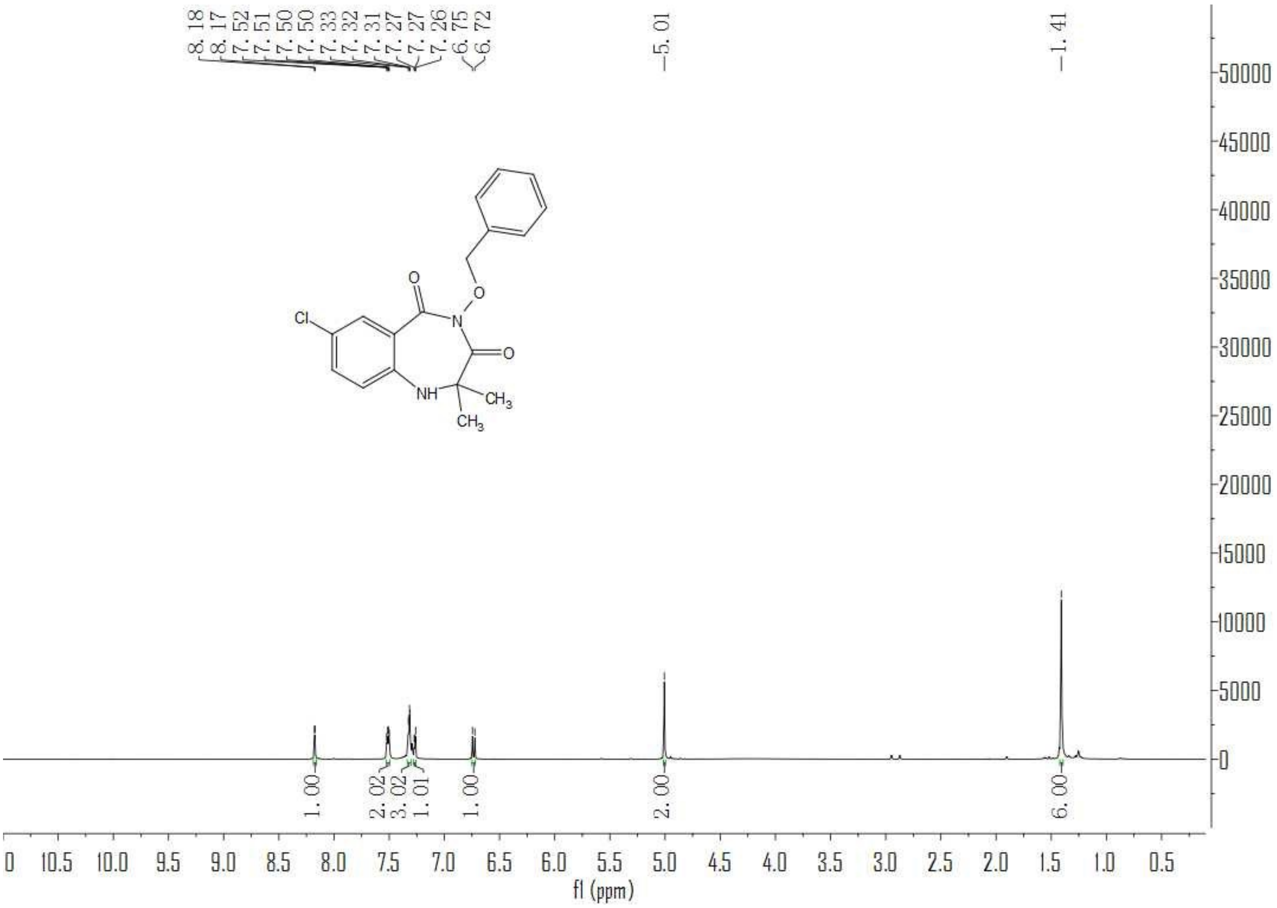

^{1}H NMR spectra (400 MHz, $CDCl_3$) of 4c

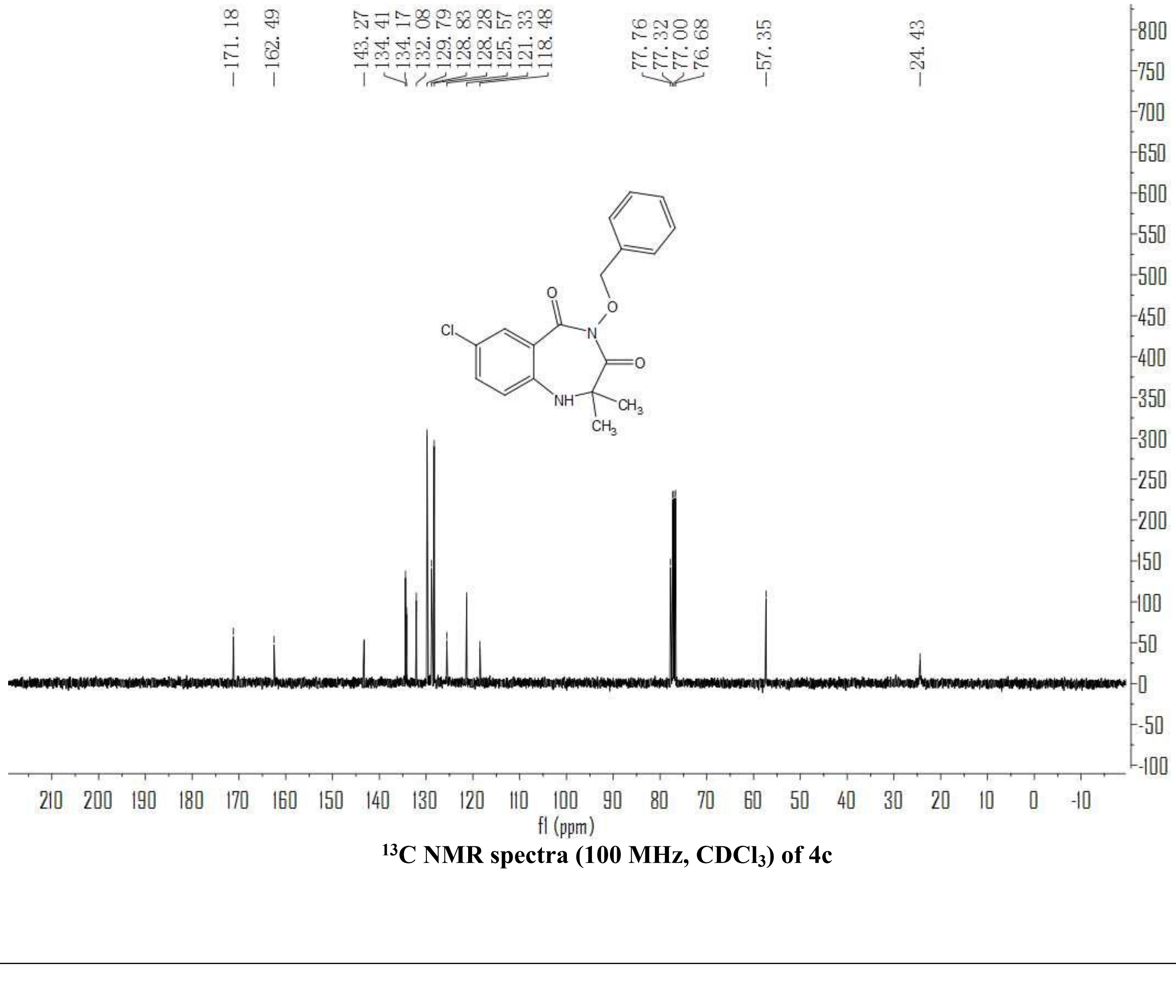

^{13}C NMR spectra (100 MHz, $CDCl_3$) of 4c

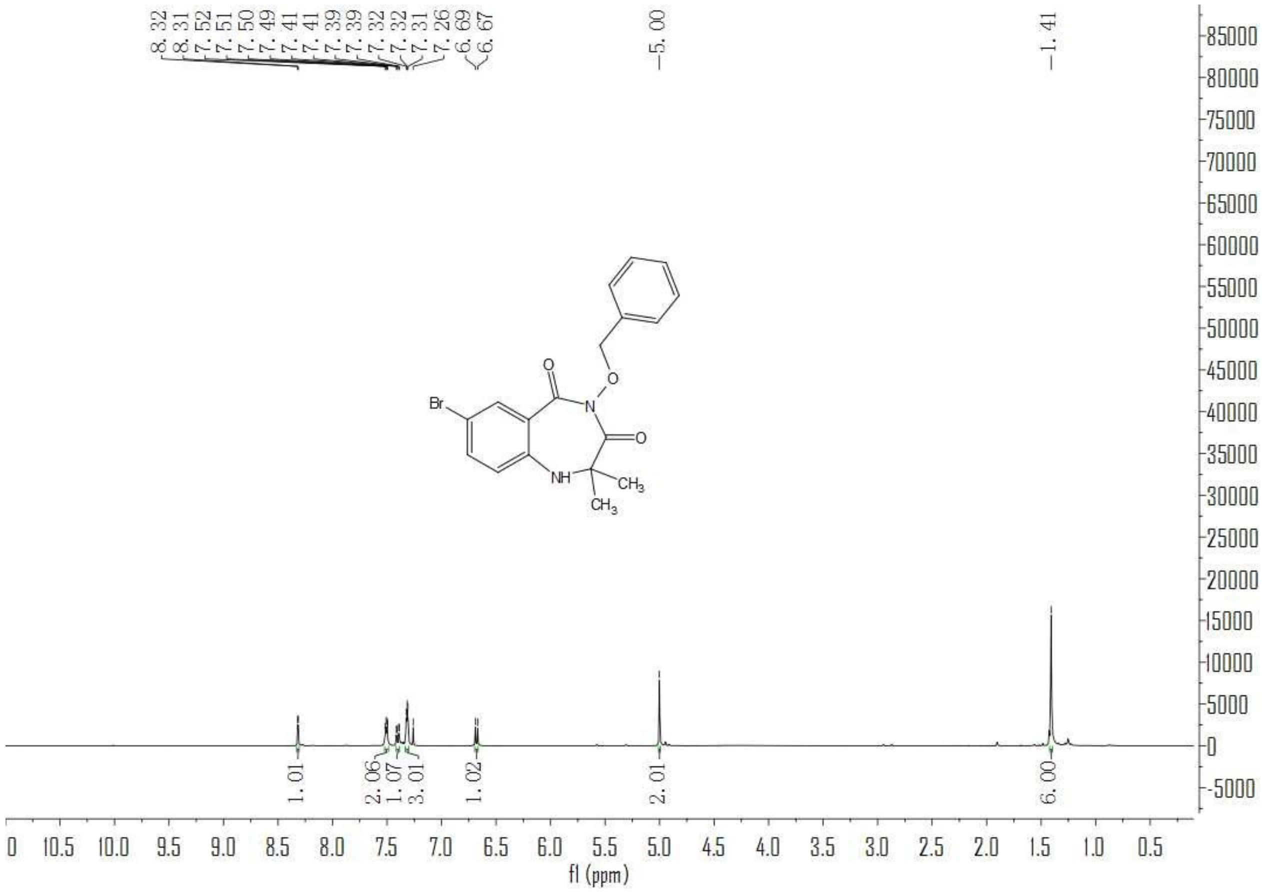

^{1}H NMR spectra (400 MHz, $CDCl_3$) of 4d

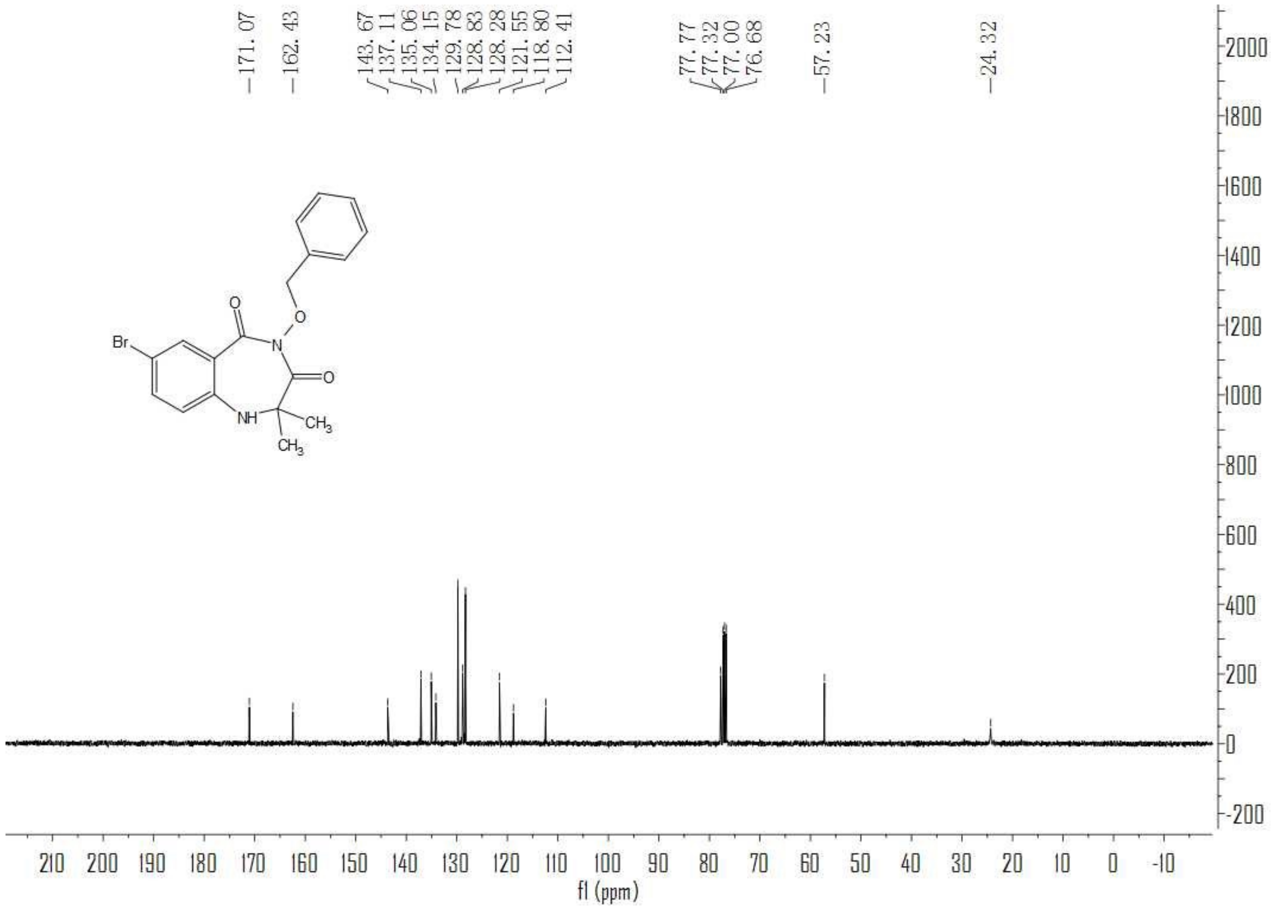

^{13}C NMR spectra (100 MHz, $CDCl_3$) of 4d

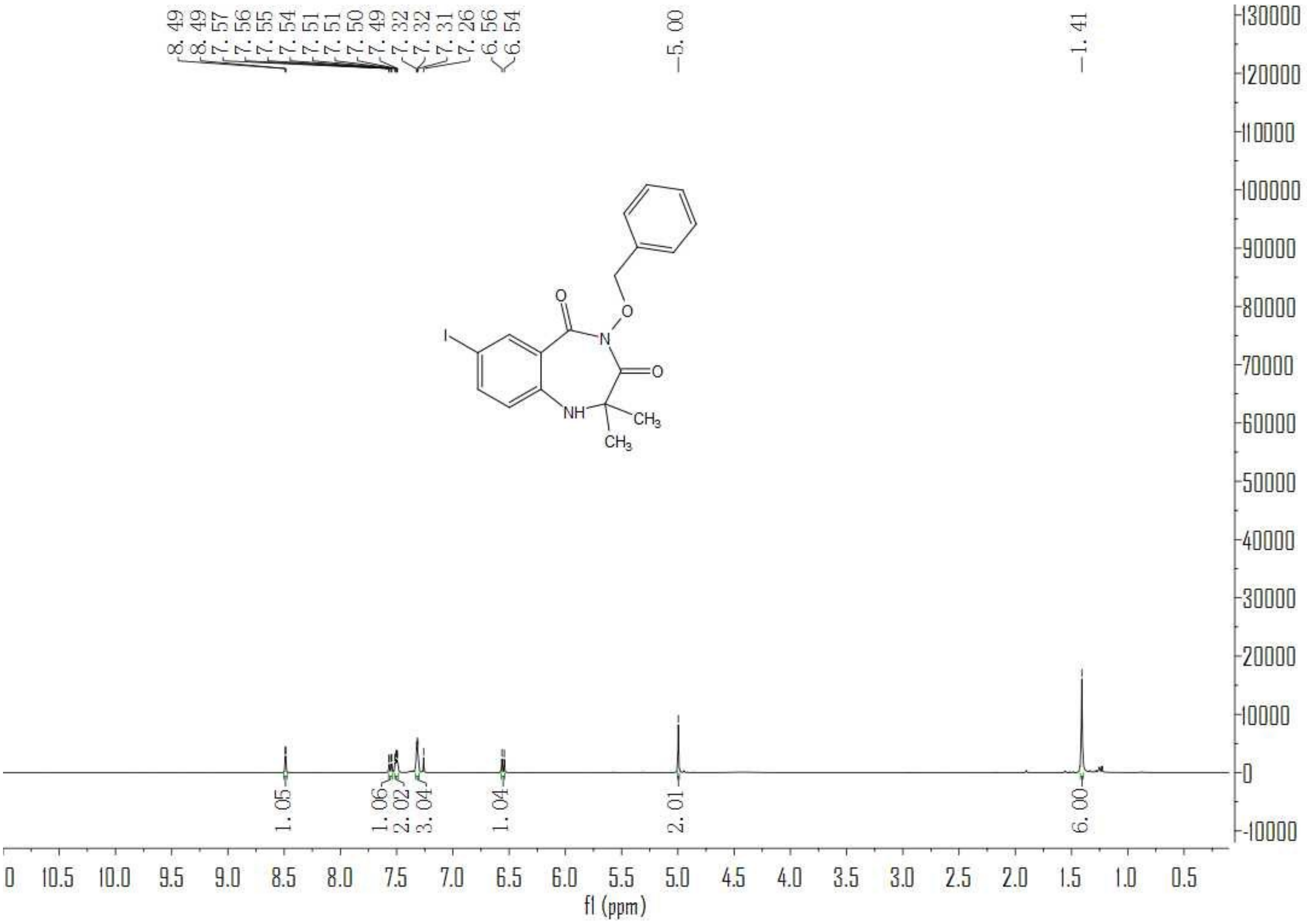

^{1}H NMR spectra (400 MHz, CDCl$_3$) of 4e

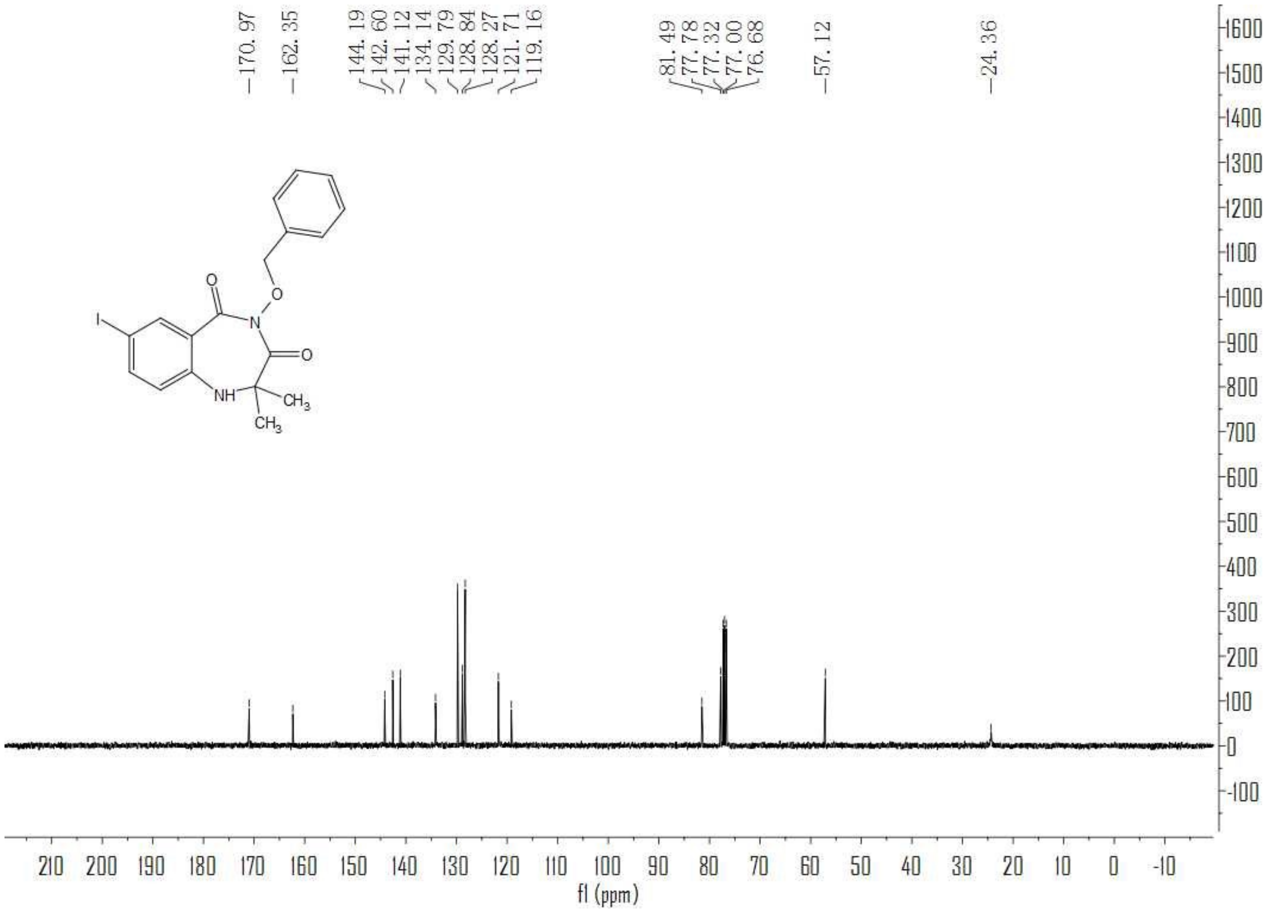

^{13}C NMR spectra (100 MHz, CDCl$_3$) of 4e

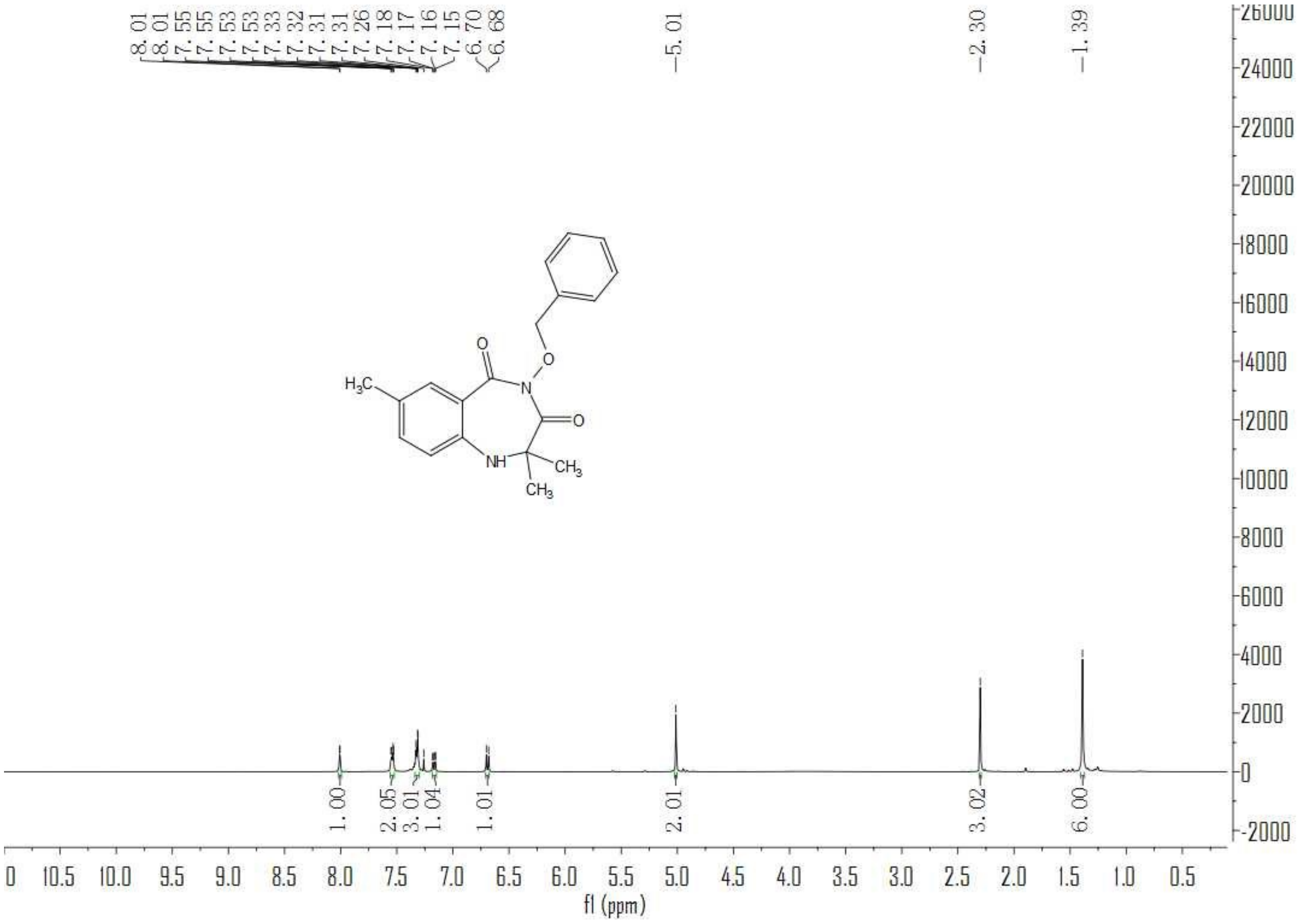

^{1}H NMR spectra (400 MHz, $CDCl_3$) of 4f

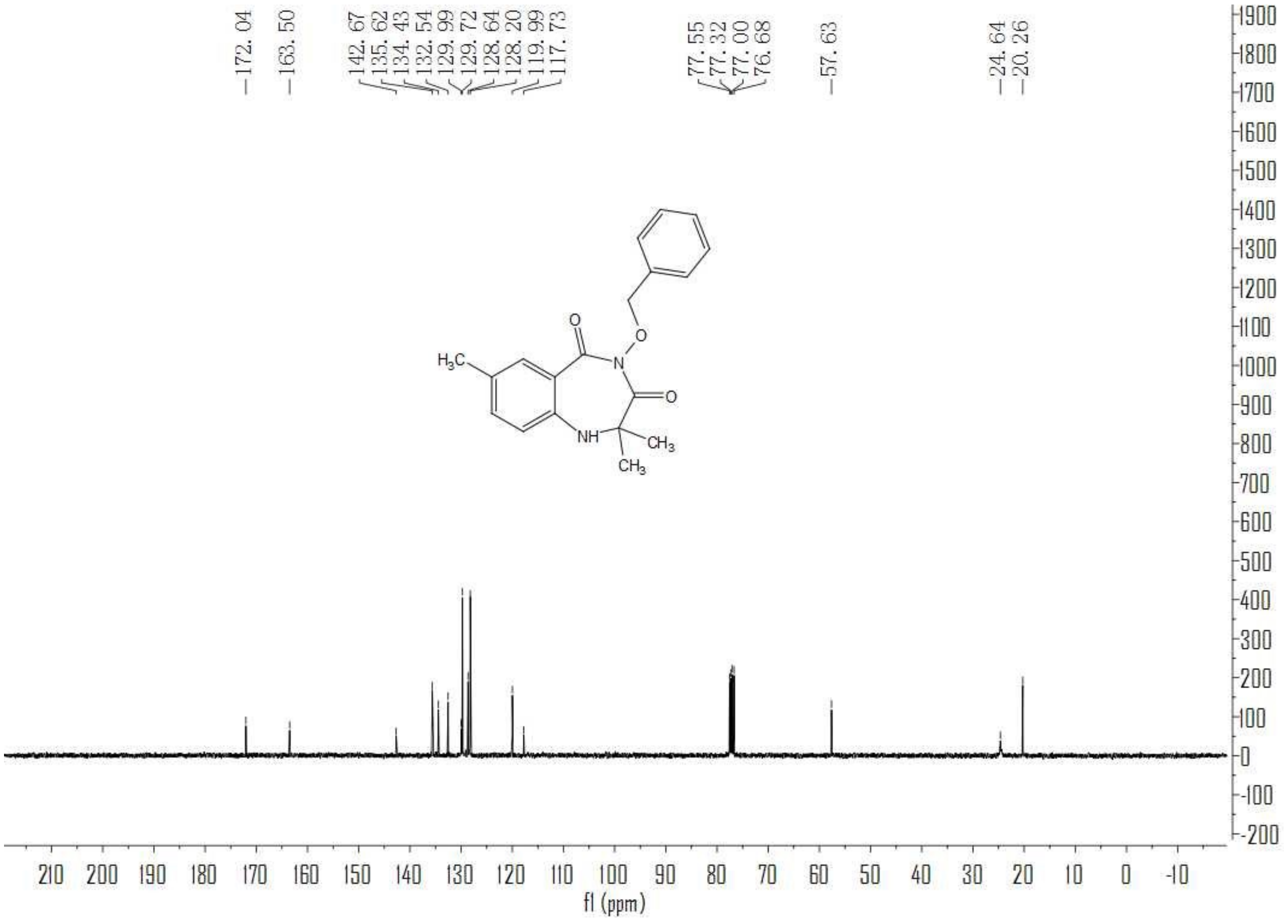

^{13}C NMR spectra (100 MHz, $CDCl_3$) of 4f

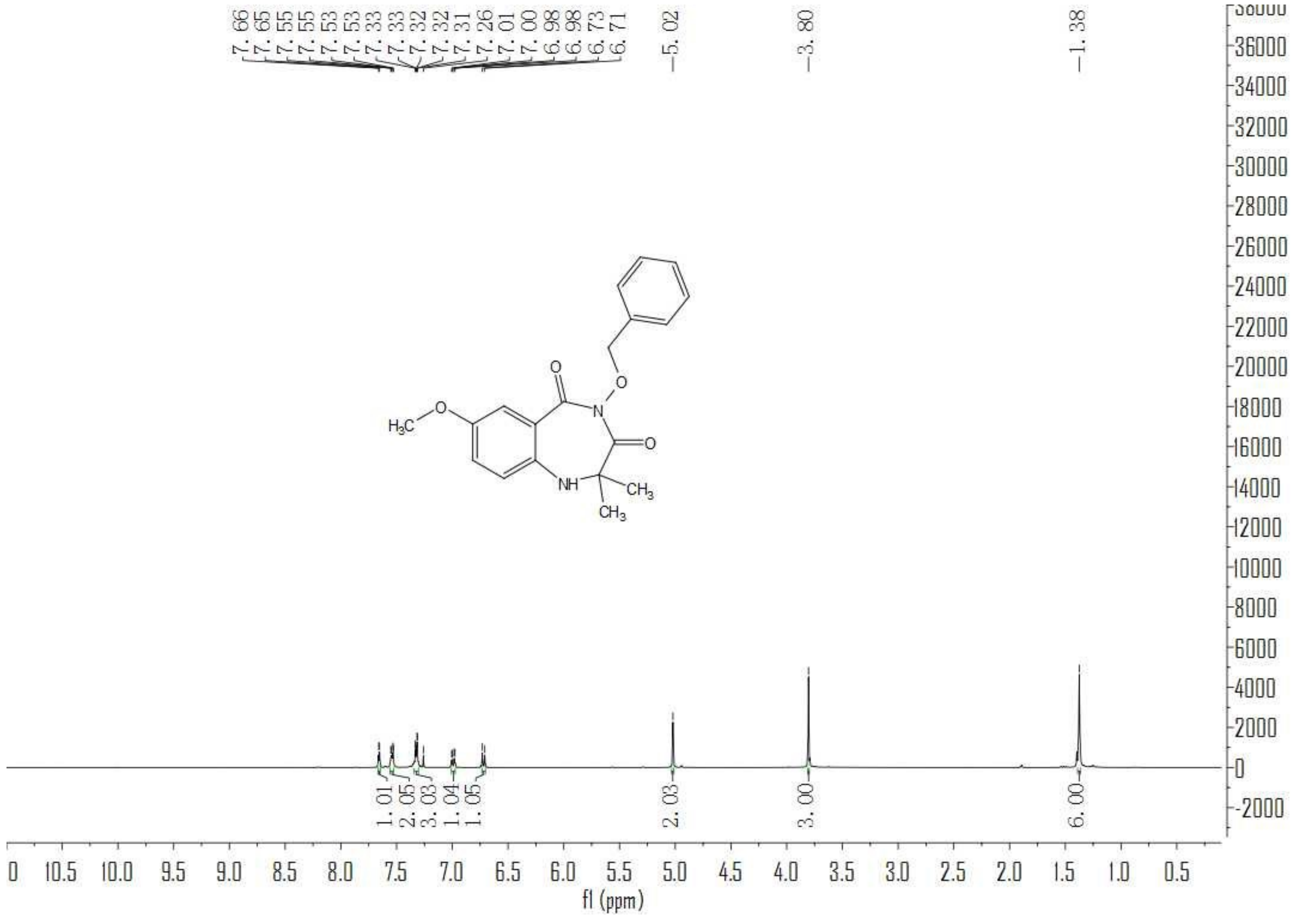

^{1}H NMR spectra (400 MHz, CDCl$_3$) of 4g

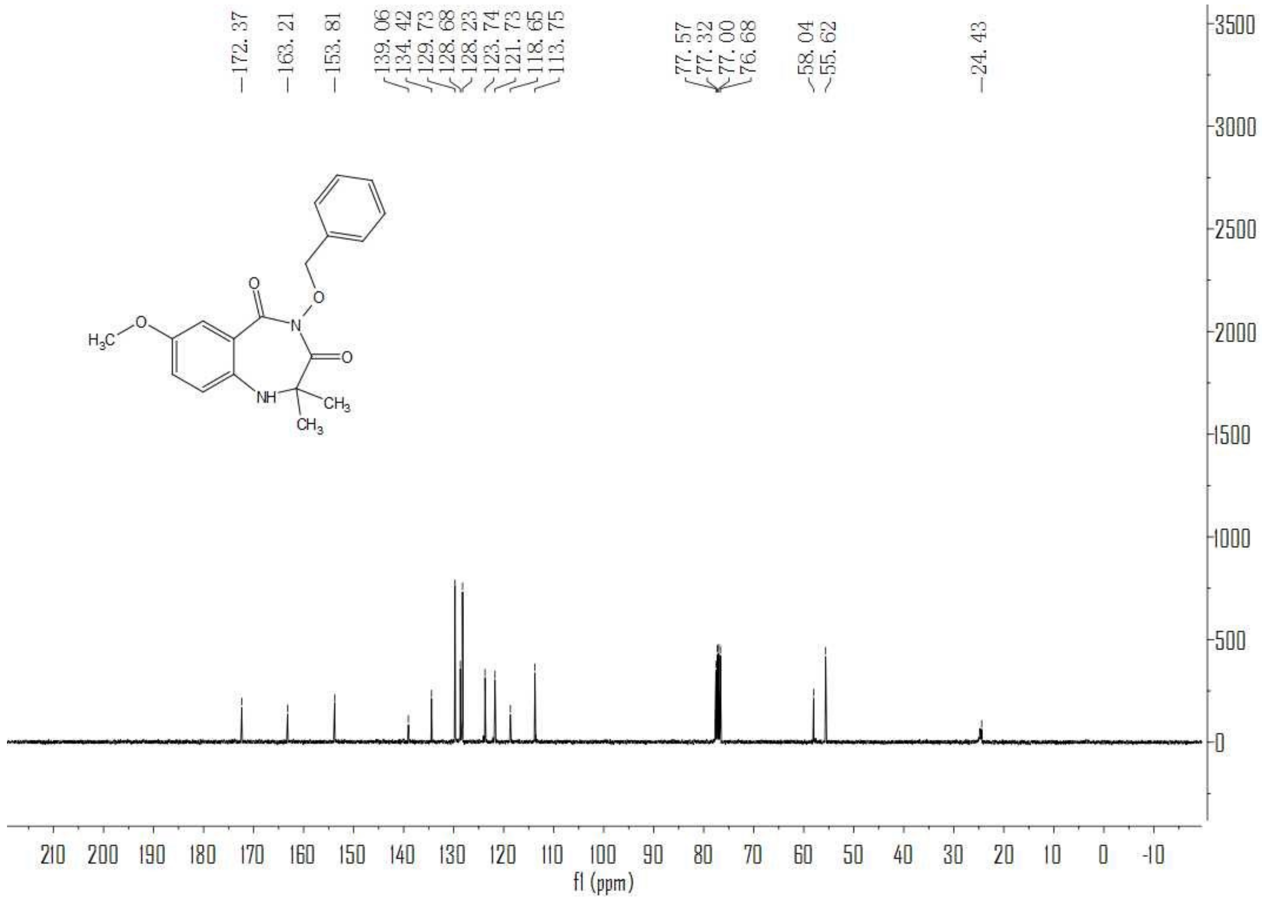

^{13}C NMR spectra (100 MHz, CDCl$_3$) of 4g

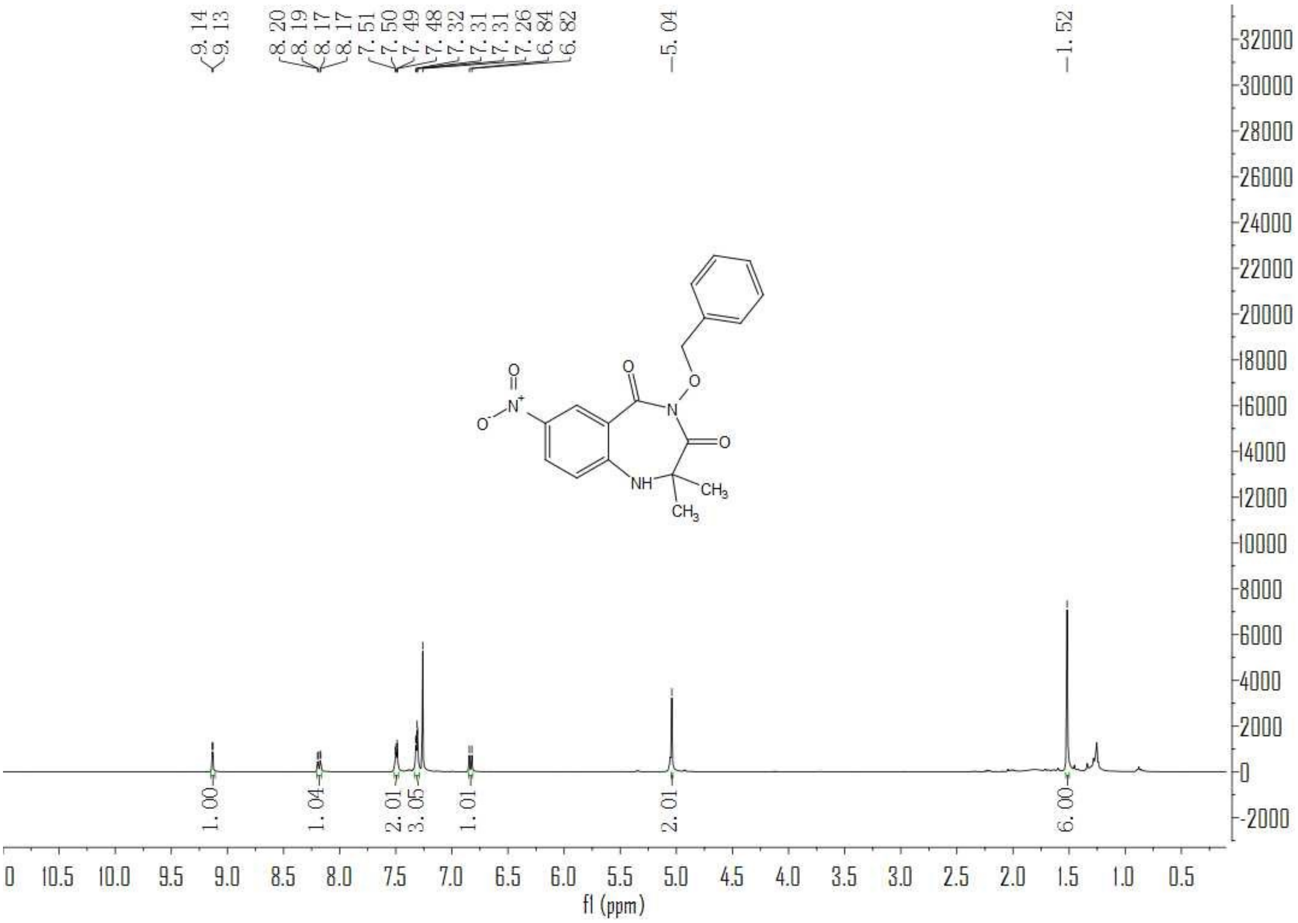

^{1}H NMR spectra (400 MHz, $CDCl_3$) of 4h

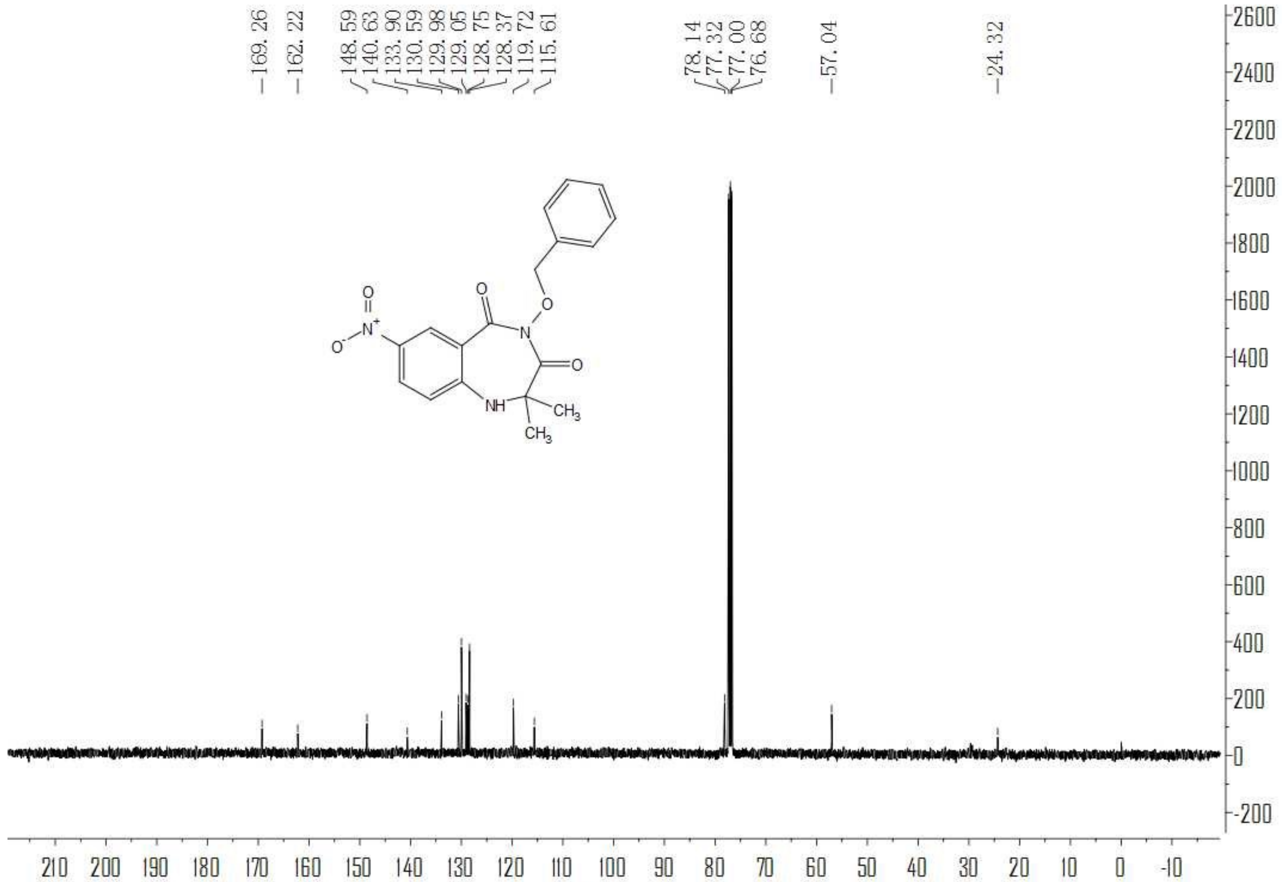

^{13}C NMR spectra (100 MHz, $CDCl_3$) of 4h

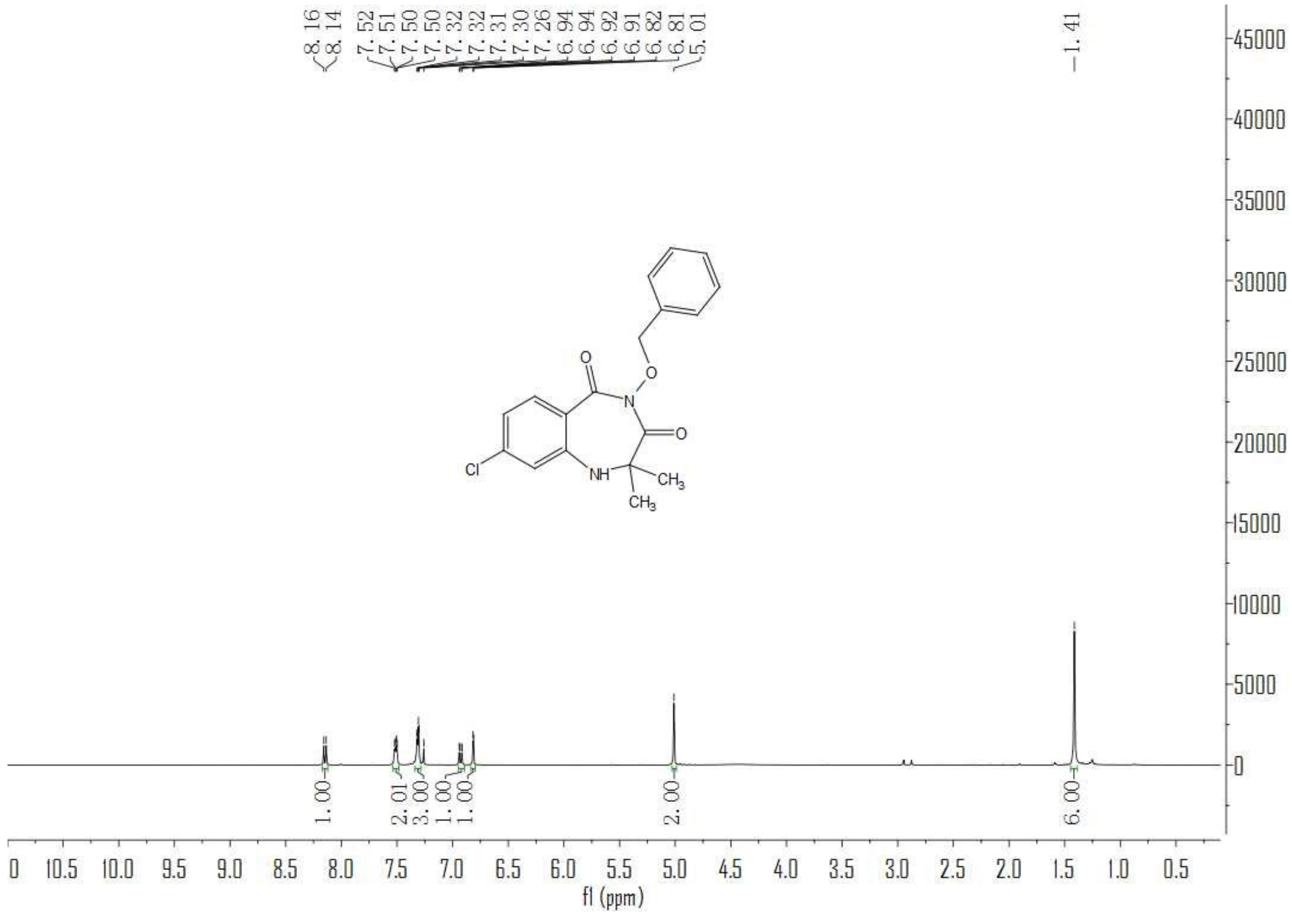

^{1}H NMR spectra (400 MHz, $CDCl_3$) of 4i

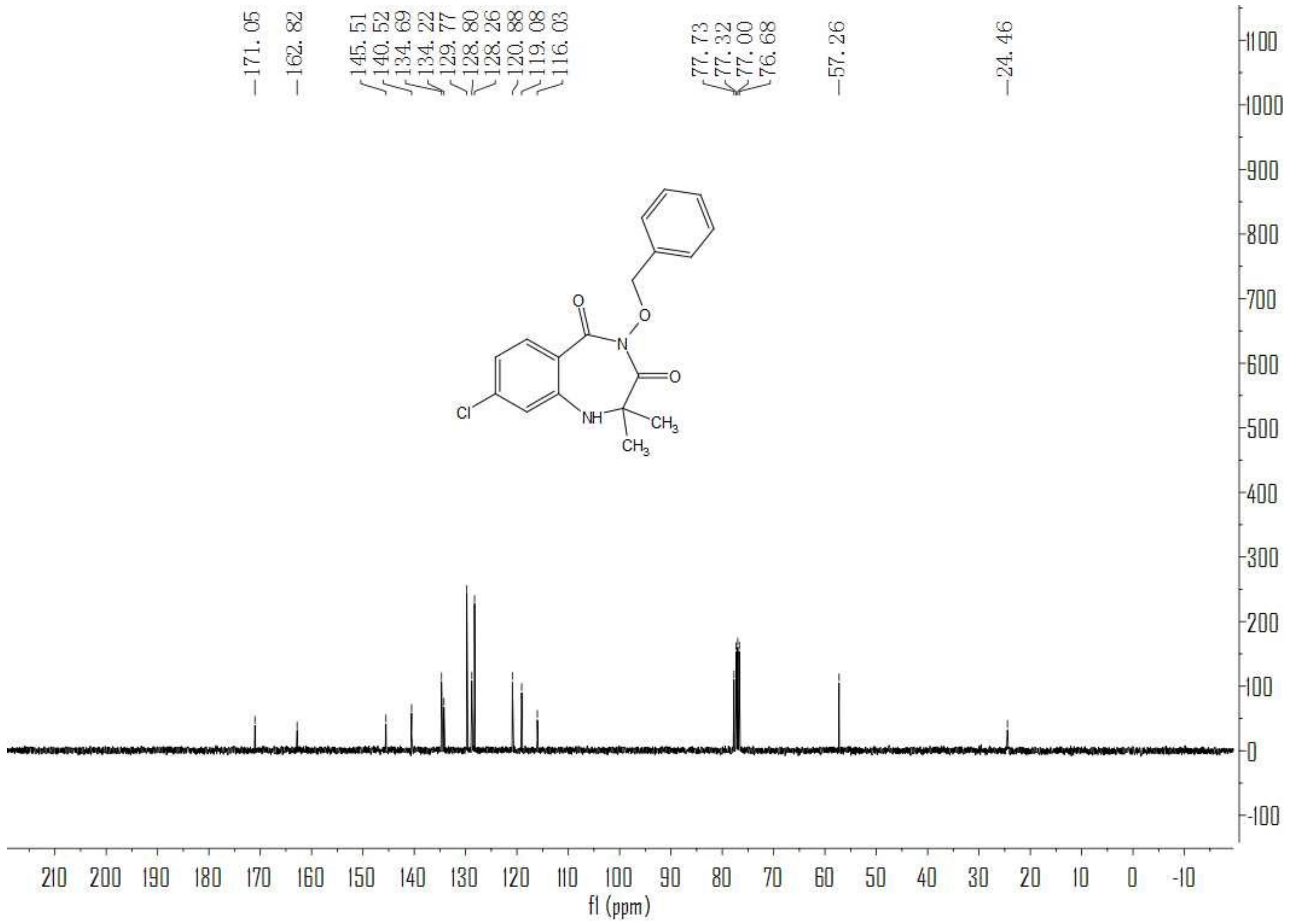

^{13}C NMR spectra (100 MHz, $CDCl_3$) of 4i

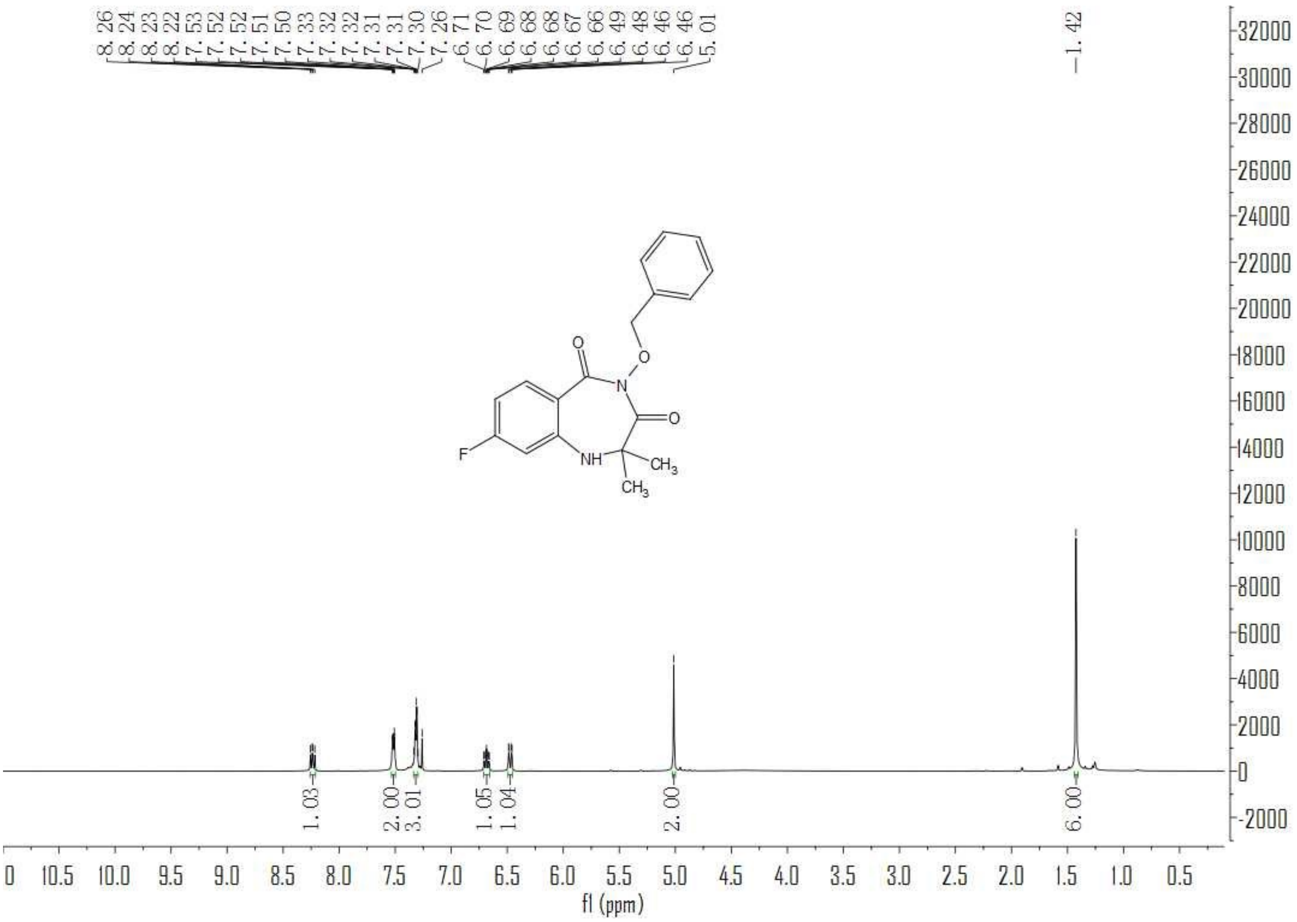

^{1}H NMR spectra (400 MHz, $CDCl_3$) of 4j

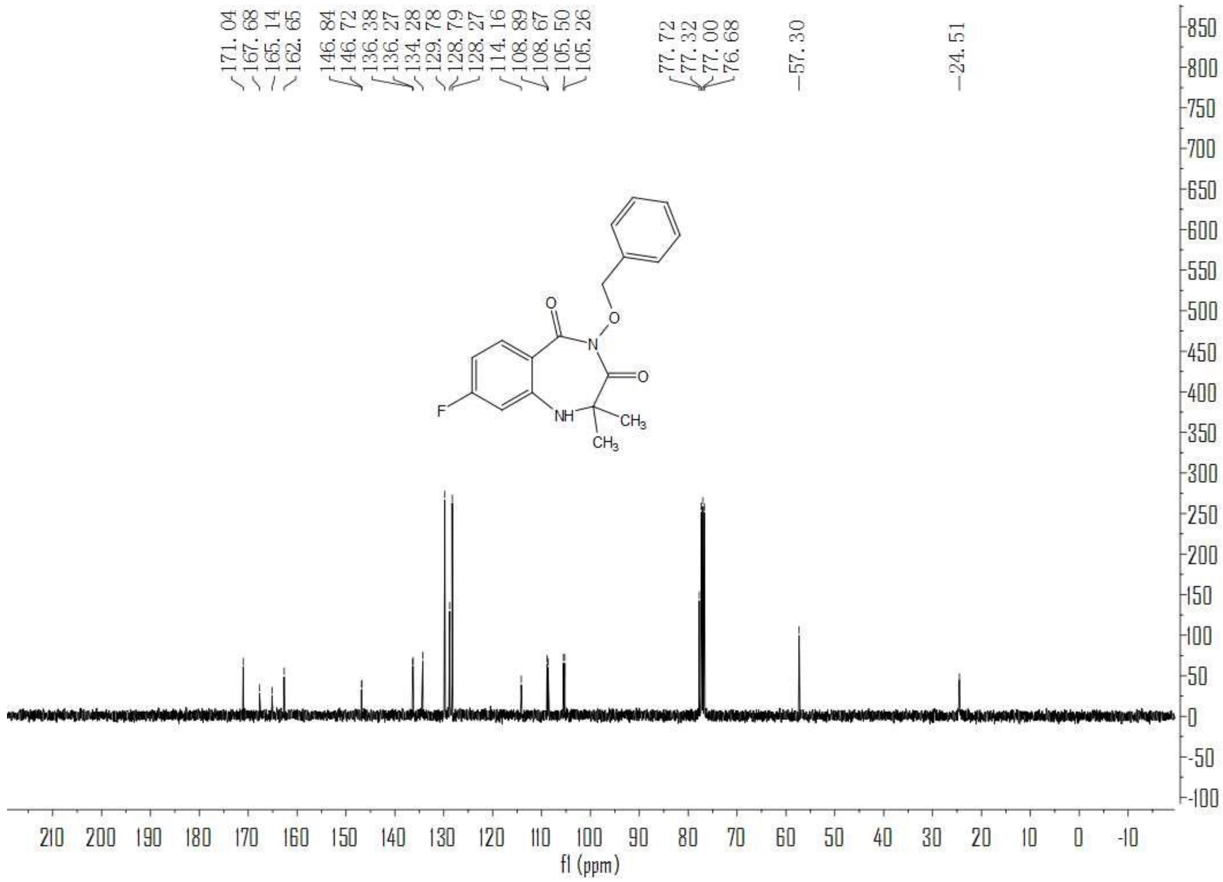

^{13}C NMR spectra (100 MHz, $CDCl_3$) of 4j

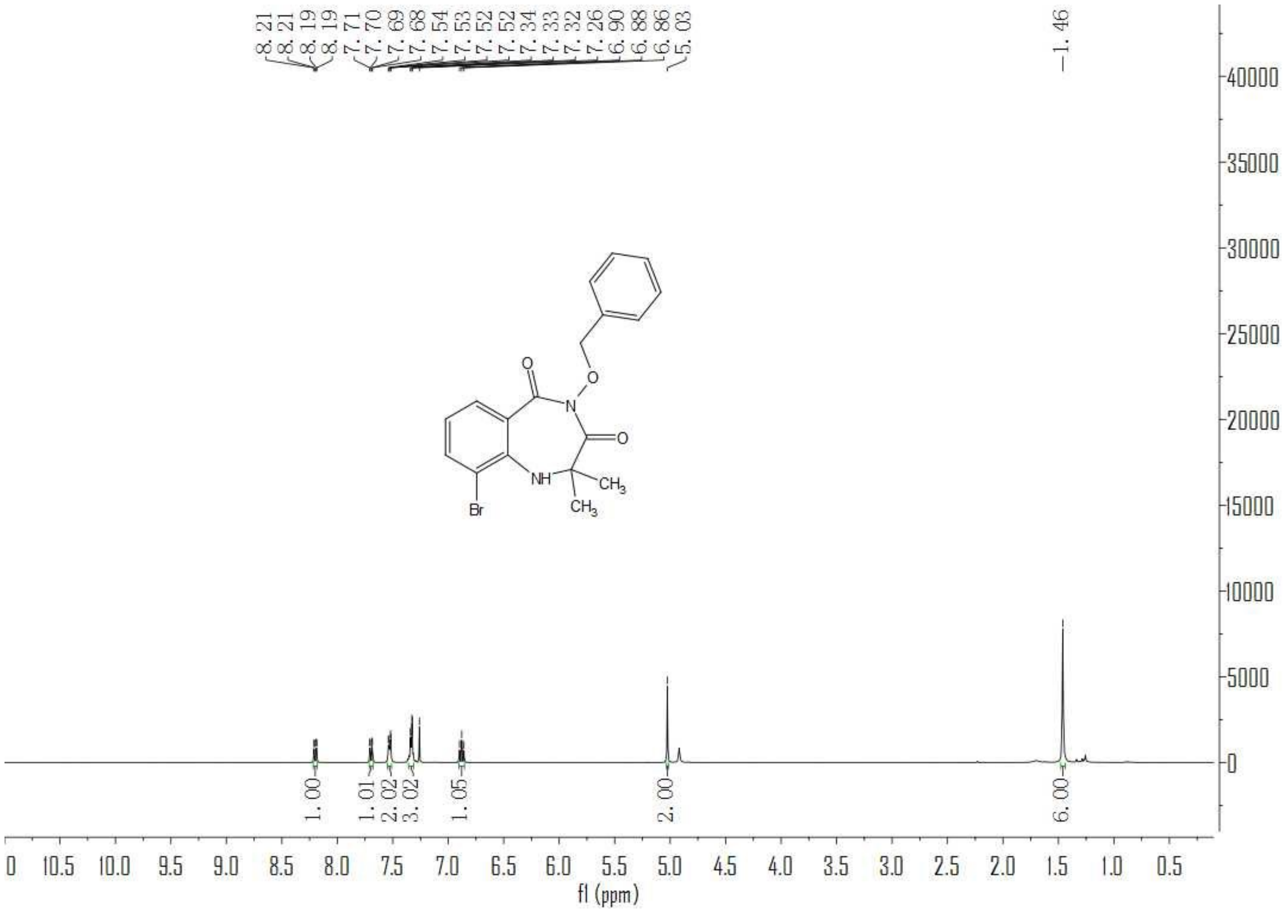

^{1}H NMR spectra (400 MHz, $CDCl_3$) of 4k

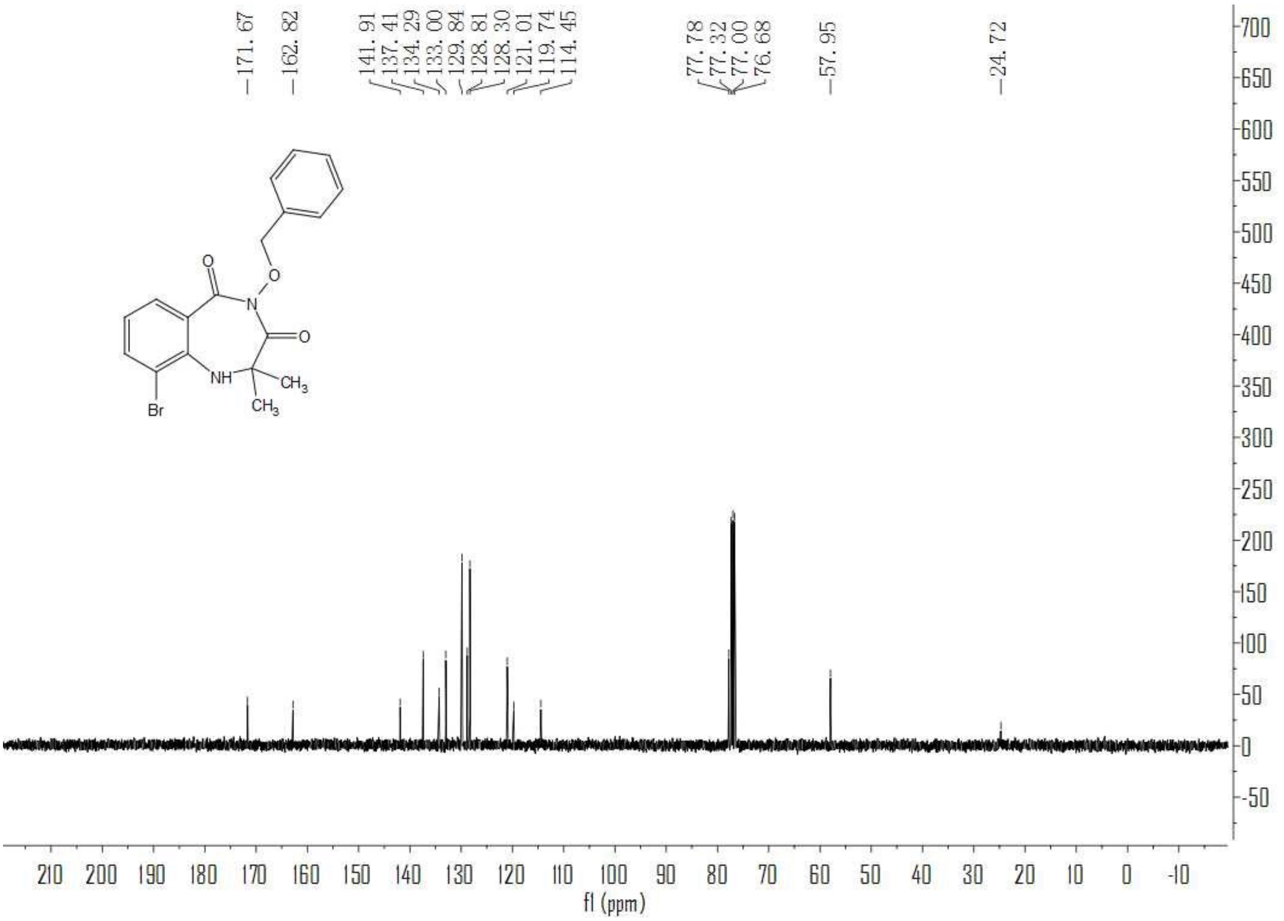

^{13}C NMR spectra (100 MHz, $CDCl_3$) of 4k

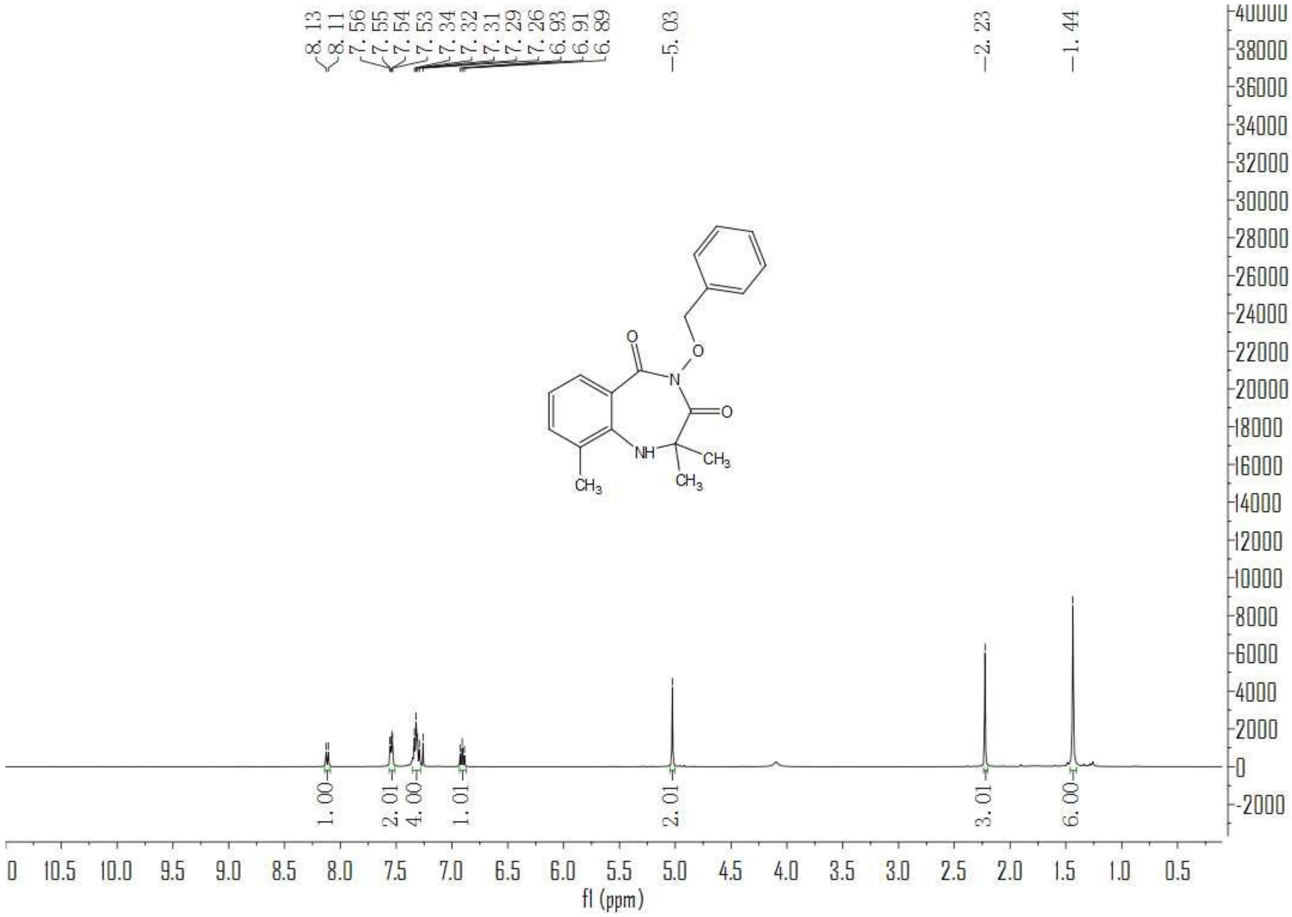

^{1}H NMR spectra (400 MHz, $CDCl_3$) of 4l

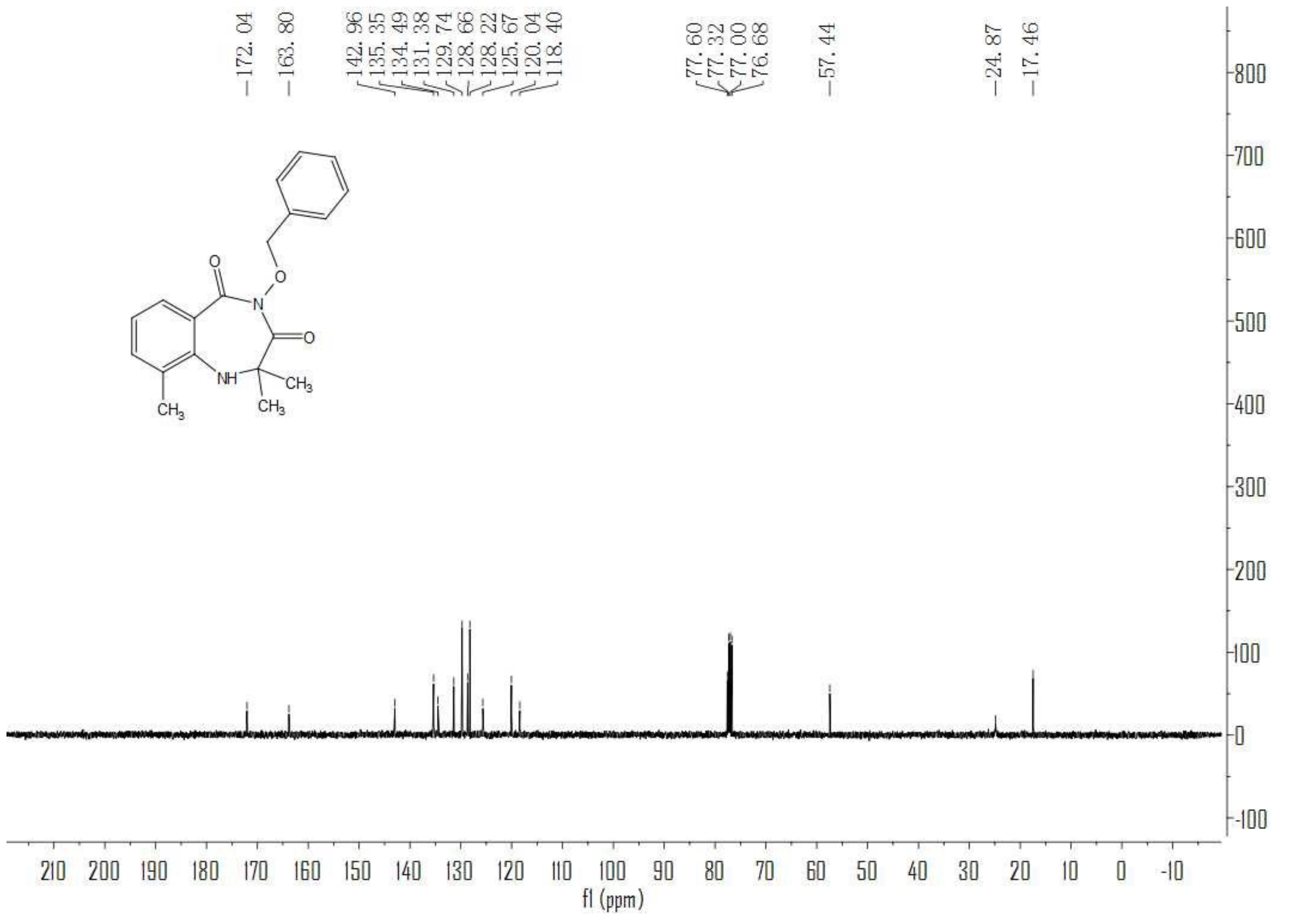

^{13}C NMR spectra (100 MHz, $CDCl_3$) of 4l

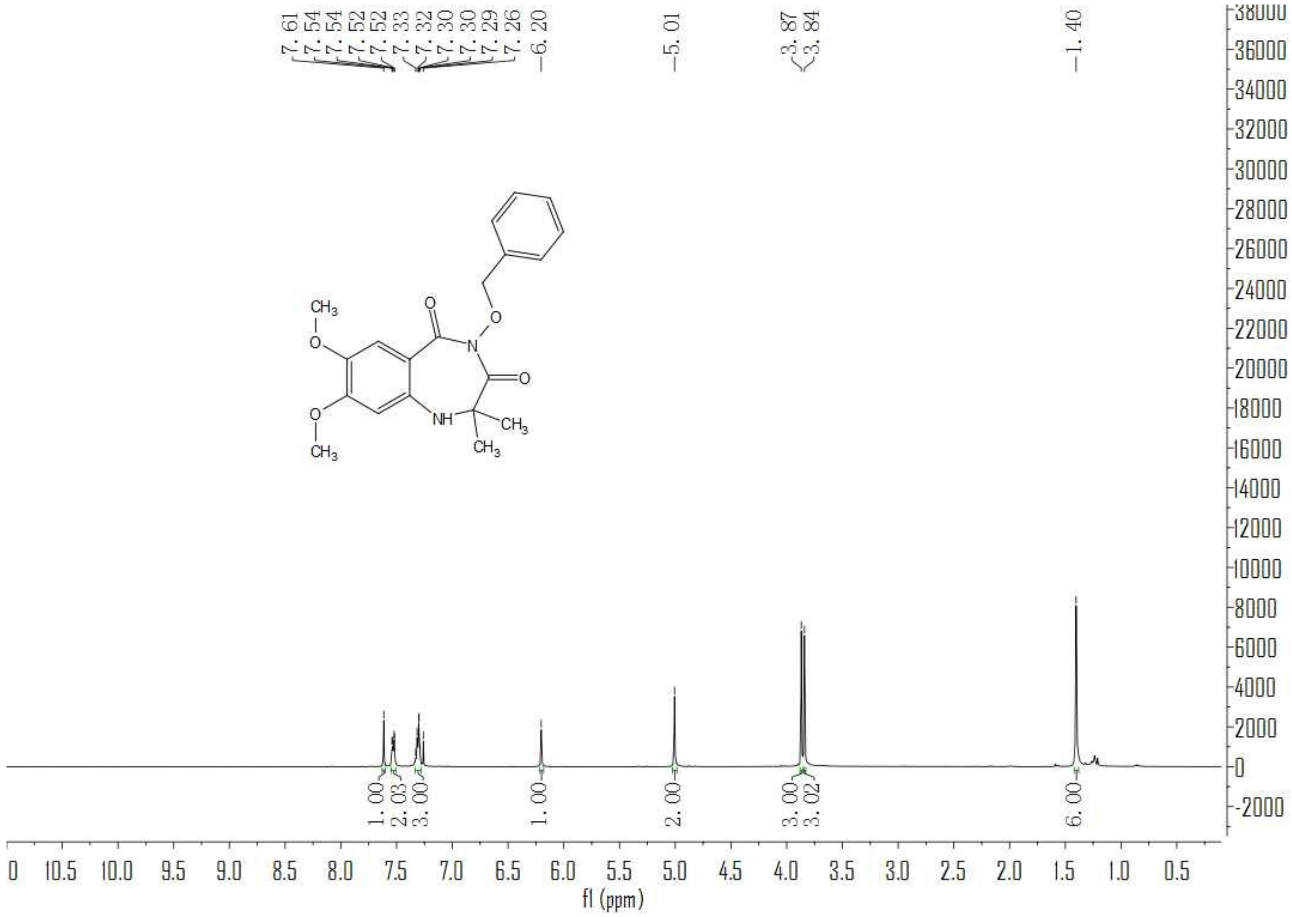

^1H NMR spectra (400 MHz, $CDCl_3$) of 4m

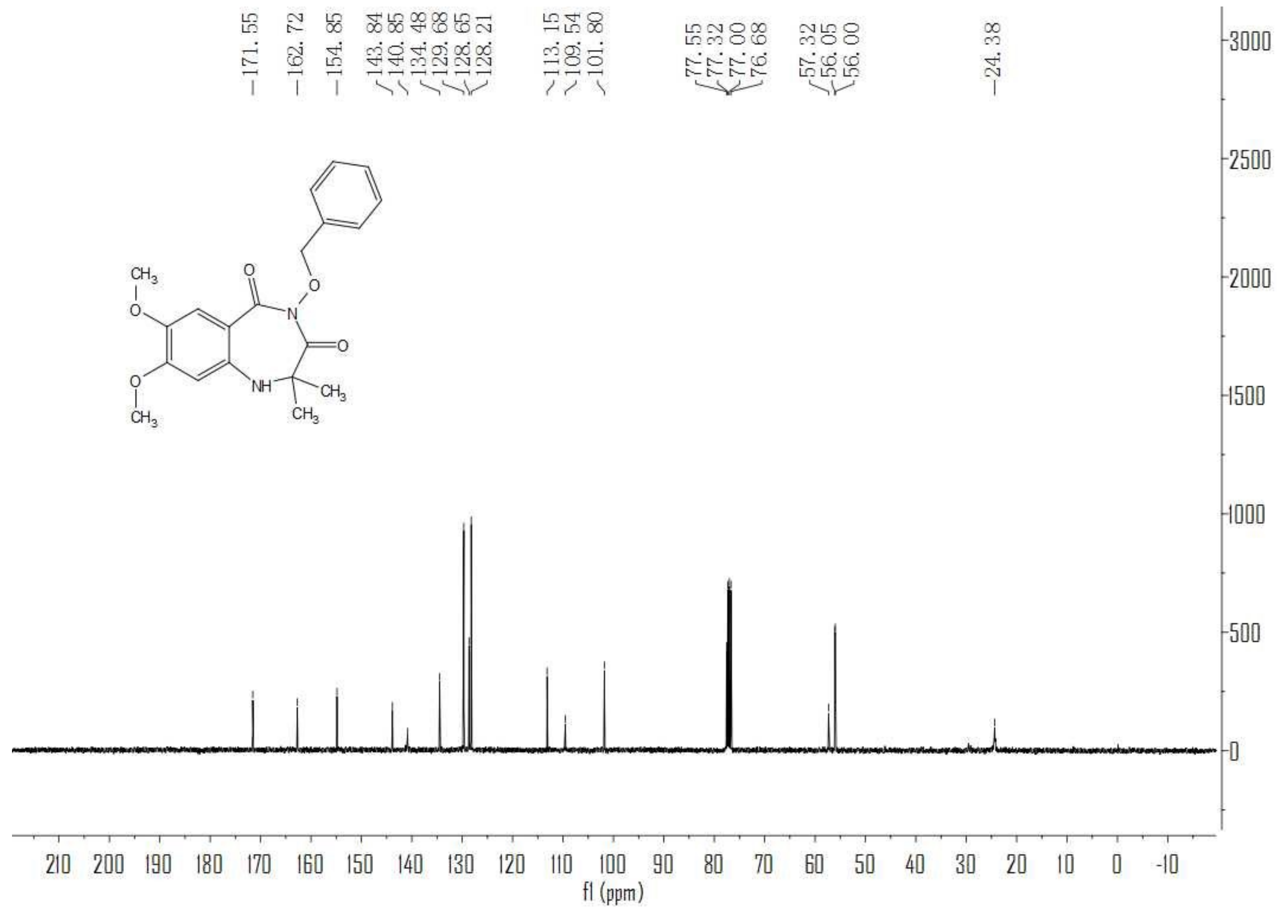

^{13}C NMR spectra (100 MHz, $CDCl_3$) of 4m

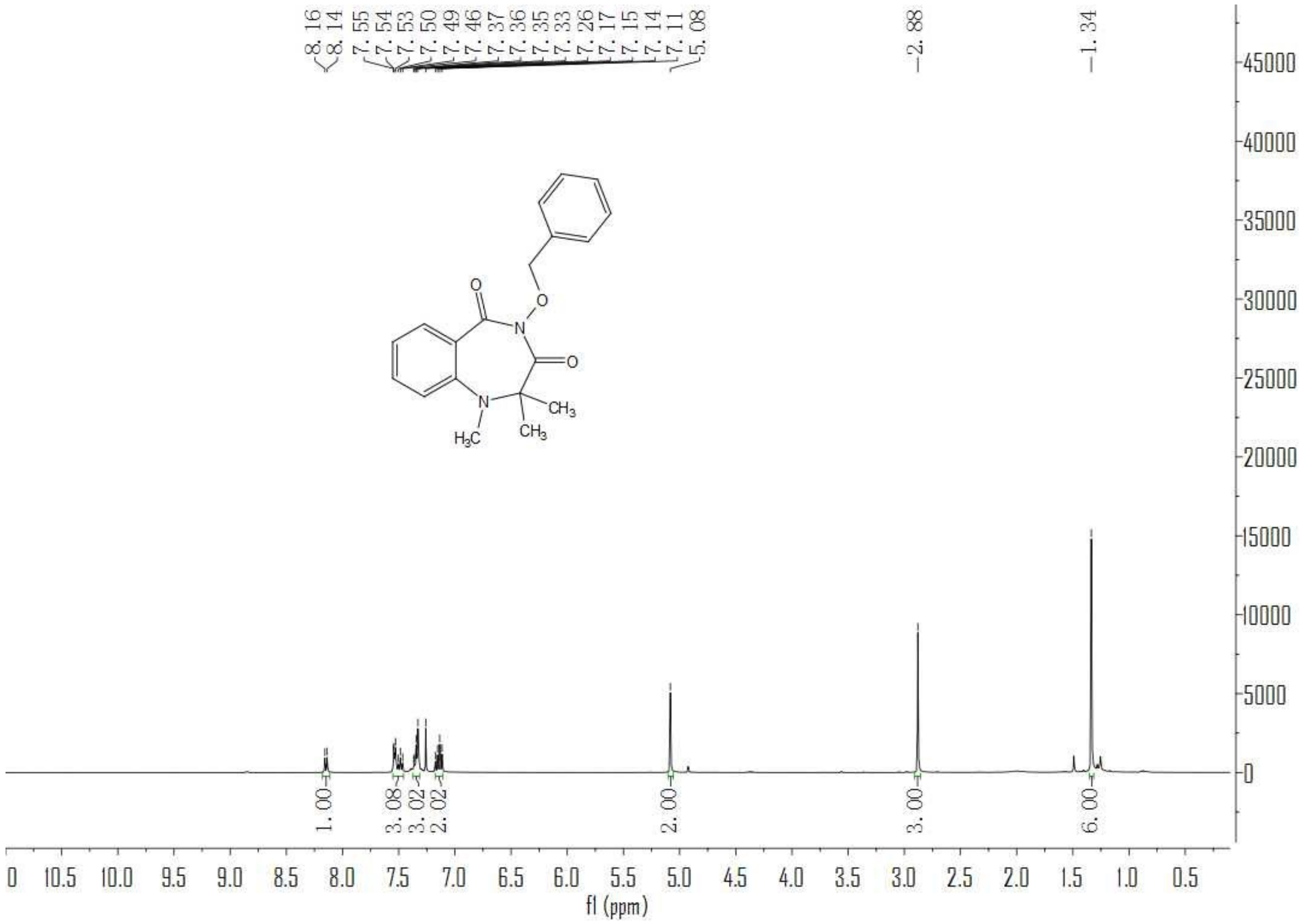

^{1}H NMR spectra (400 MHz, $CDCl_3$) of 4n

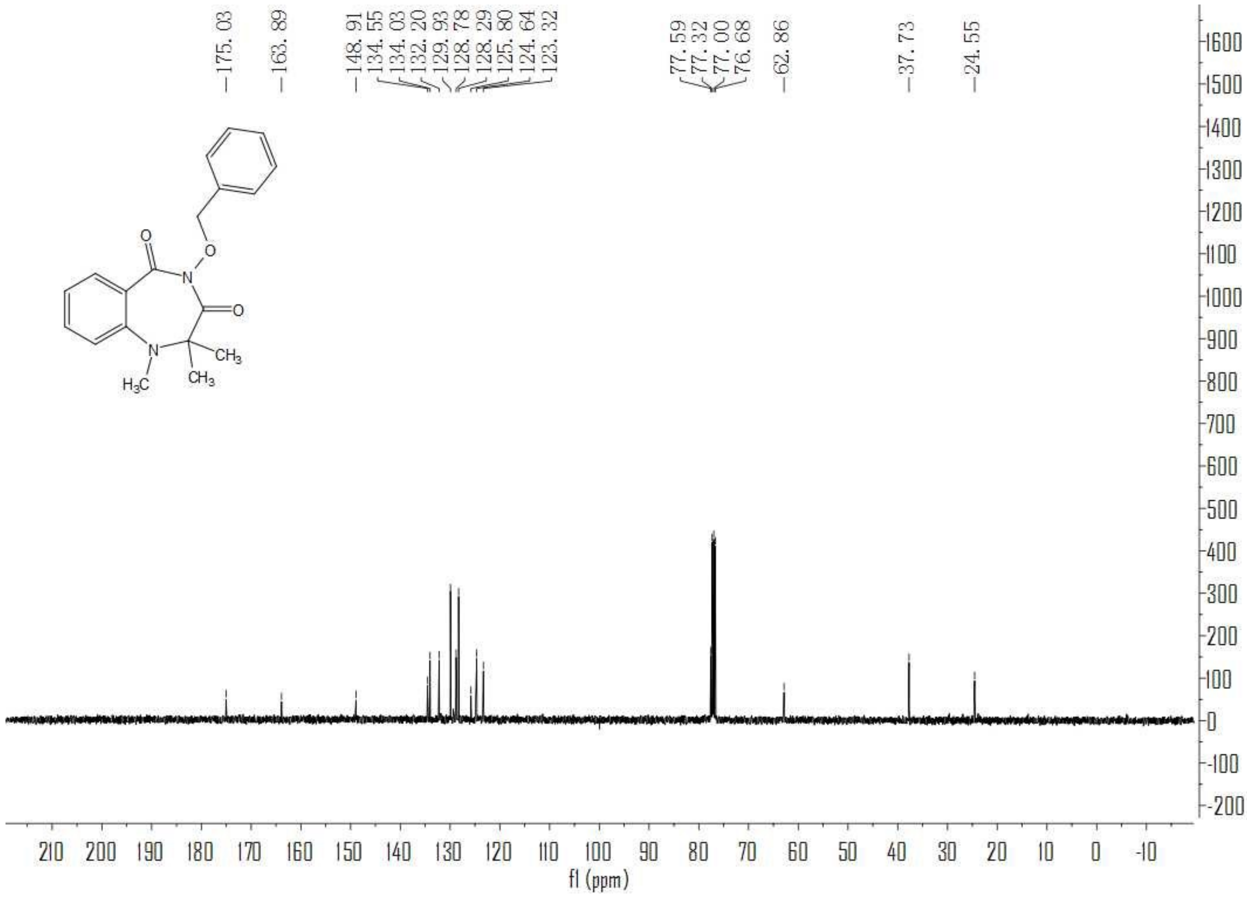

^{13}C NMR spectra (100 MHz, $CDCl_3$) of 4n

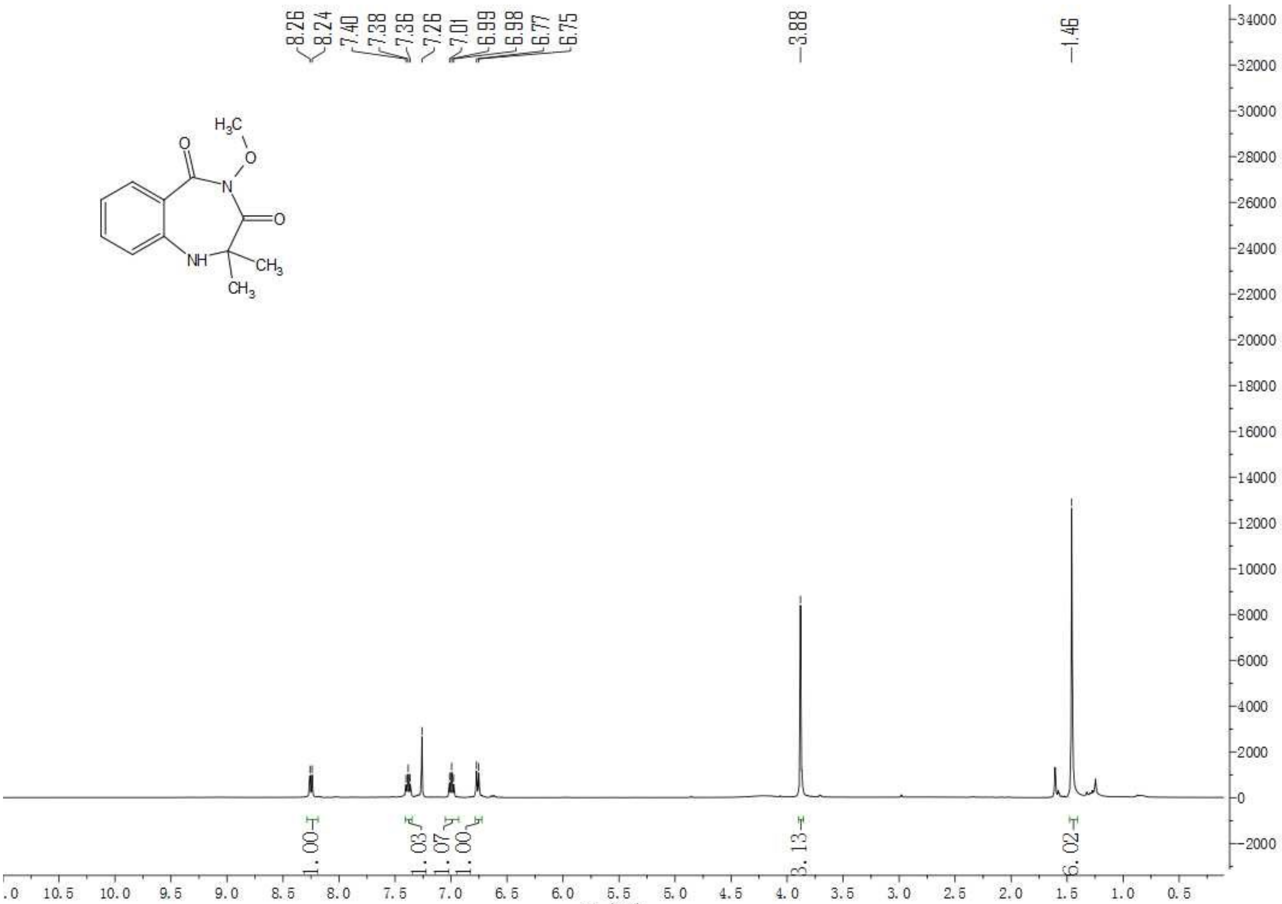

^{1}H NMR spectra (100 MHz, $CDCl_3$) of 4o

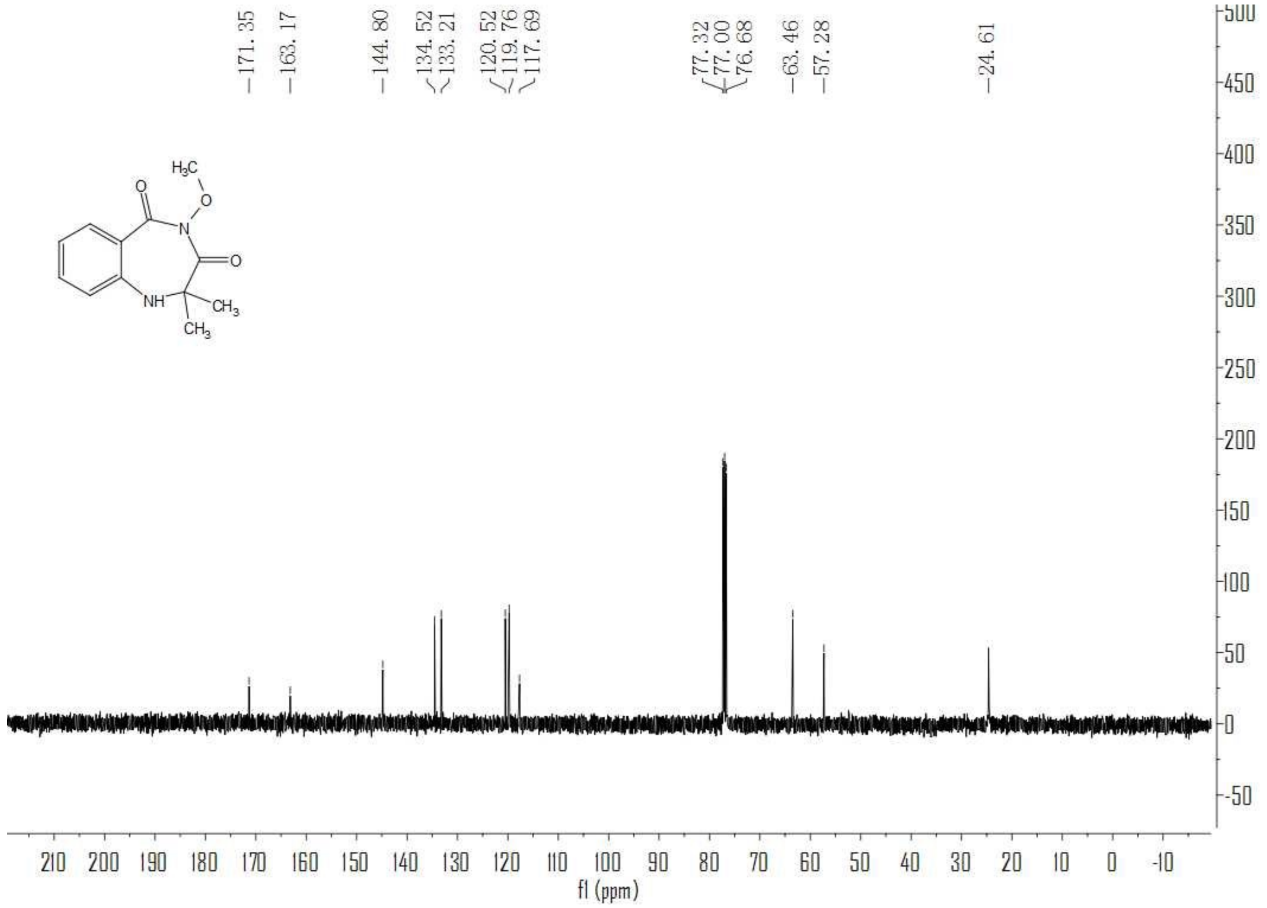

^{13}C NMR spectra (100 MHz, $CDCl_3$) of 4o

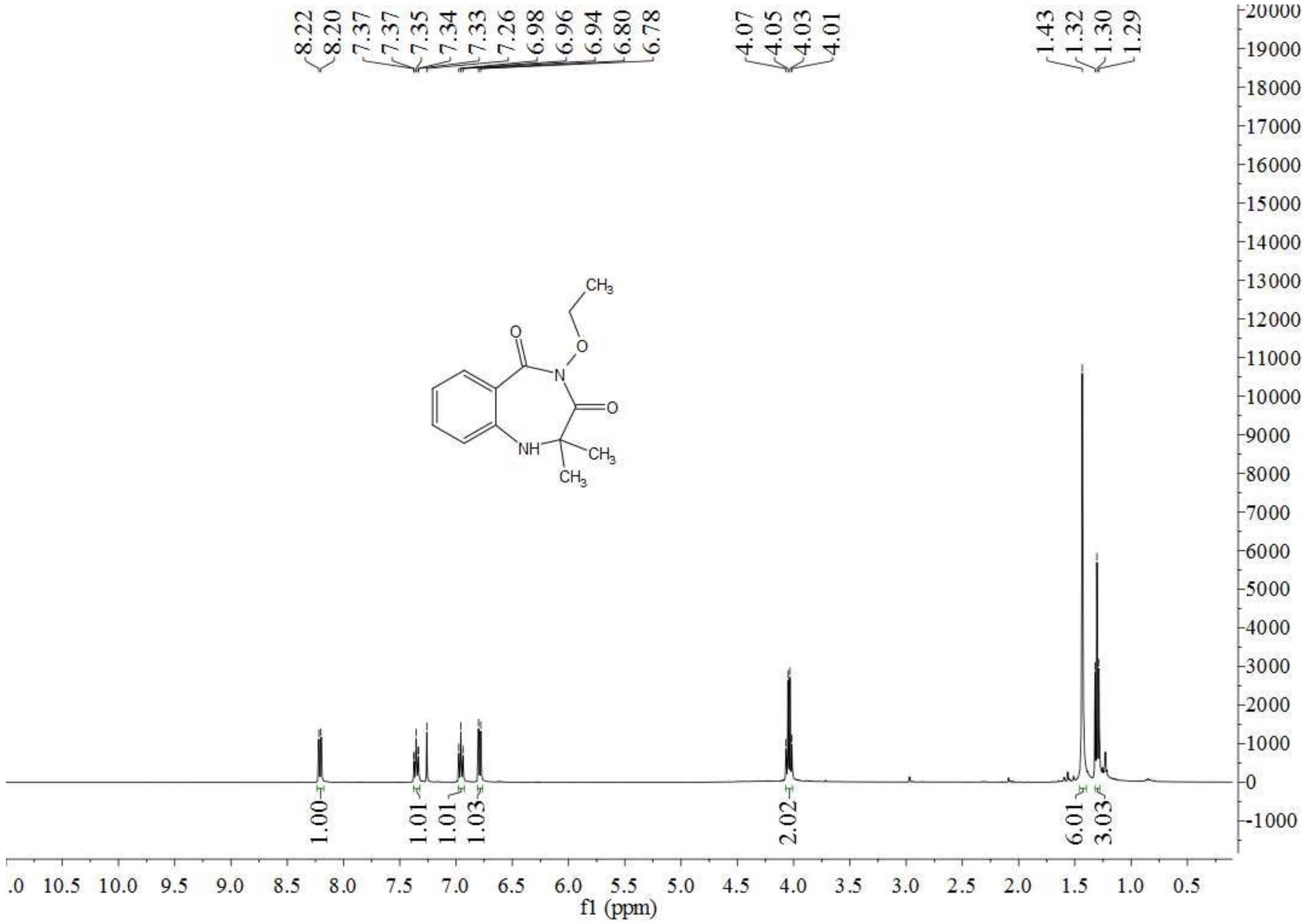

^{1}H NMR spectra (400 MHz, CDCl$_3$) of 4p

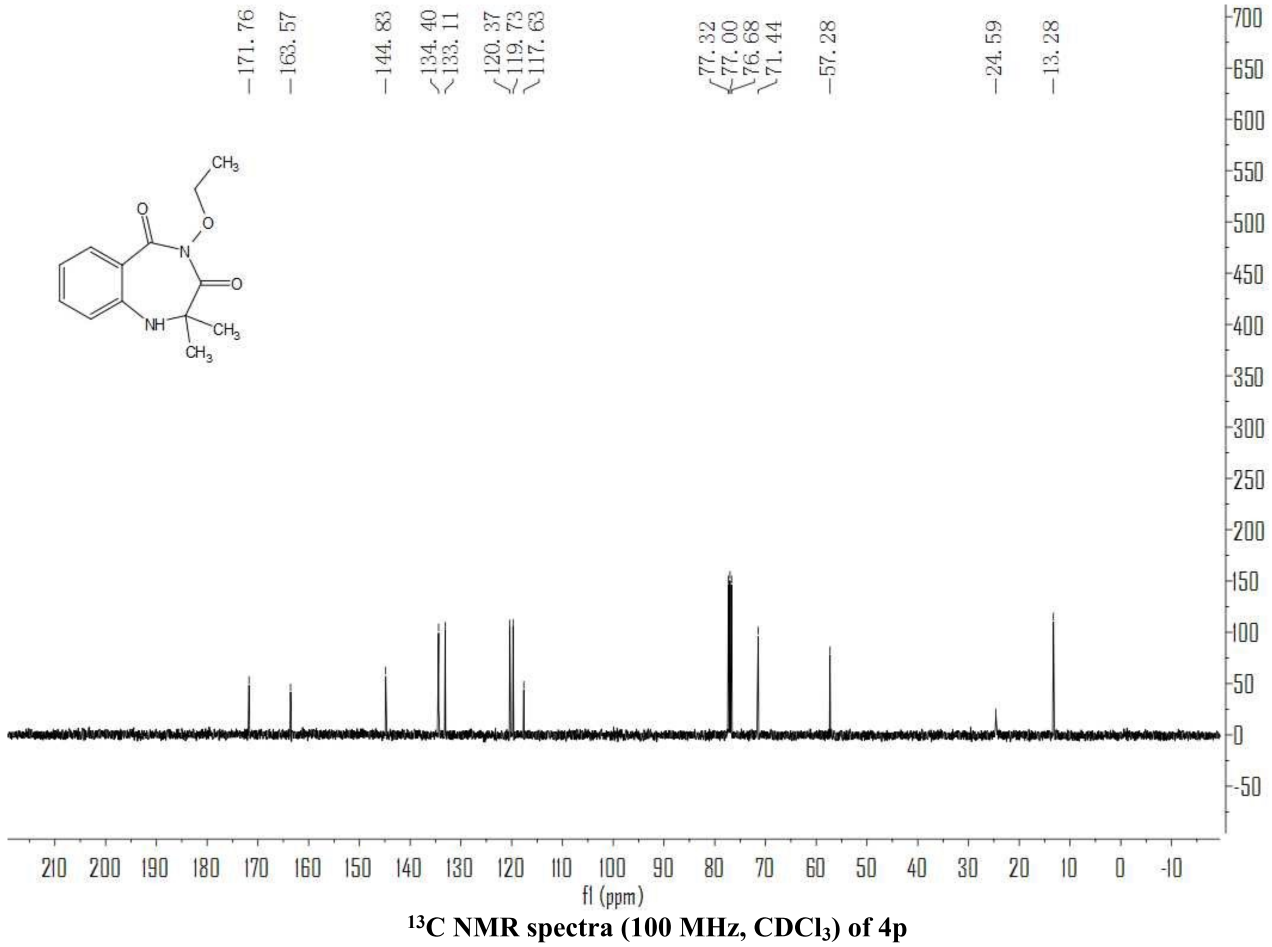

^{13}C NMR spectra (100 MHz, CDCl$_3$) of 4p

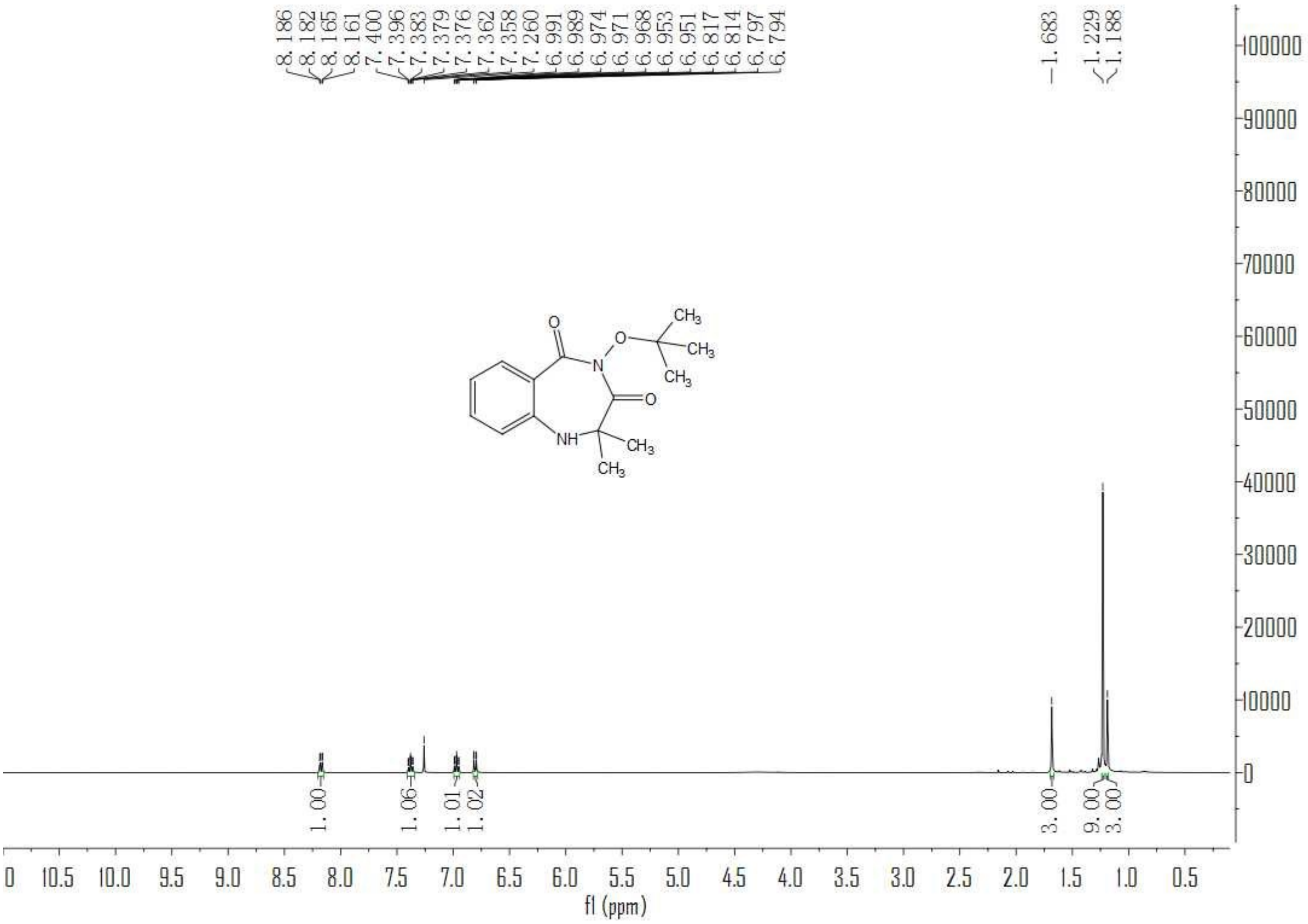

^{1}H NMR spectra (400 MHz, $CDCl_3$) of 4q

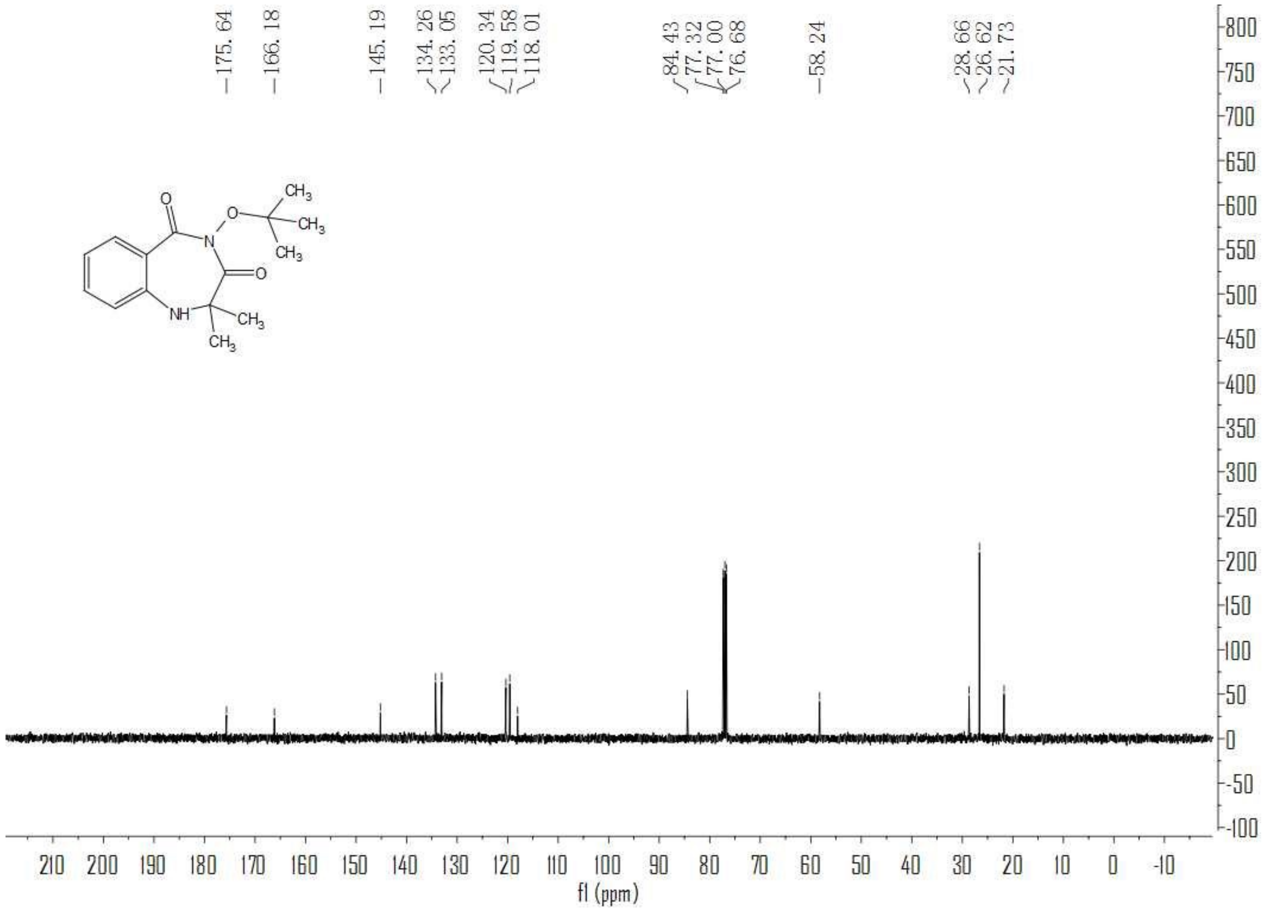

^{13}C NMR spectra (100 MHz, $CDCl_3$) of 4q

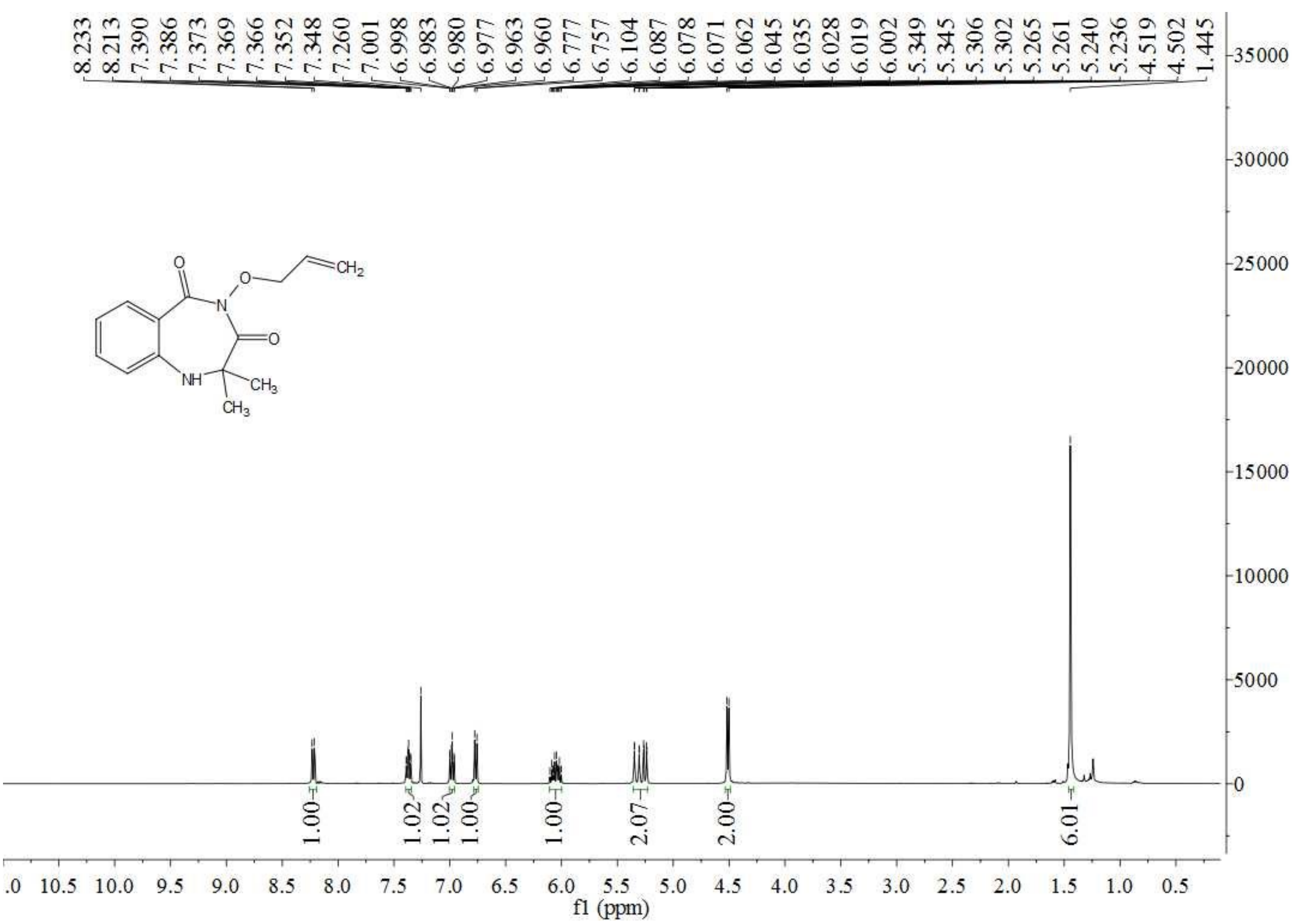

^{1}H NMR spectra (400 MHz, CDCl$_3$) of 4r

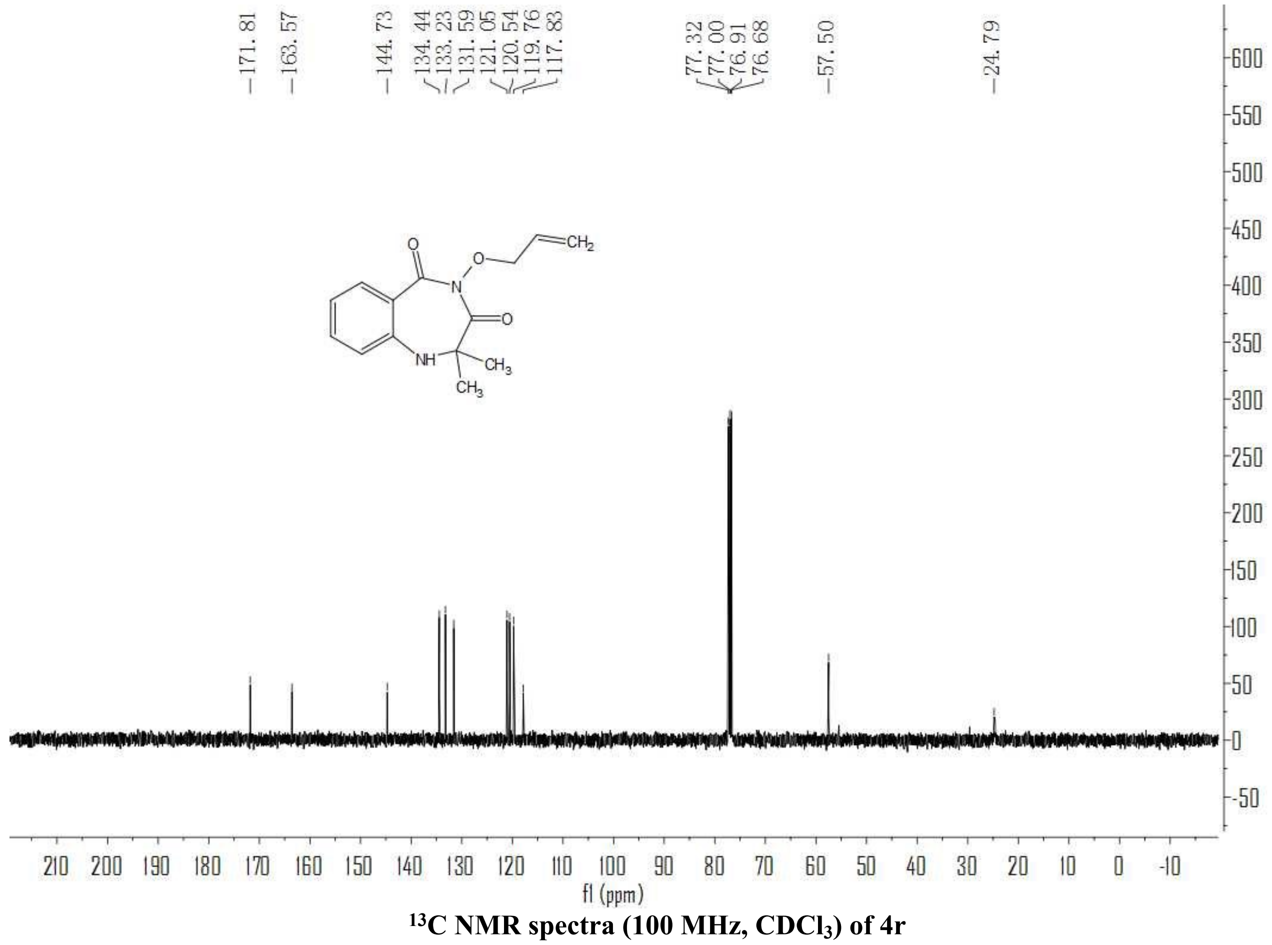

^{13}C NMR spectra (100 MHz, CDCl$_3$) of 4r

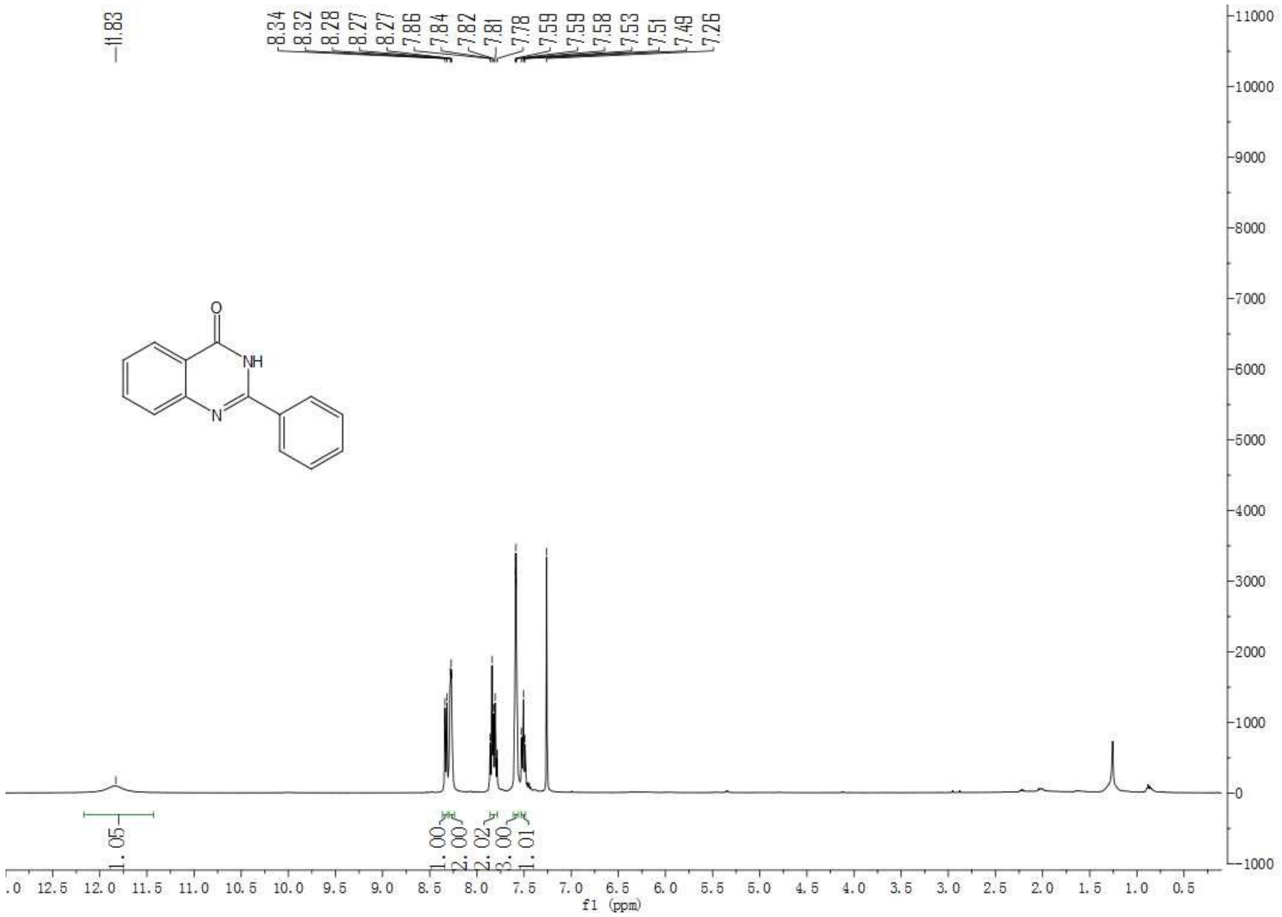

^{1}H NMR spectra (400 MHz, $CDCl_3$) of 6a

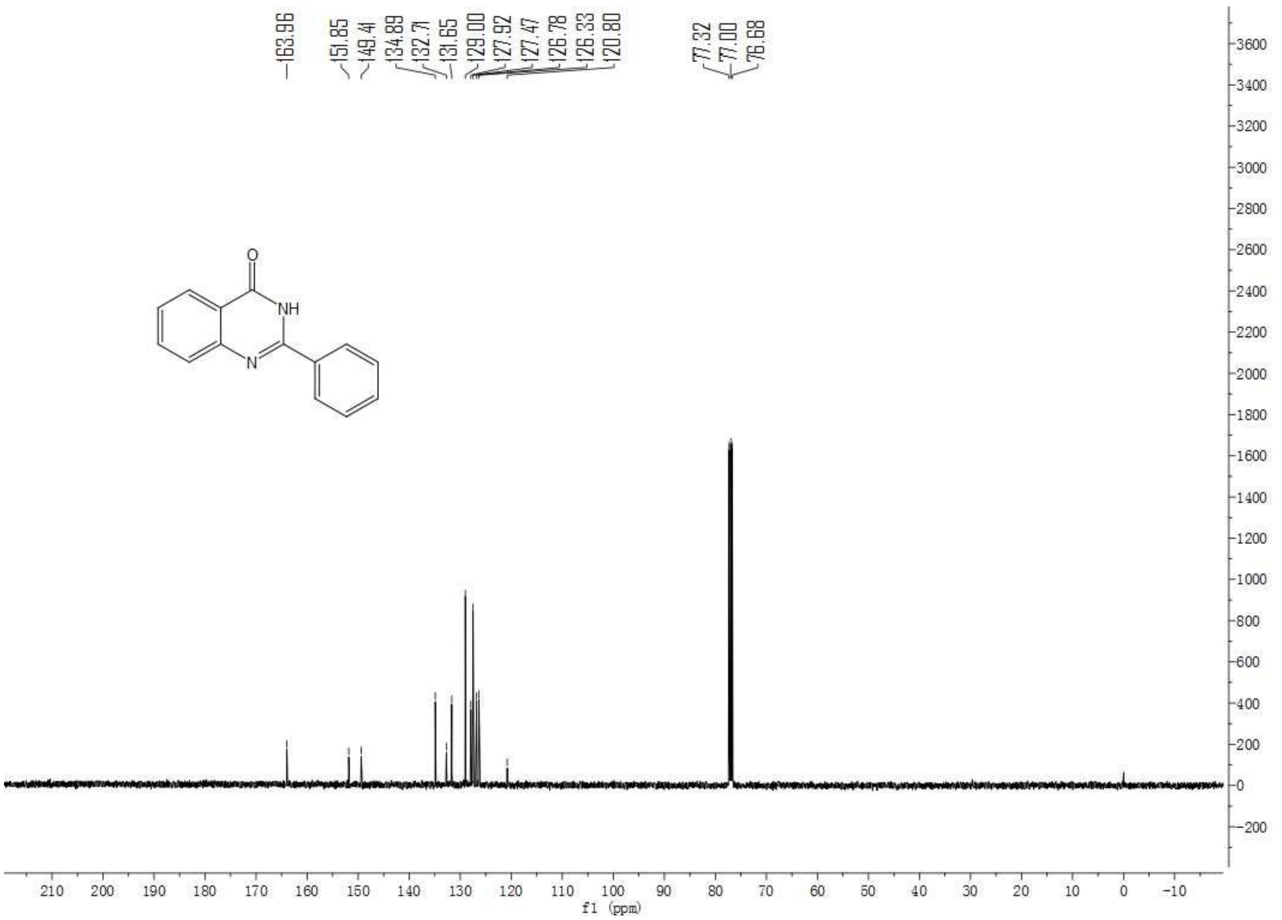

^{13}C NMR spectra (100 MHz, $CDCl_3$) of 6a

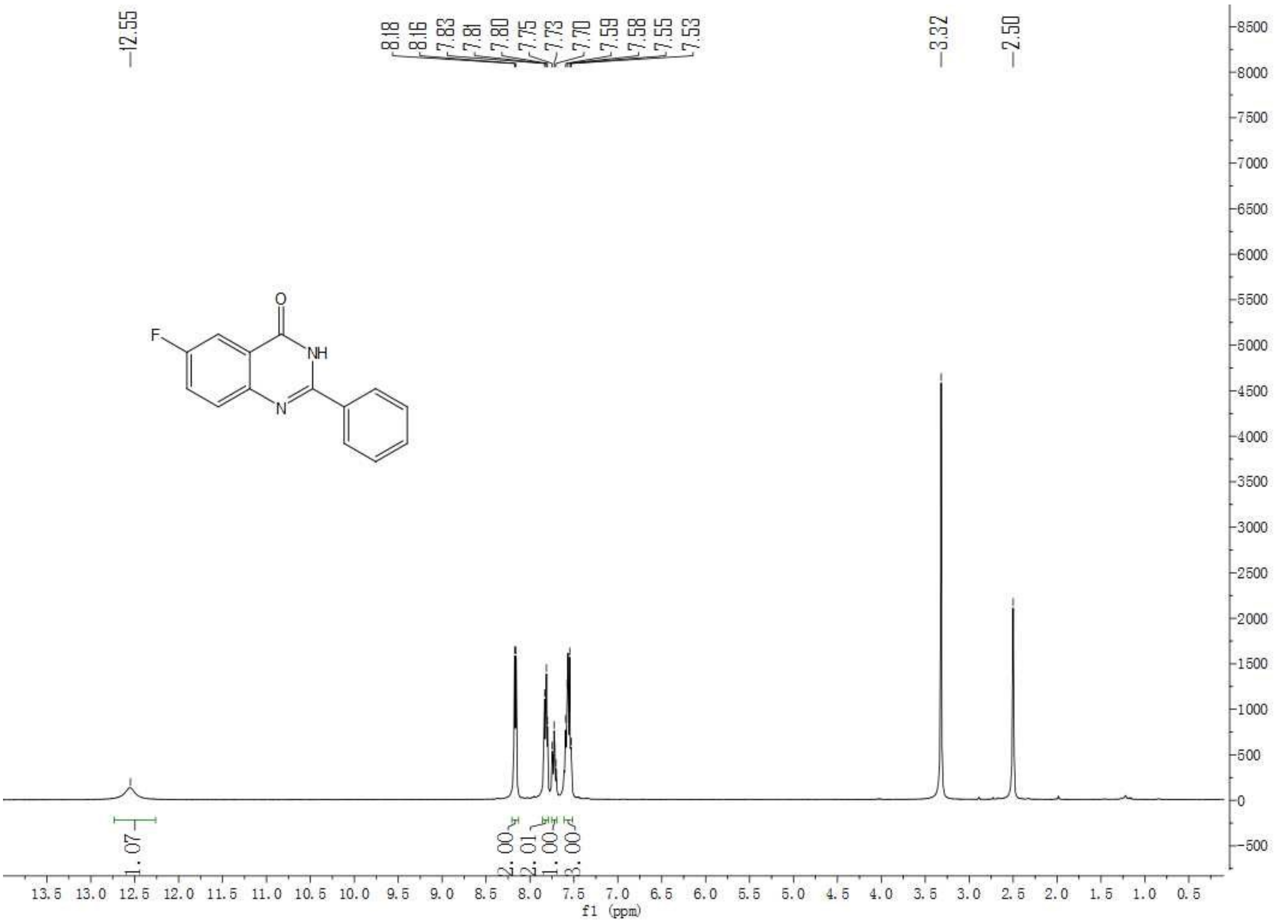

^{1}H NMR spectra (400 MHz, DMSO-d_6) of 6b

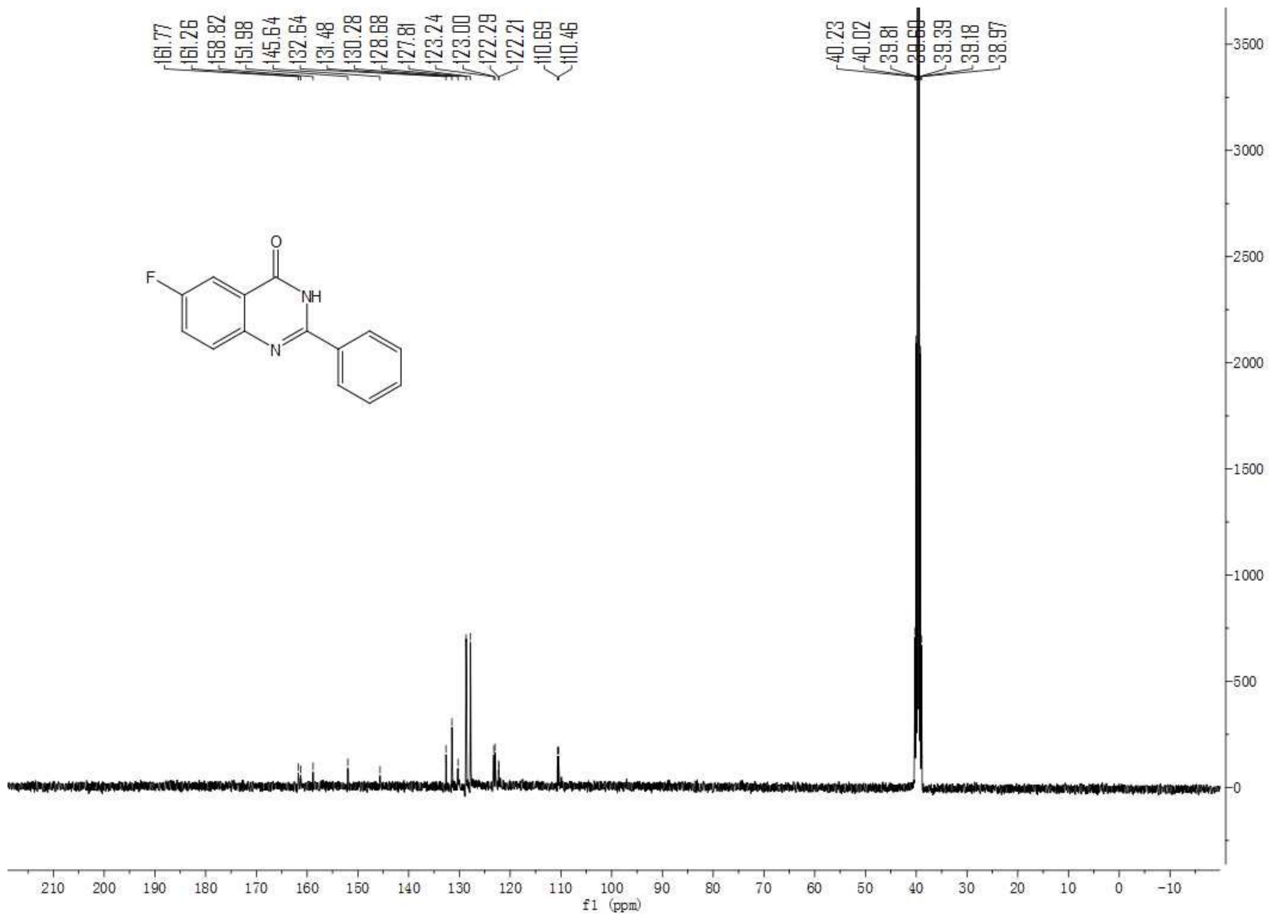

^{13}C NMR spectra (100 MHz, DMSO-d_6) of 6b

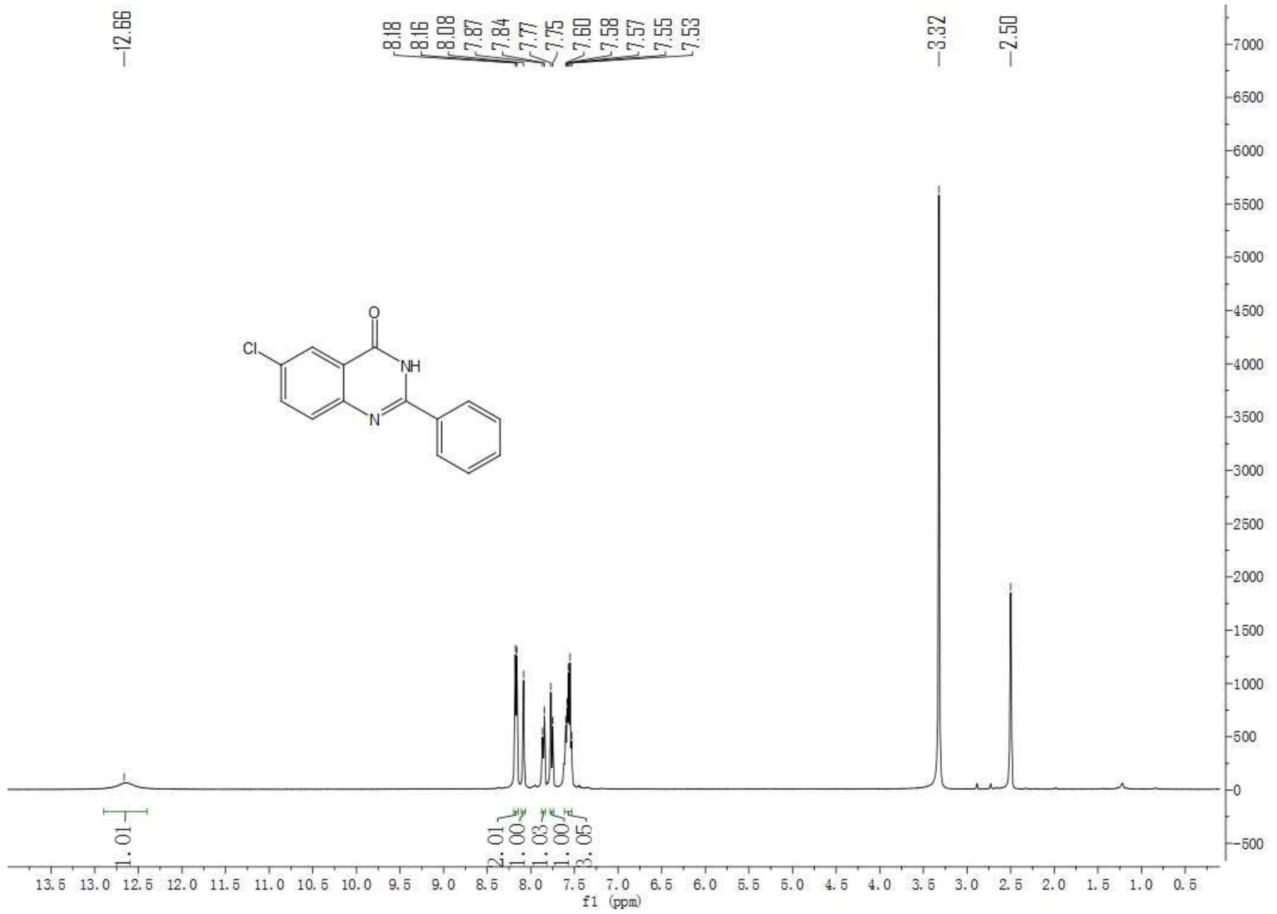

^{1}H NMR spectra (400 MHz, DMSO-d_6**) of 6c**

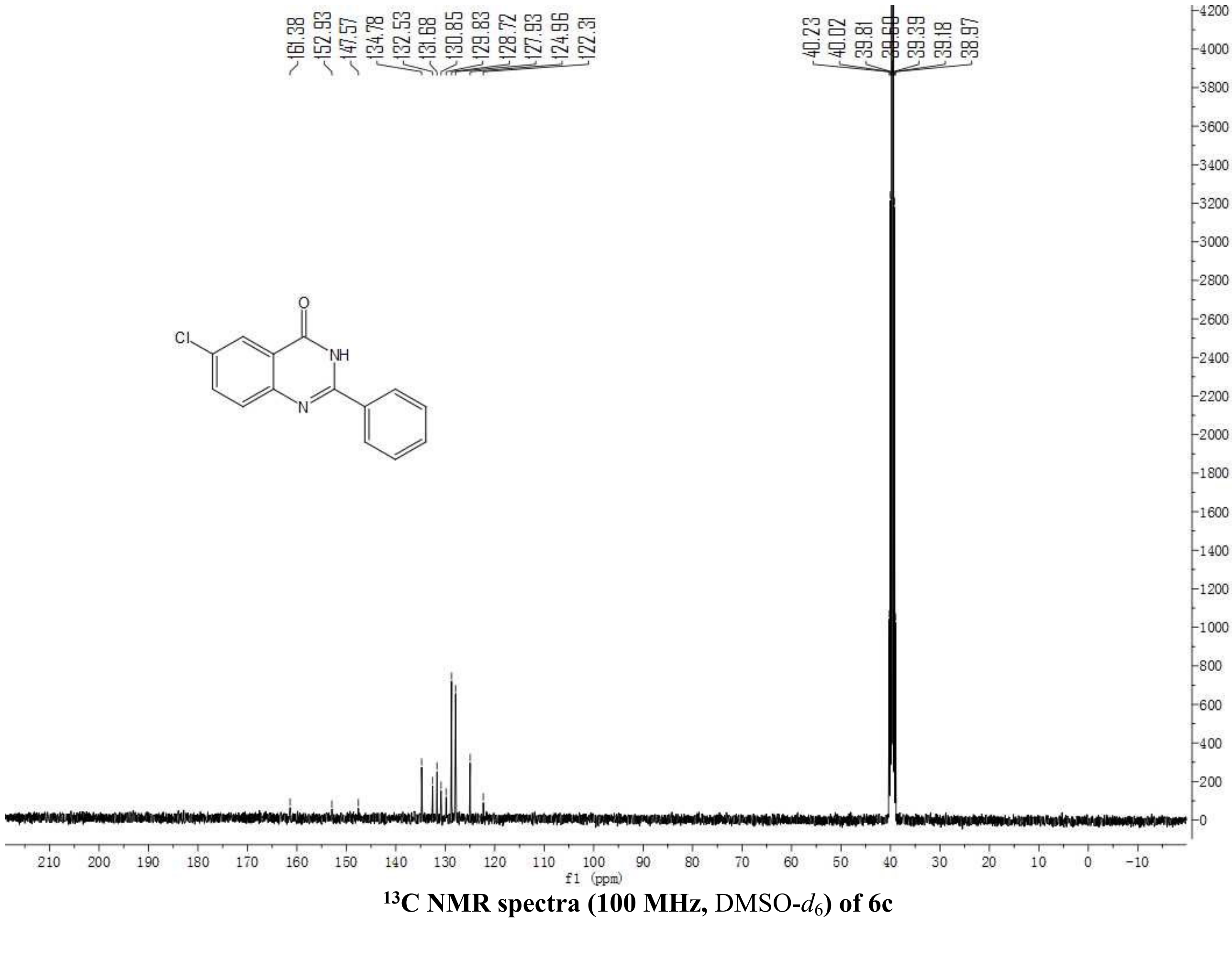

^{13}C NMR spectra (100 MHz, DMSO-d_6**) of 6c**

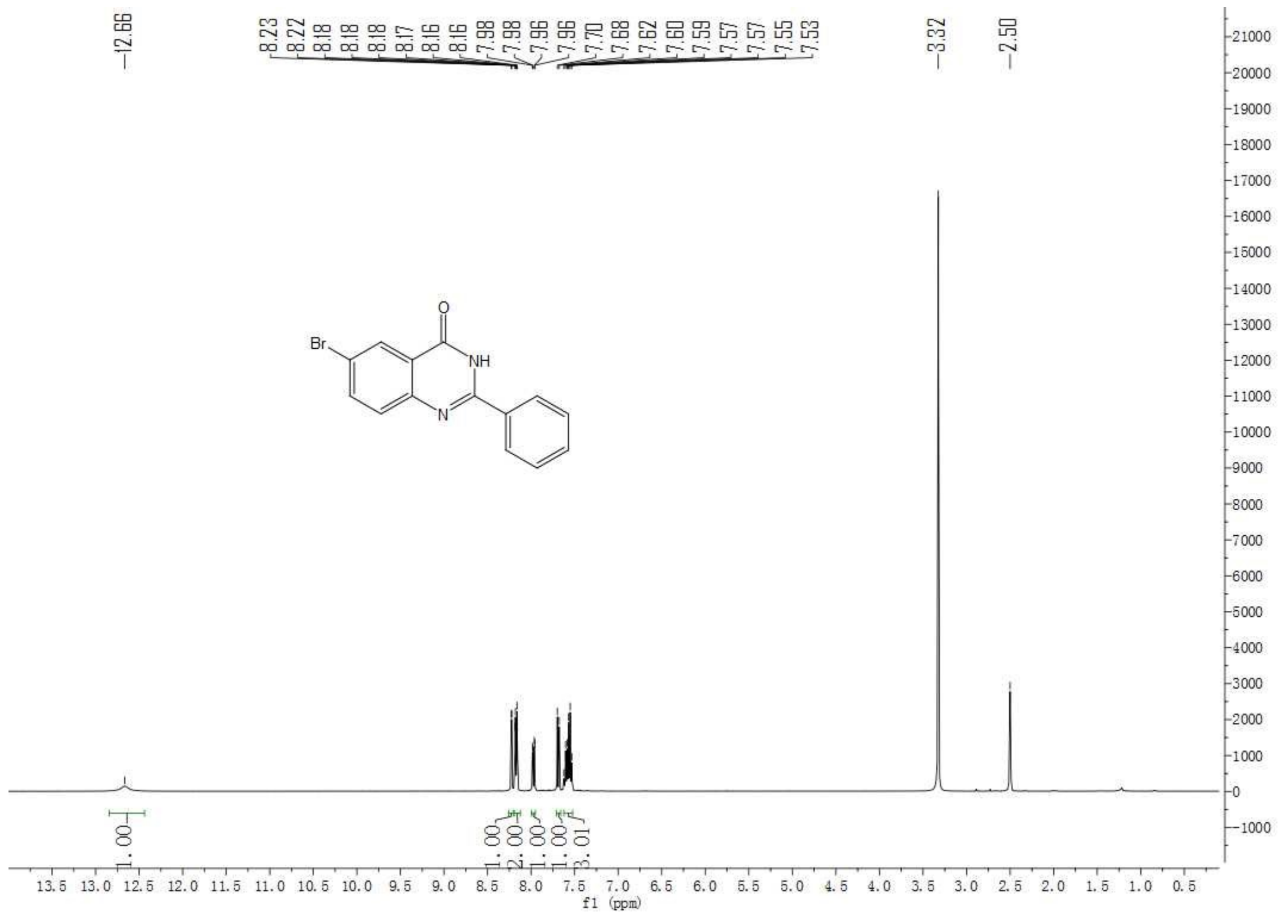

^{1}H NMR spectra (400 MHz, DMSO-d_6**) of 6d**

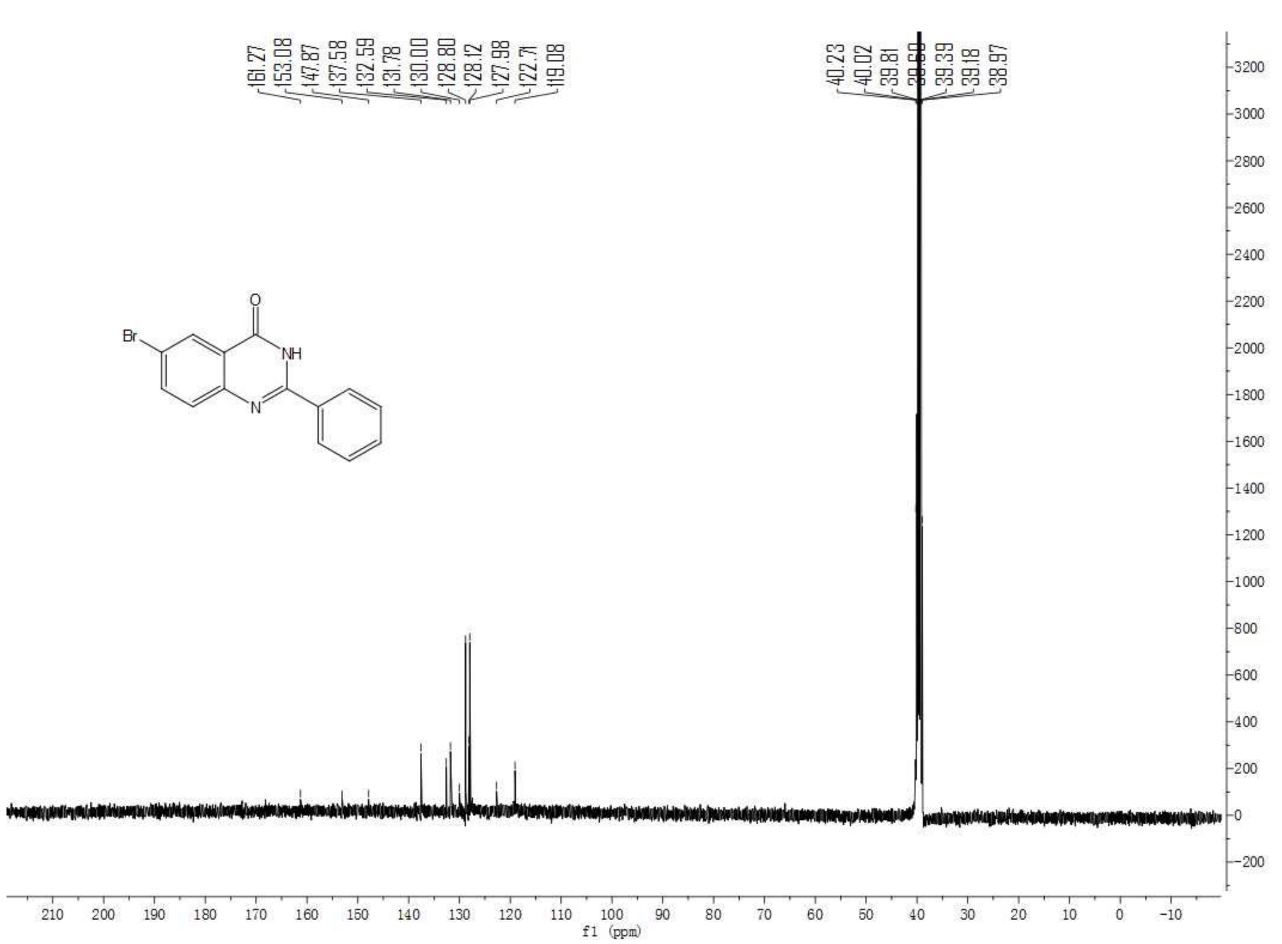

^{13}C NMR spectra (100 MHz, DMSO-d_6**) of 6d**

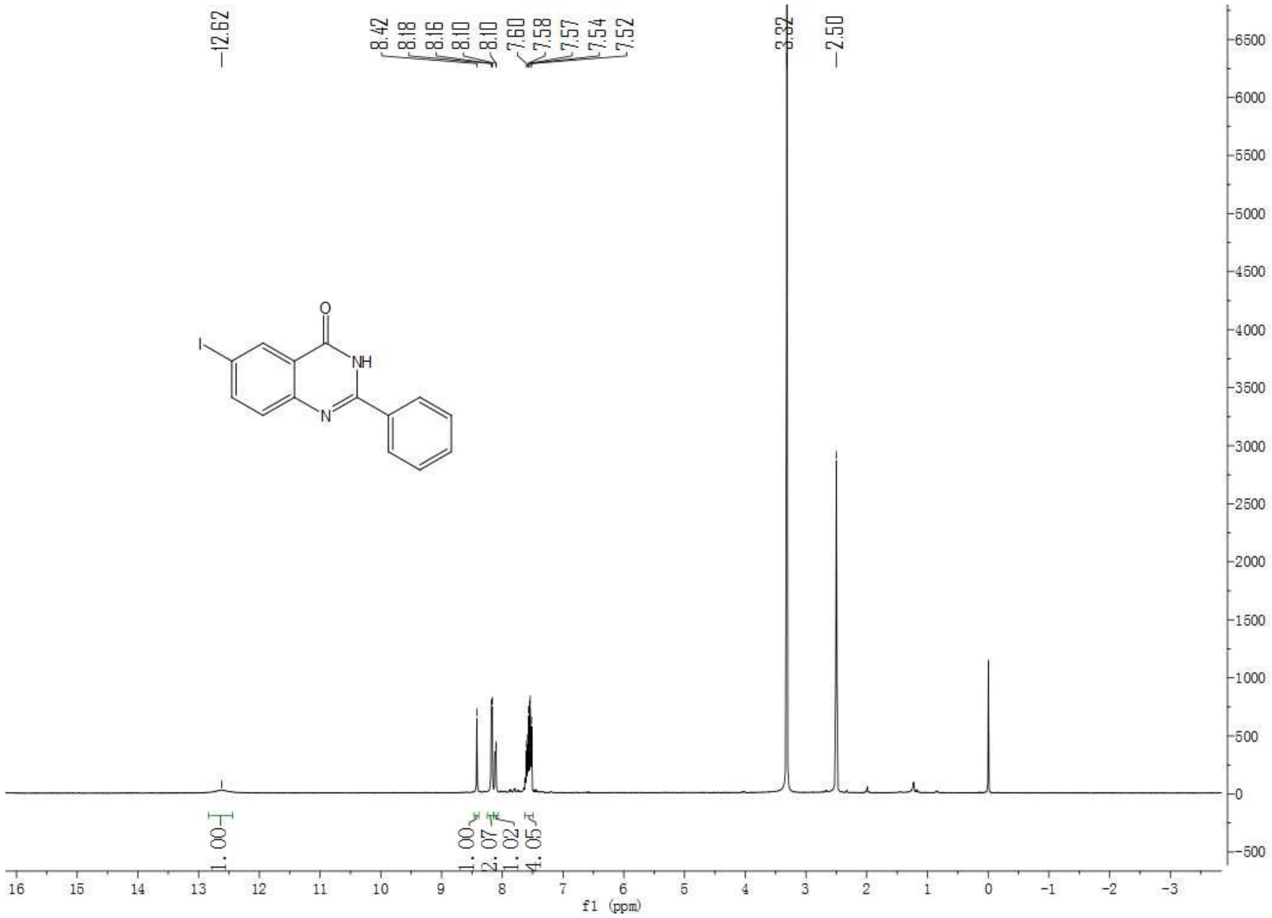

^{1}H NMR spectra (400 MHz, DMSO-d_6) of 6e

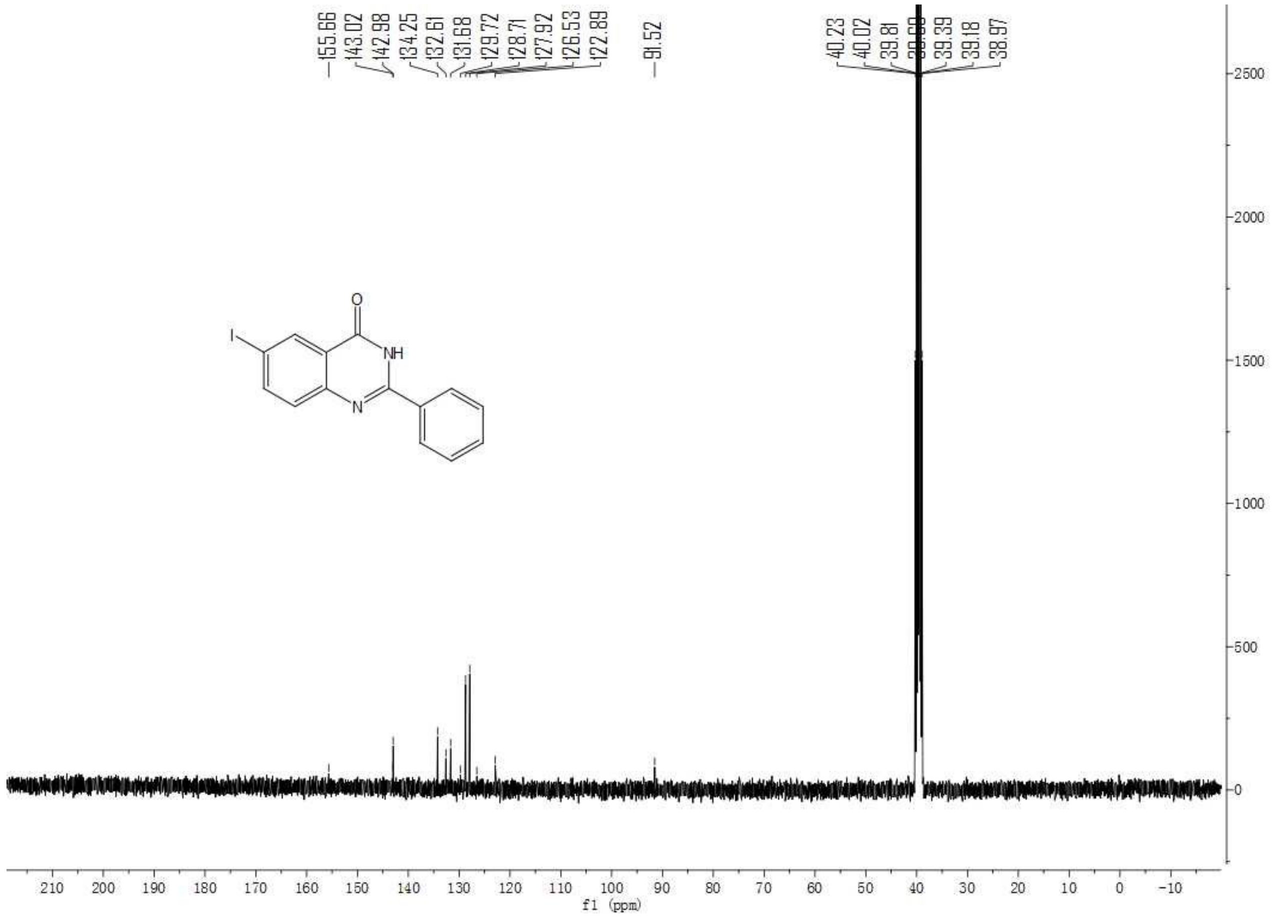

^{13}C NMR spectra (100 MHz, DMSO-d_6) of 6e

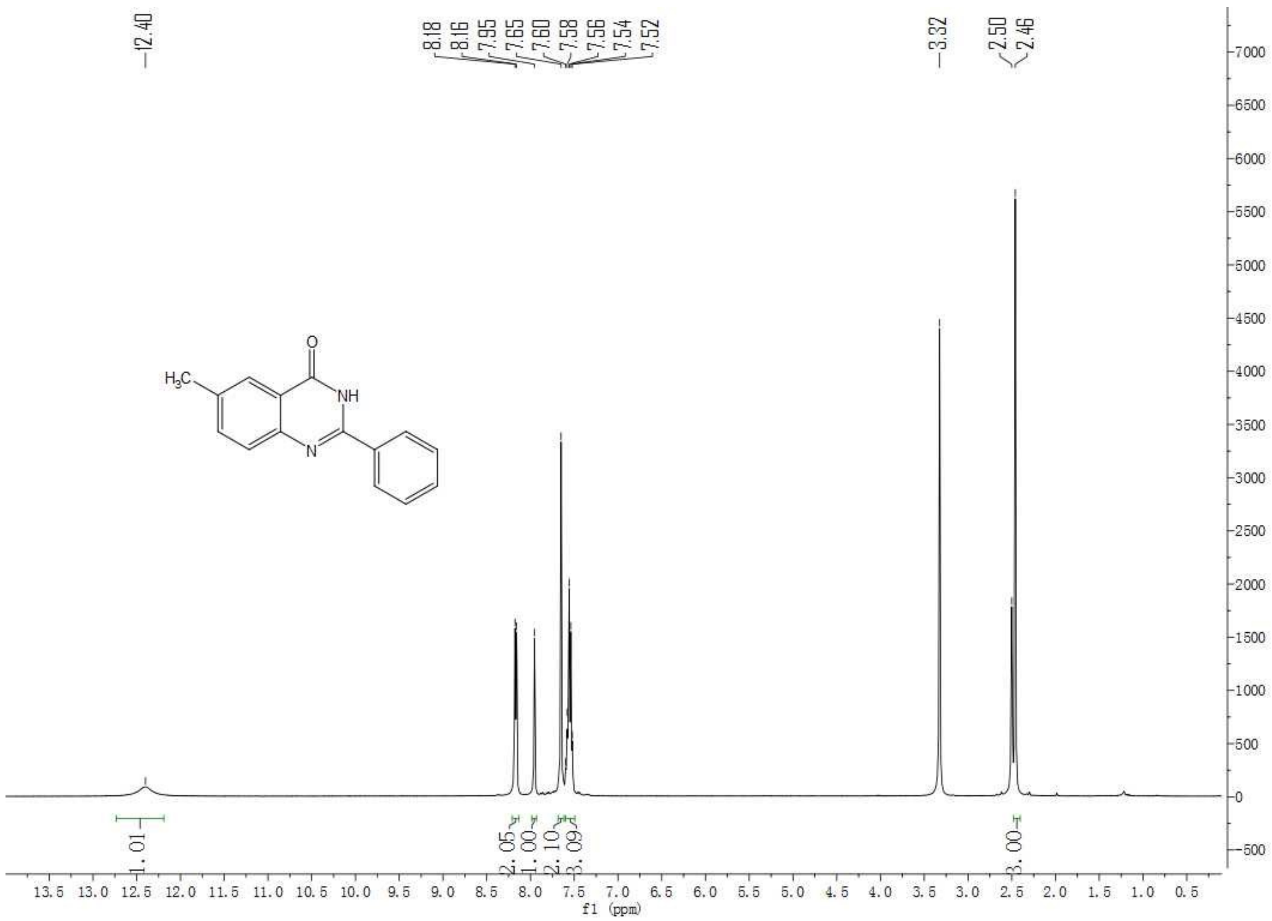

^{1}H NMR spectra (400 MHz, DMSO-d_6) **of 6f**

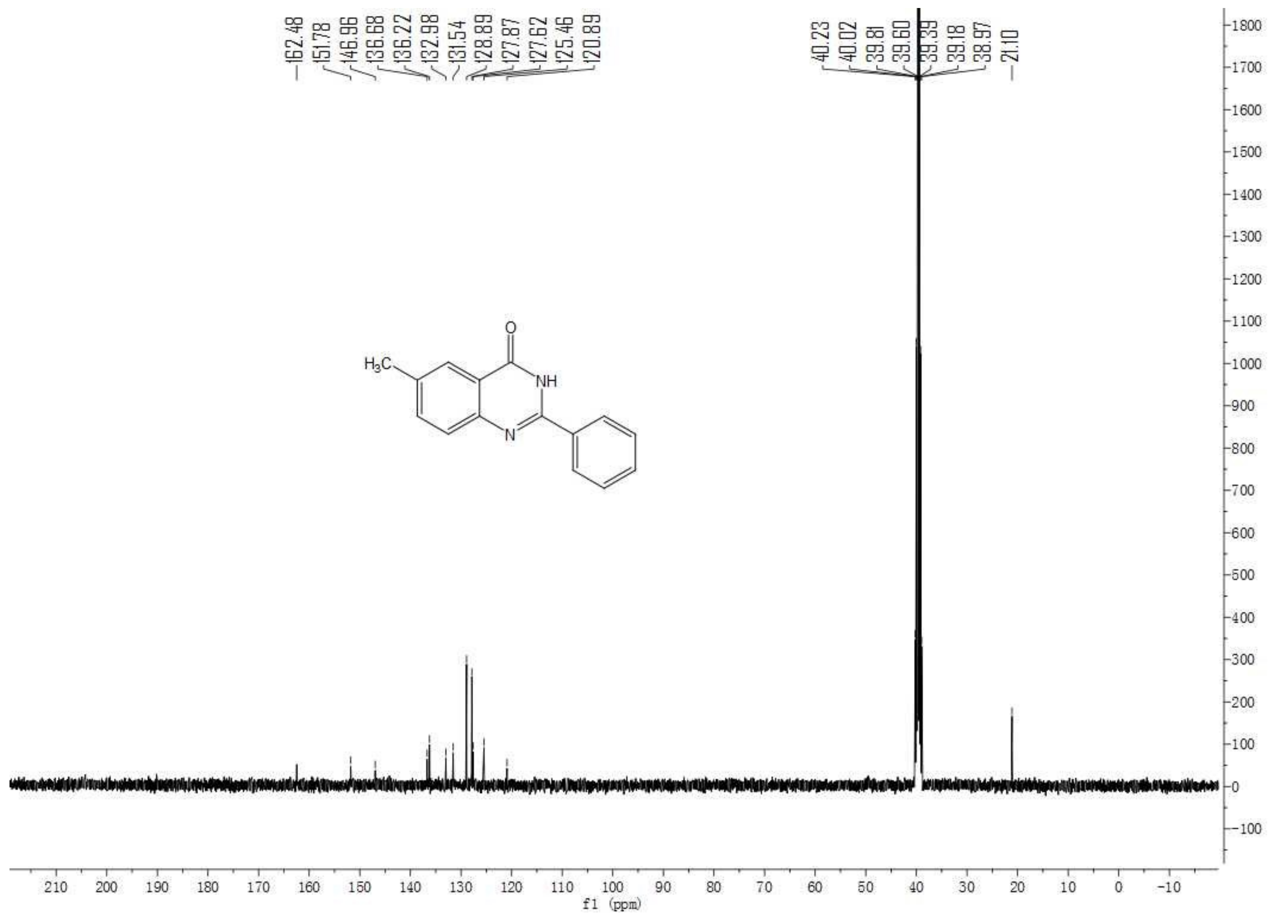

^{13}C NMR spectra (100 MHz, DMSO-d_6) **of 6f**

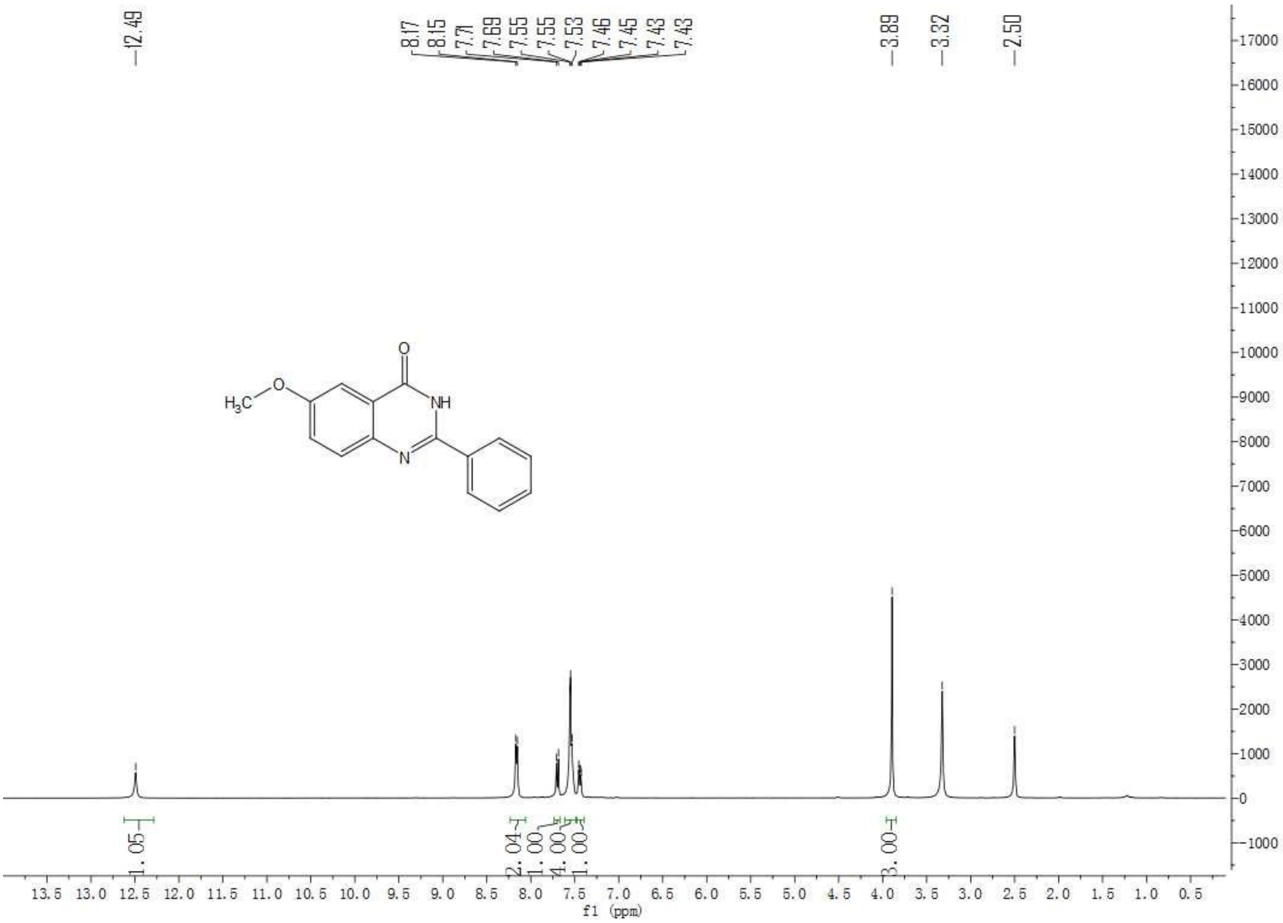

^{1}H NMR spectra (400 MHz, DMSO-d_6) **of 6g**

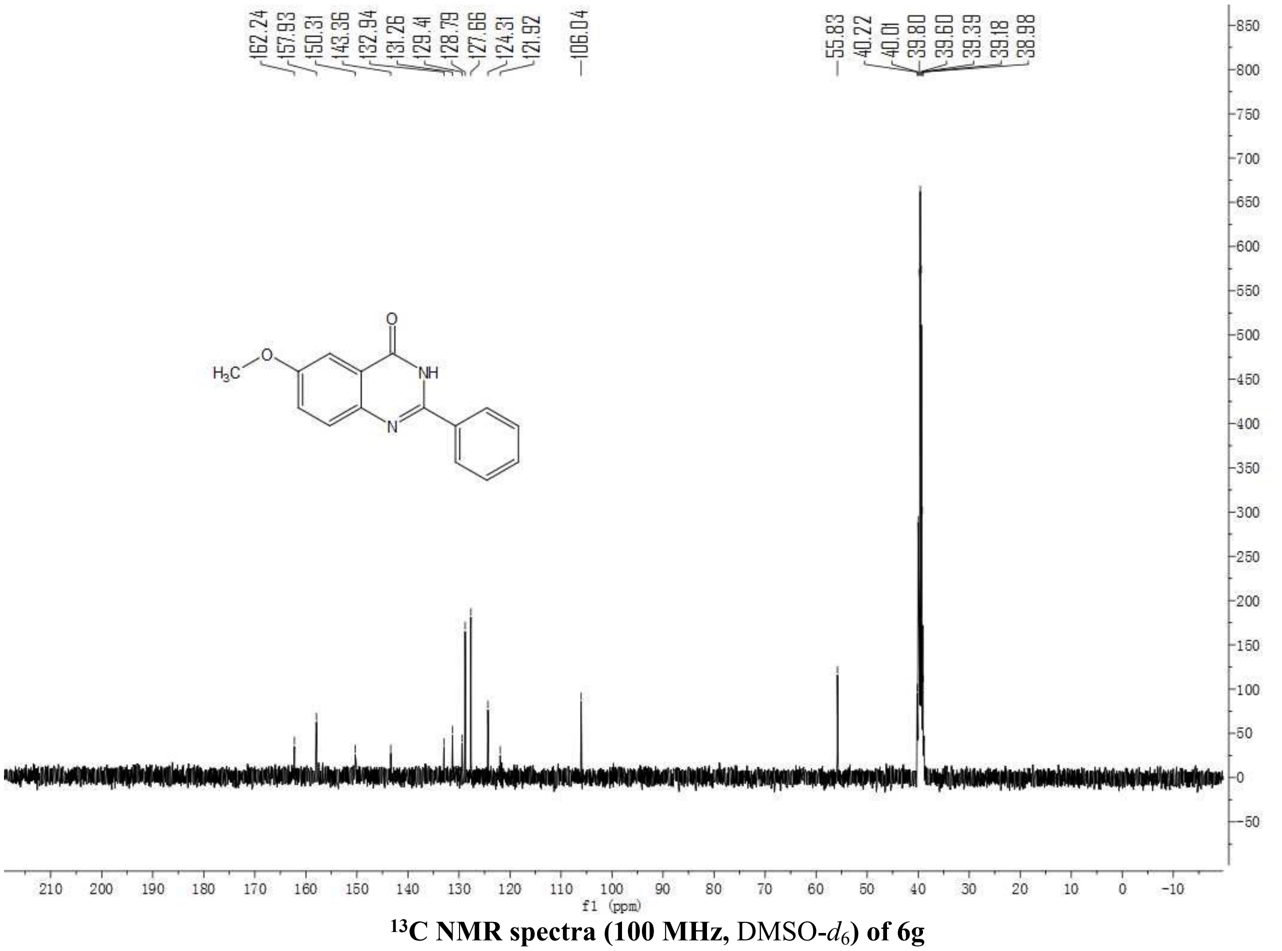

^{13}C NMR spectra (100 MHz, DMSO-d_6) **of 6g**

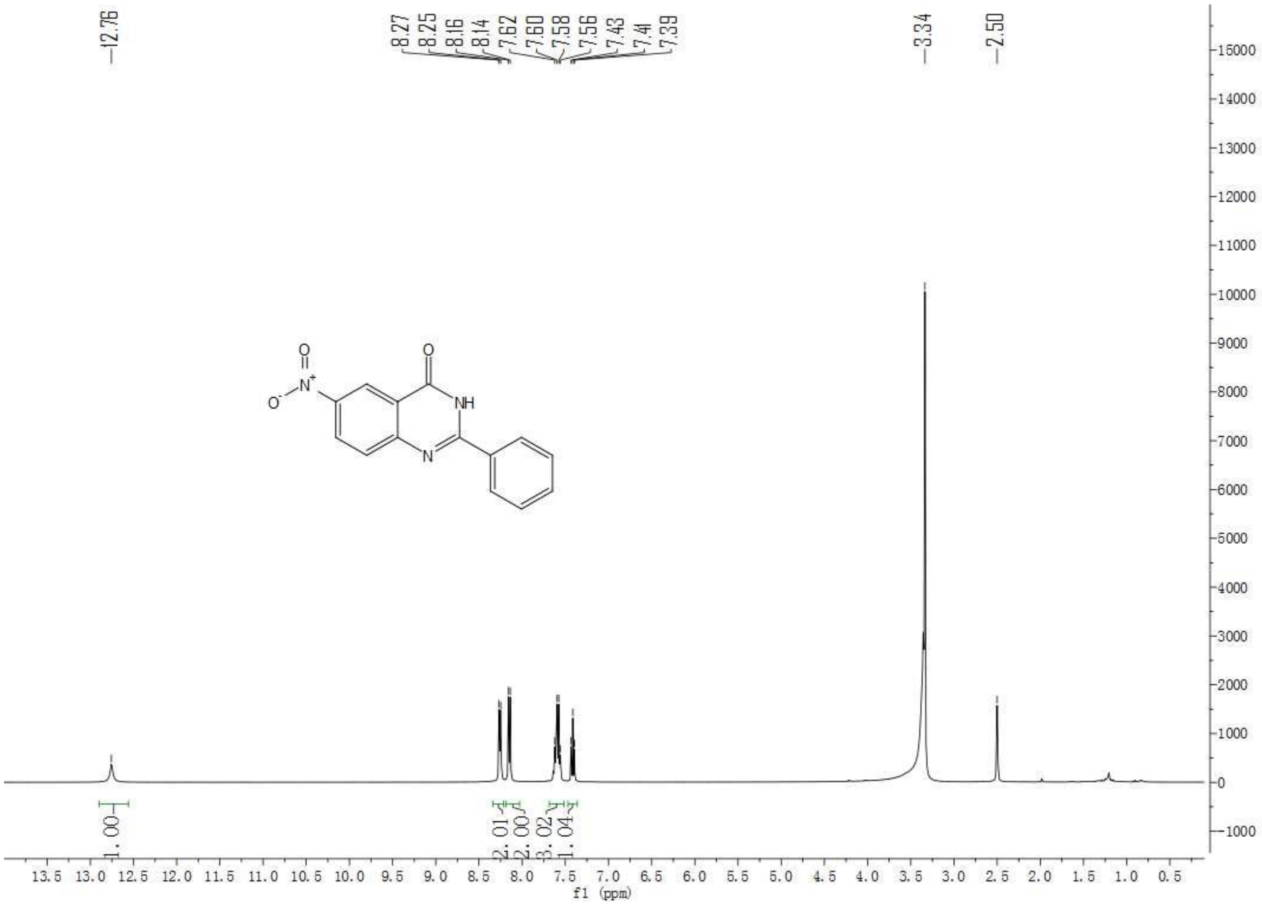

^{1}H NMR spectra (400 MHz, DMSO-d_6) **of 6h**

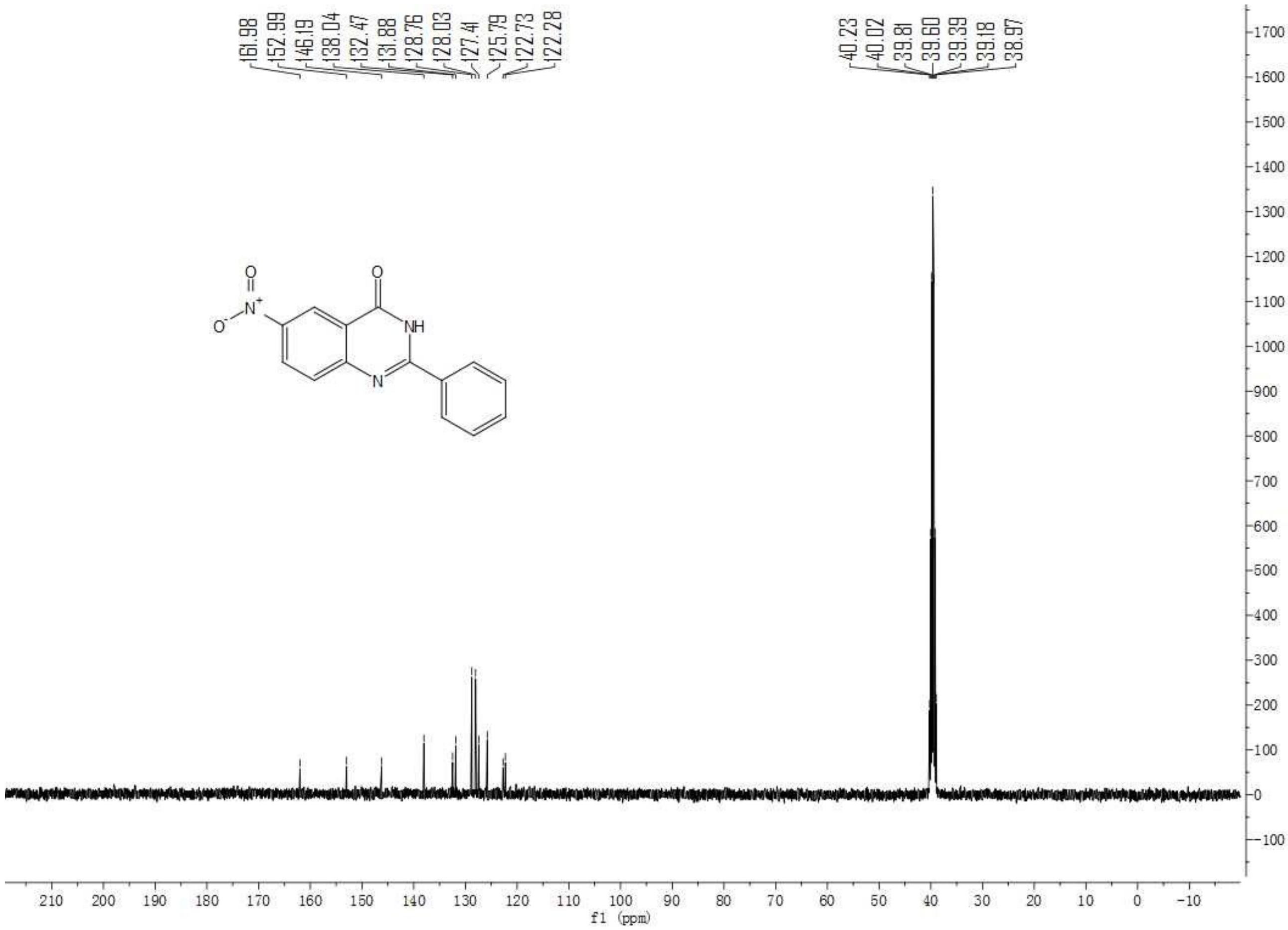

^{13}C NMR spectra (100 MHz, DMSO-d_6) **of 6h**

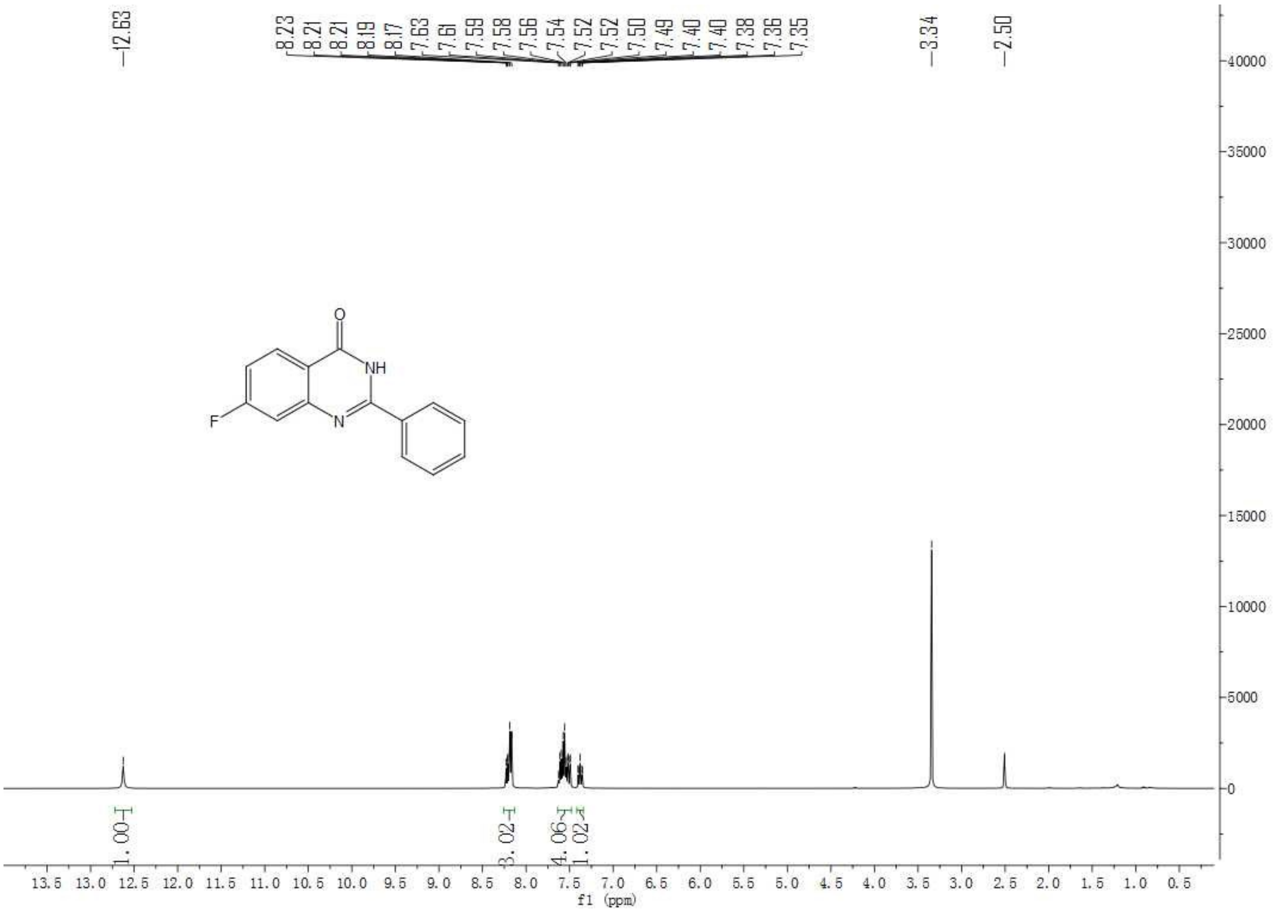

^{1}H NMR spectra (400 MHz, DMSO-d_6) **of 6i**

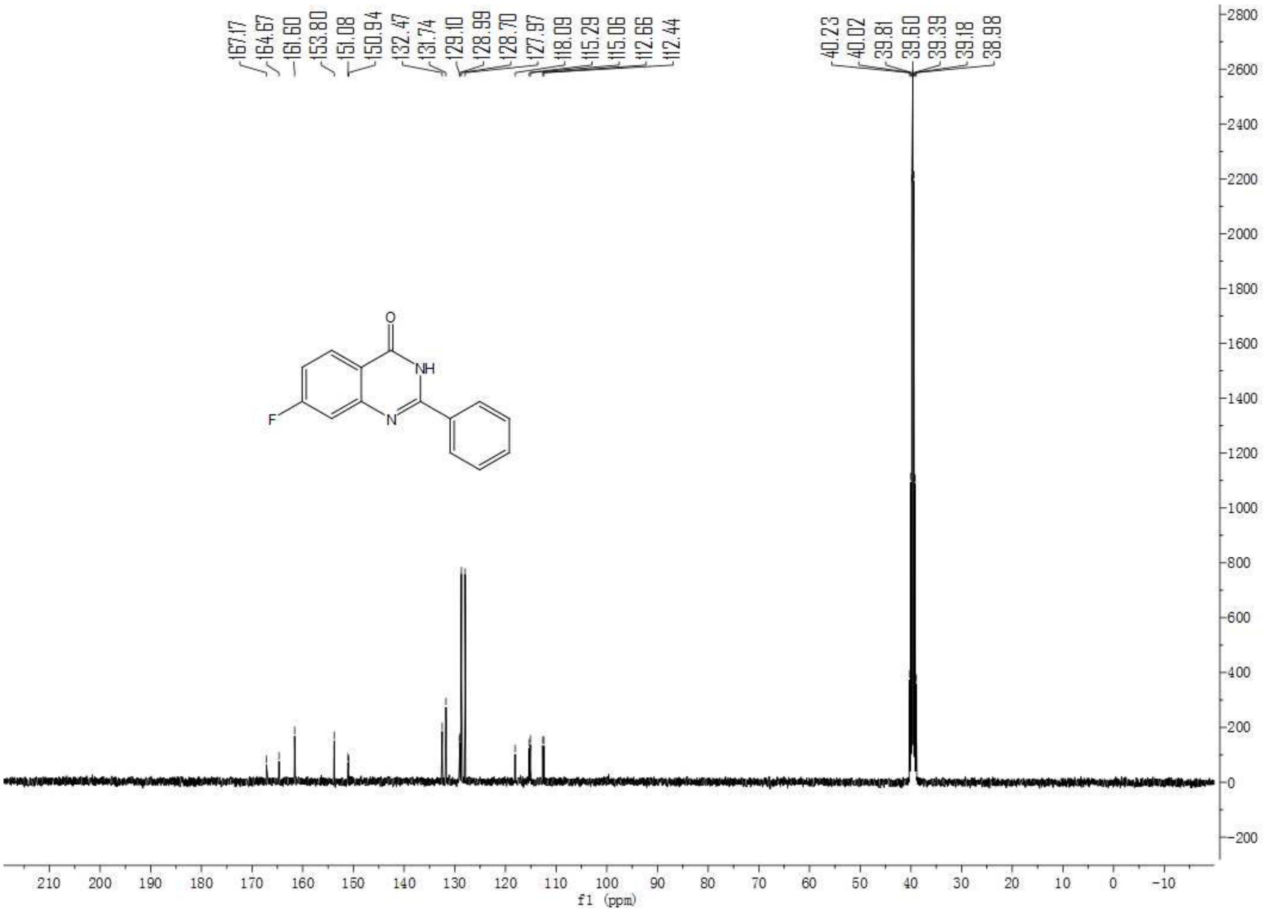

^{13}C NMR spectra (100 MHz, DMSO-d_6) **of 6i**

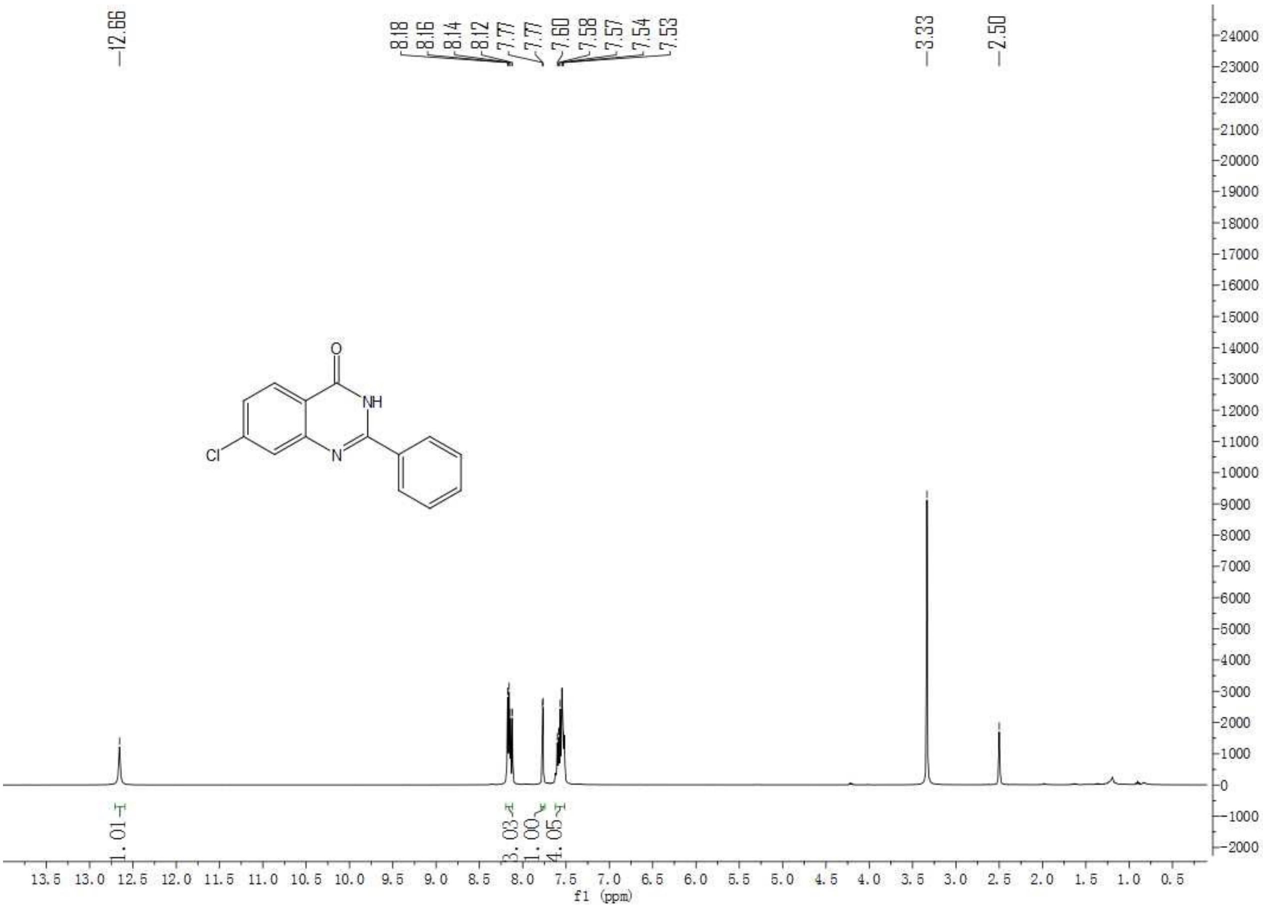

^{1}H NMR spectra (400 MHz, DMSO-d_6) **of 6j**

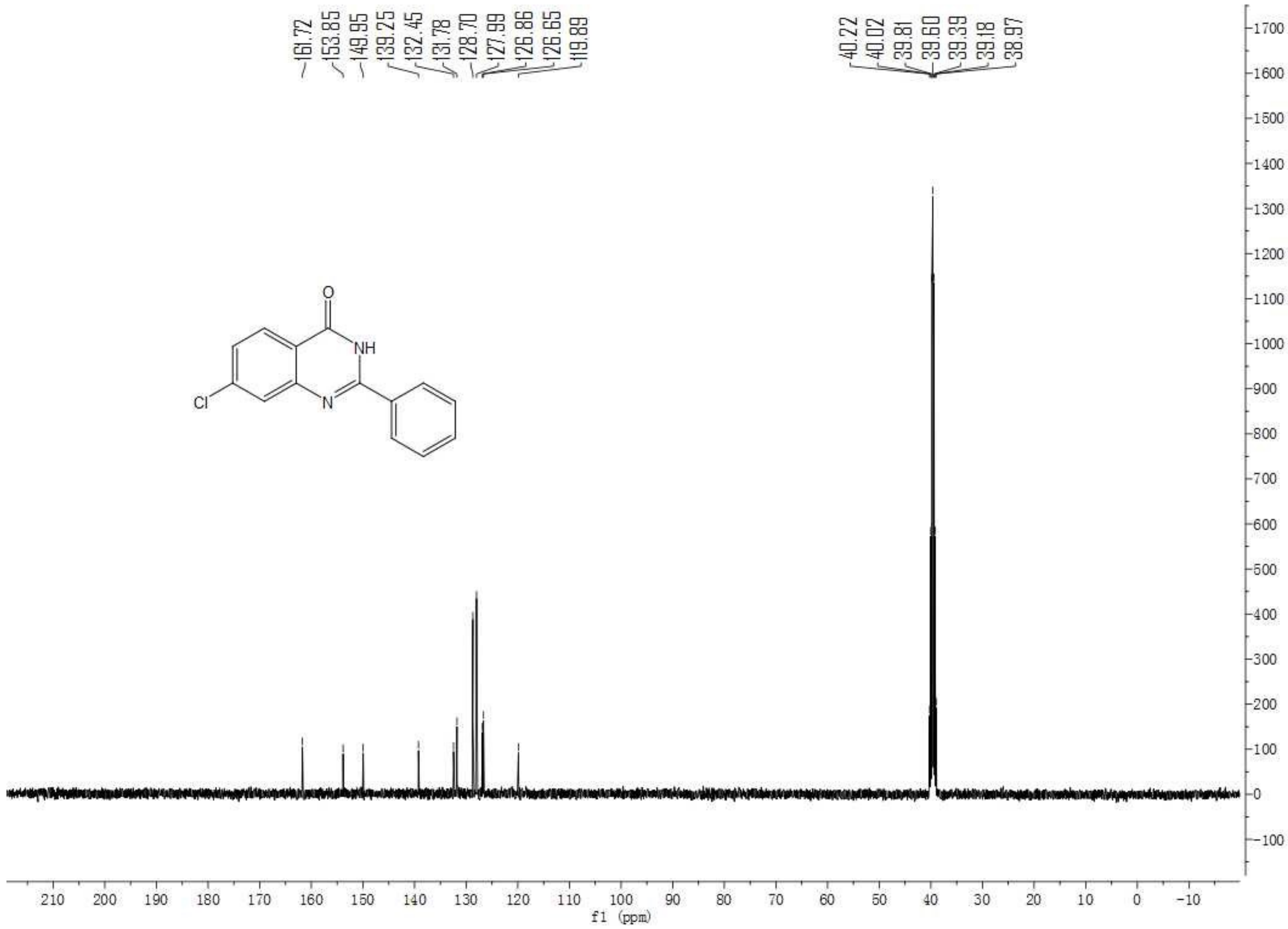

^{13}C NMR spectra (100 MHz, DMSO-d_6) **of 6j**

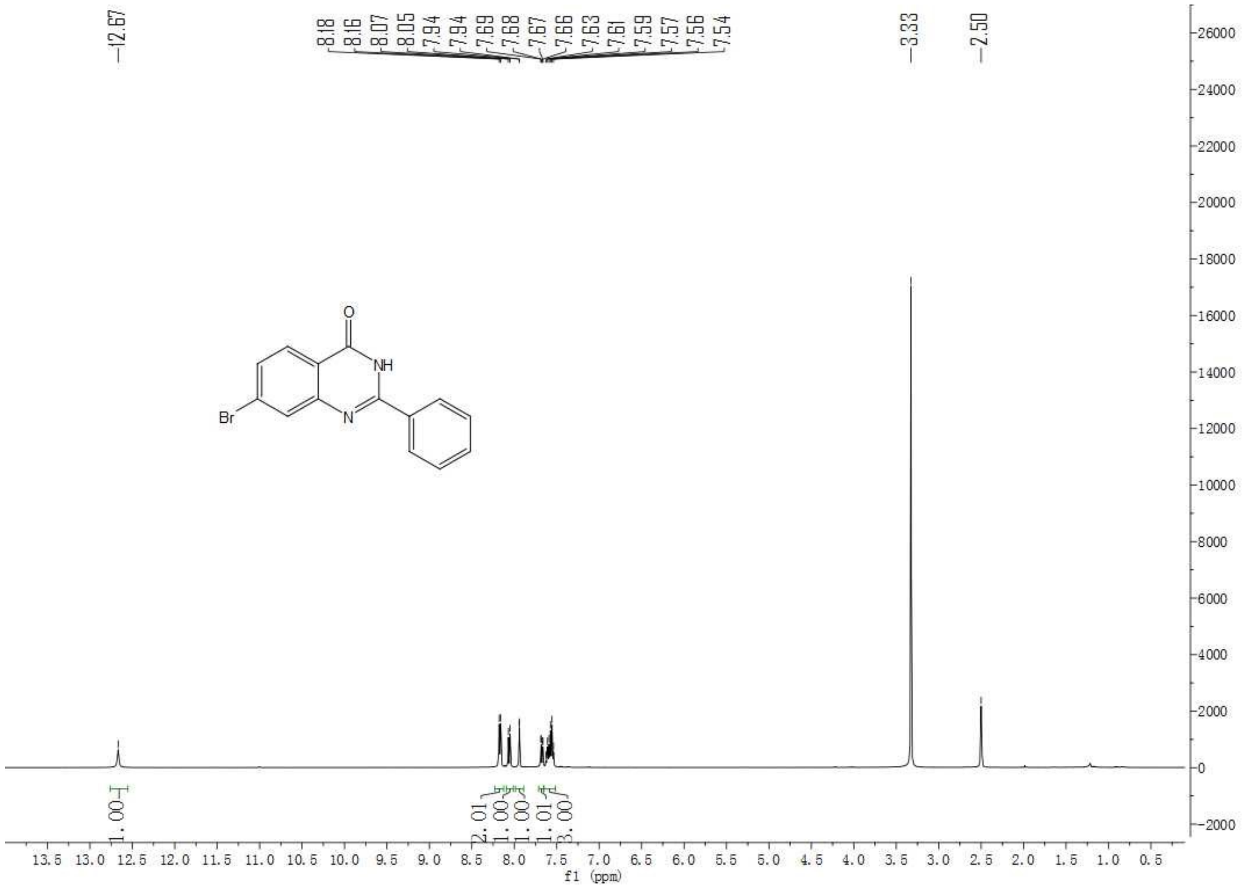

^{1}H NMR spectra (400 MHz, DMSO-d_6**) of 6k**

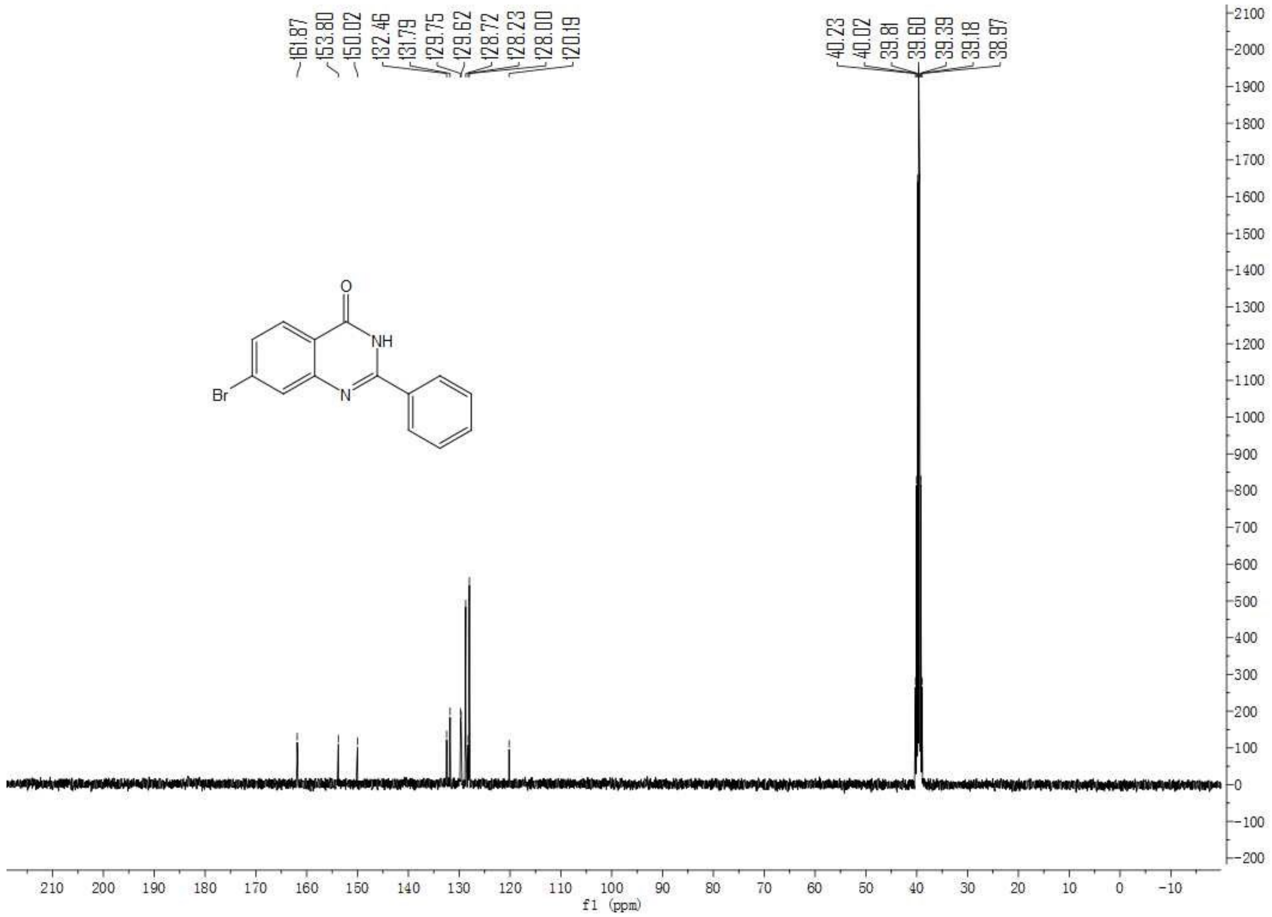

^{13}C NMR spectra (100 MHz, DMSO-d_6**) of 6k**

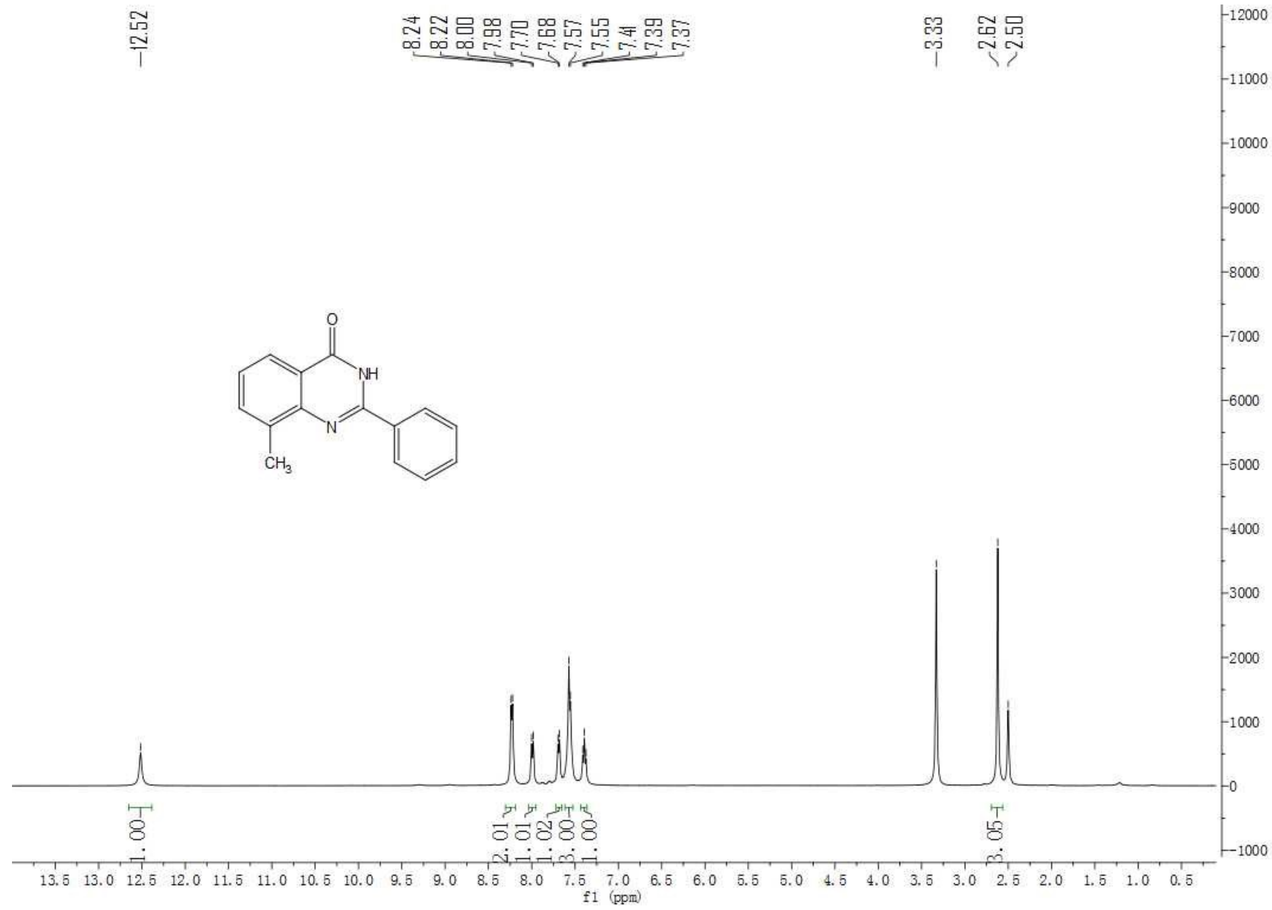

^{1}H NMR spectra (400 MHz, DMSO-d_6) **of 6l**

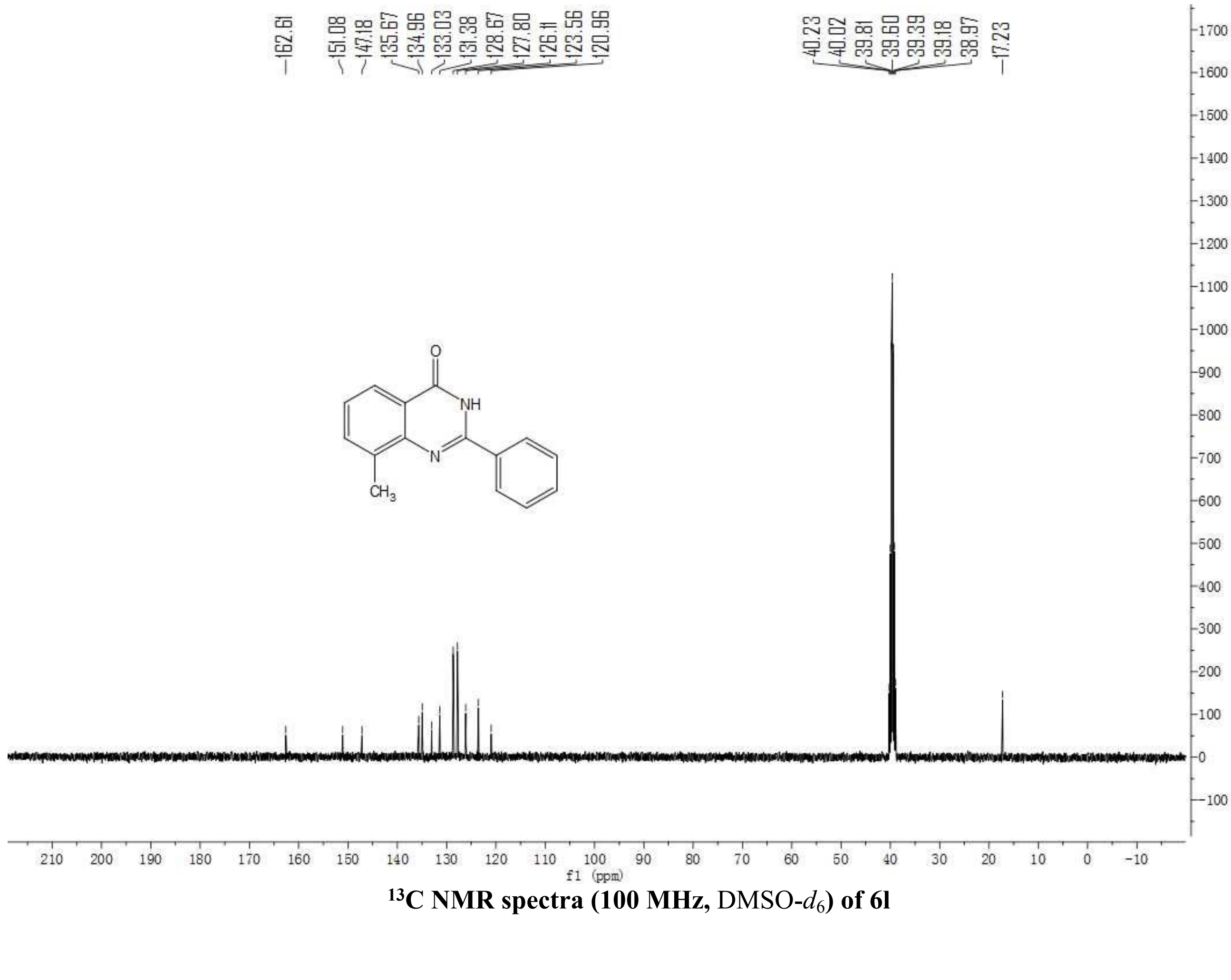

^{13}C NMR spectra (100 MHz, DMSO-d_6) **of 6l**

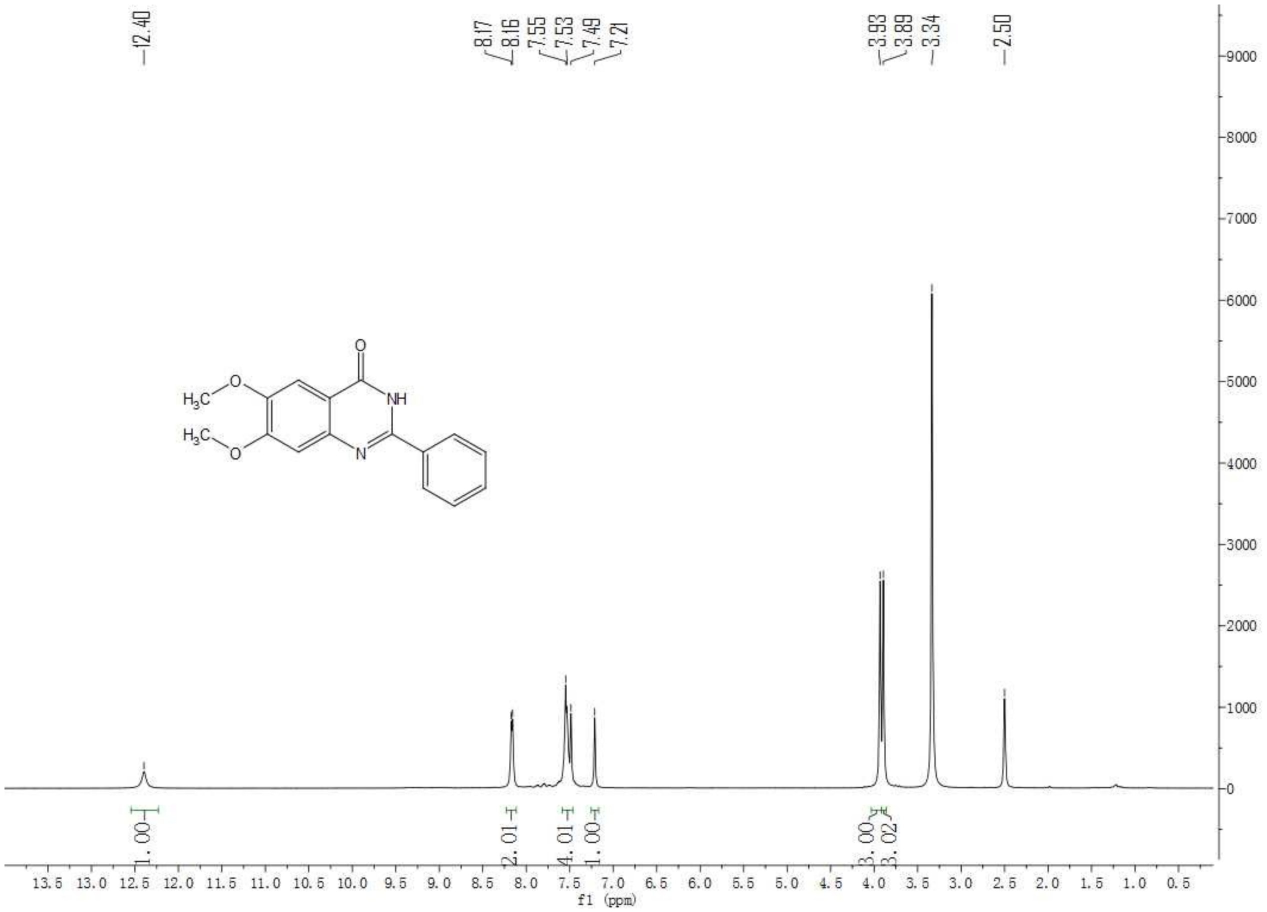

^{1}H NMR spectra (400 MHz, DMSO-d_6**) of 6m**

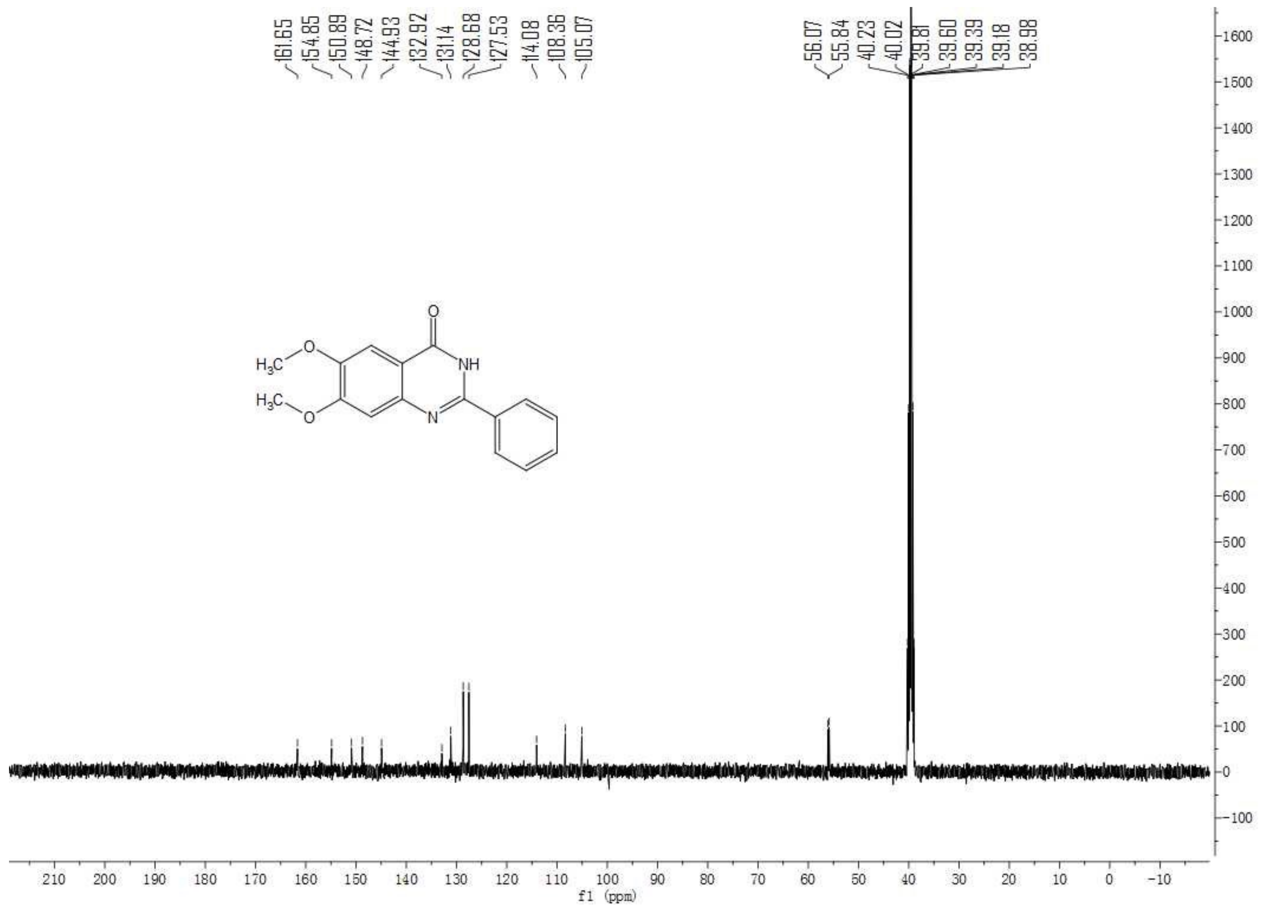

^{13}C NMR spectra (100 MHz, DMSO-d_6**) of 6m**

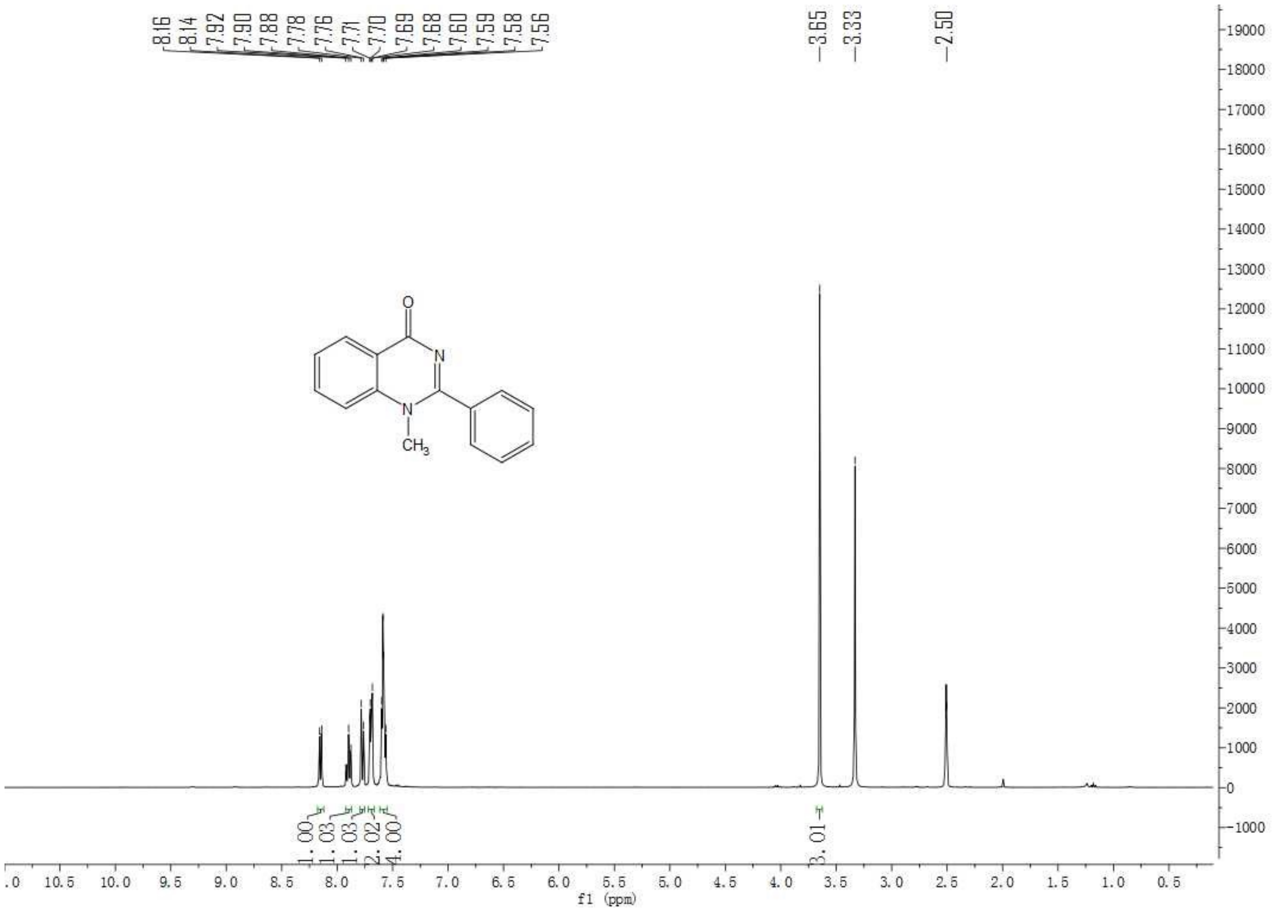

^{1}H NMR spectra (400 MHz, DMSO-d_6**) of 6n**

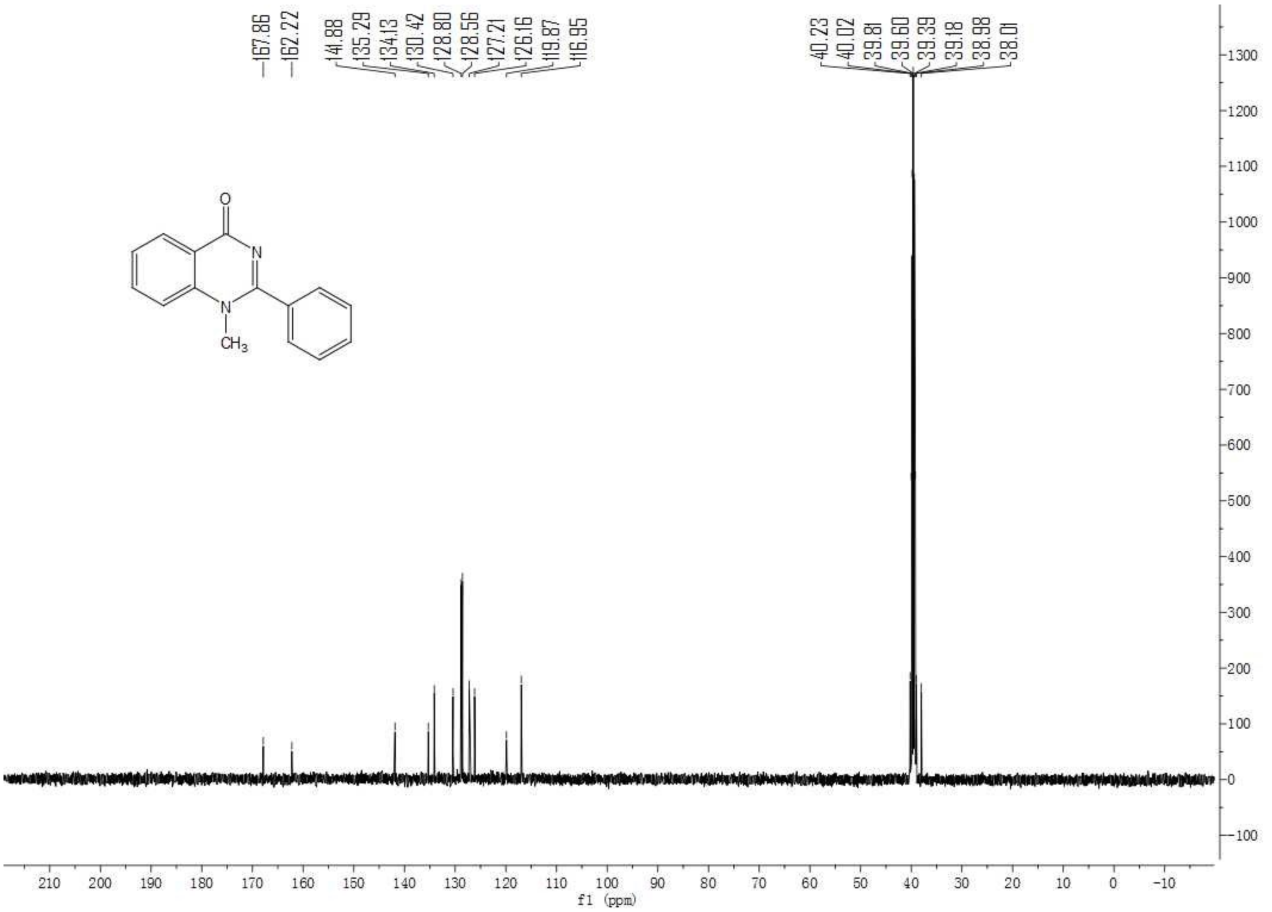

^{13}C NMR spectra (100 MHz, DMSO-d_6**) of 6n**

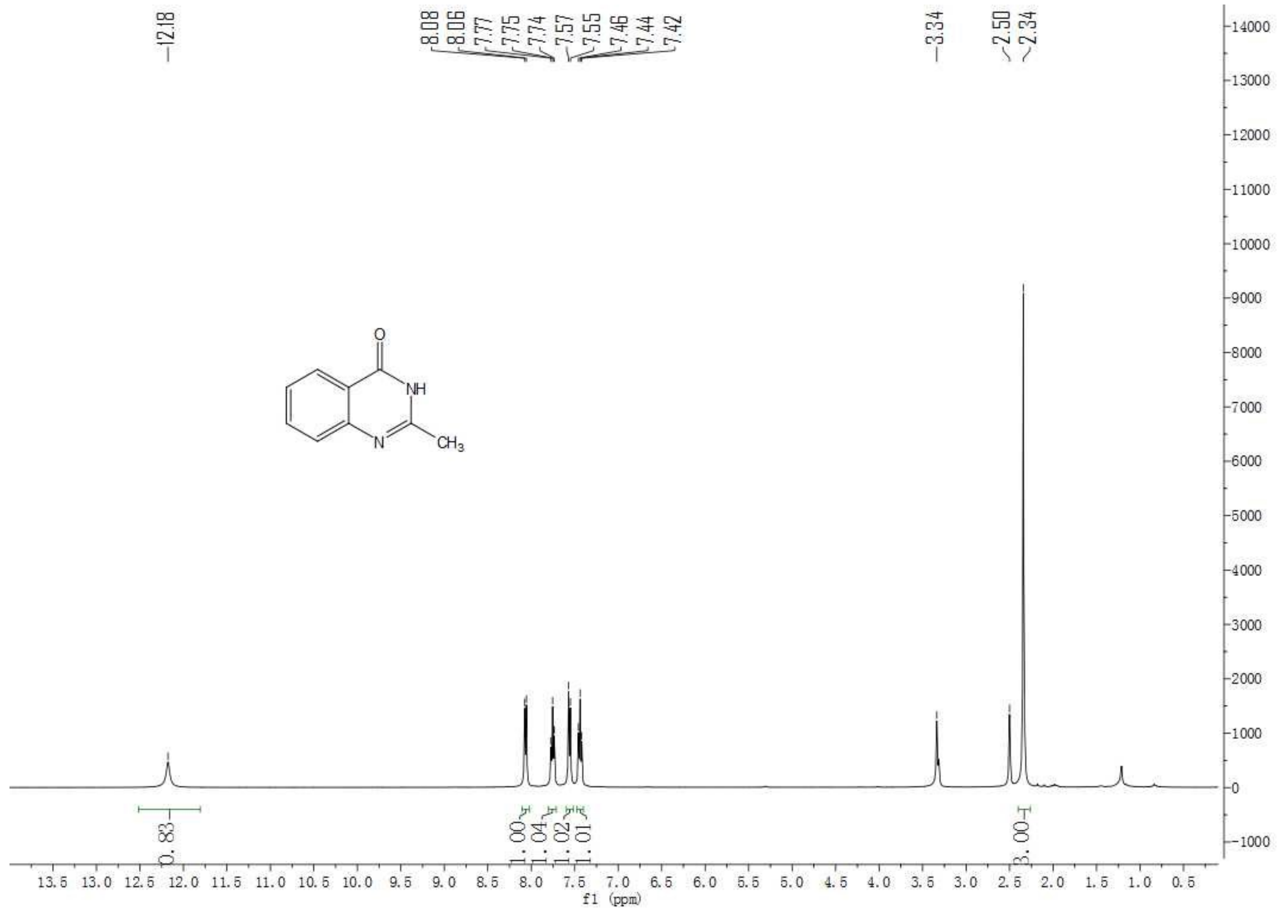

^{1}H NMR spectra (400 MHz, DMSO-d_6**) of 6o**

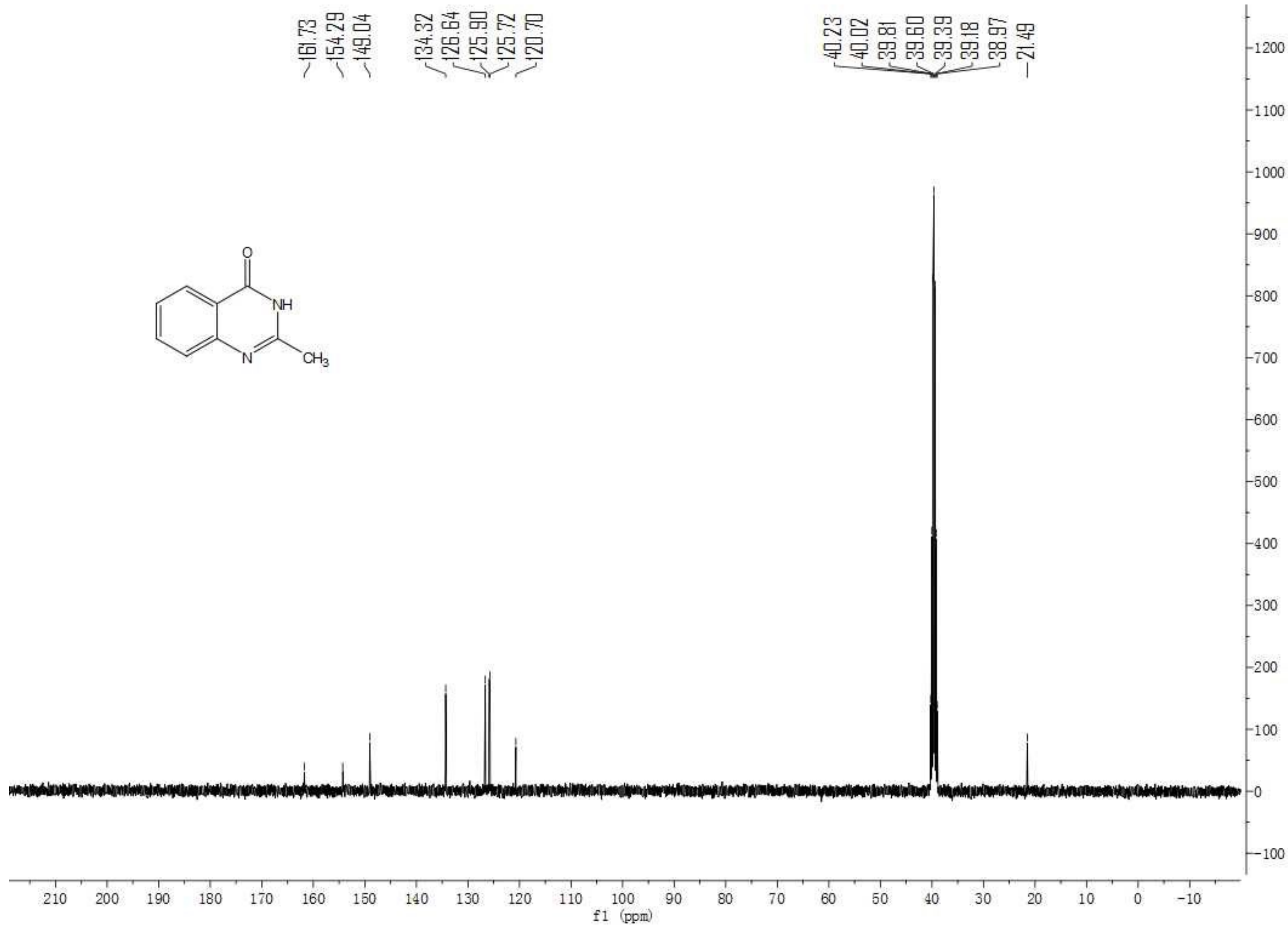

^{13}C NMR spectra (100 MHz, DMSO-d_6**) of 6o**

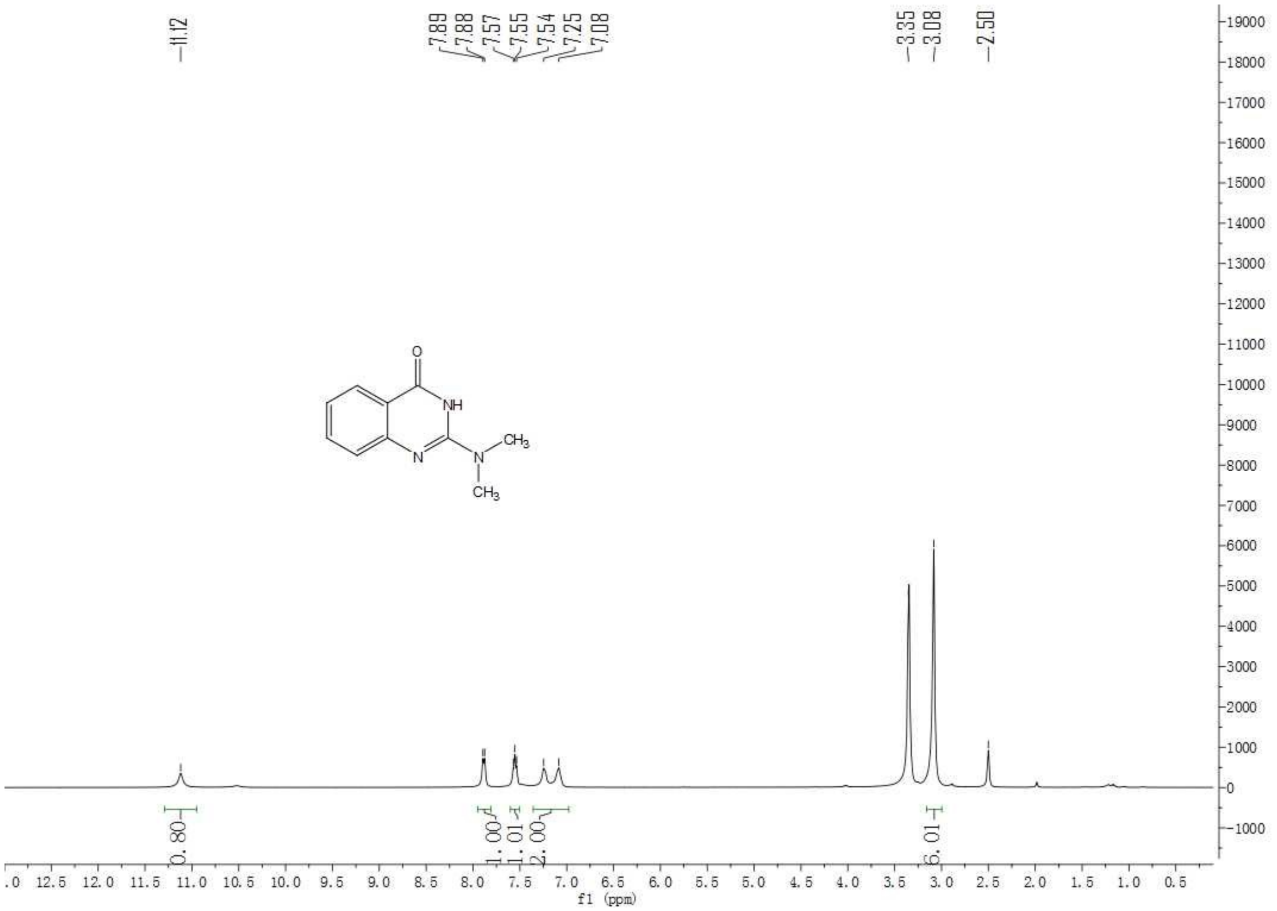

^{1}H NMR spectra (400 MHz, DMSO-d_6**) of 6p**

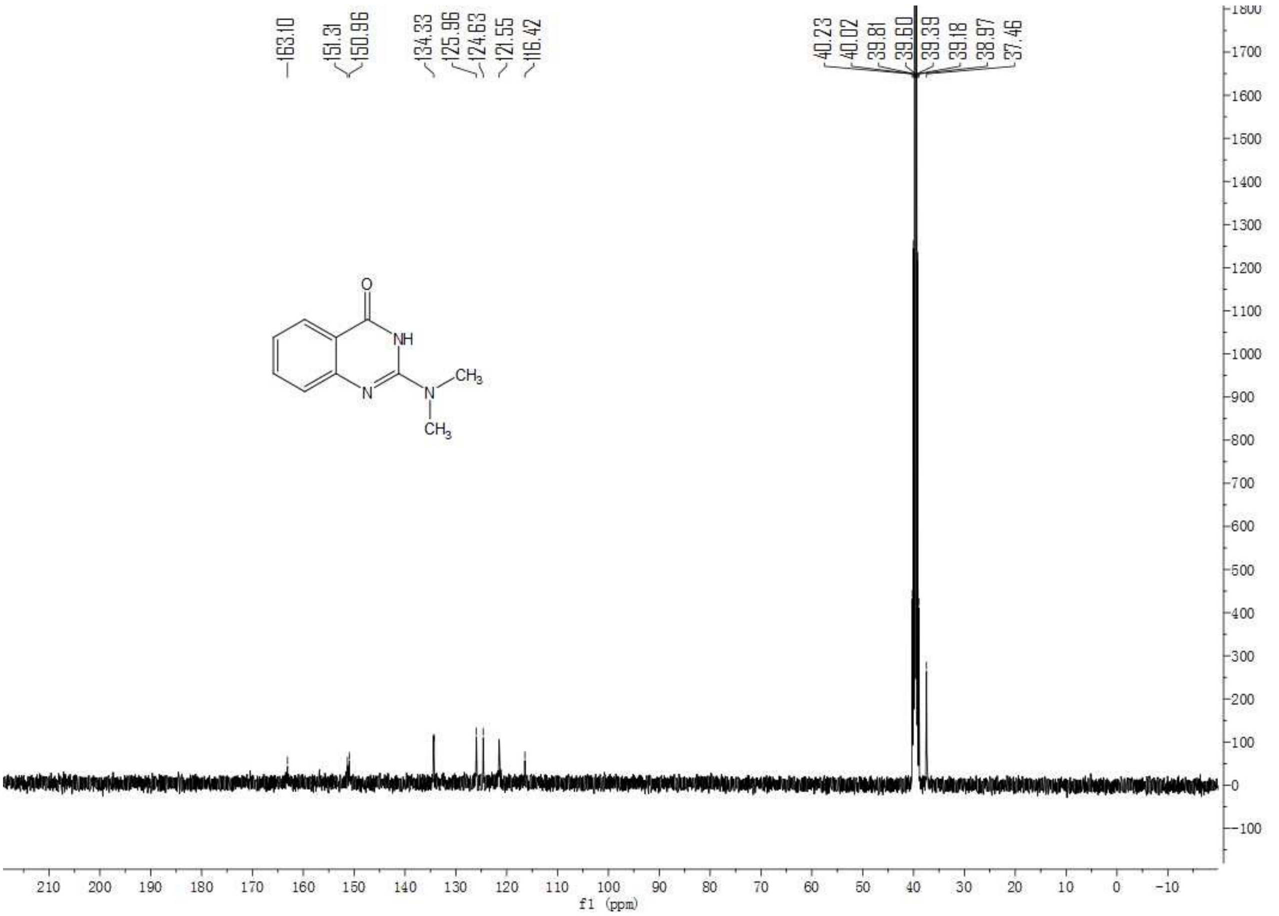

^{13}C NMR spectra (100 MHz, DMSO-d_6**) of 6p**

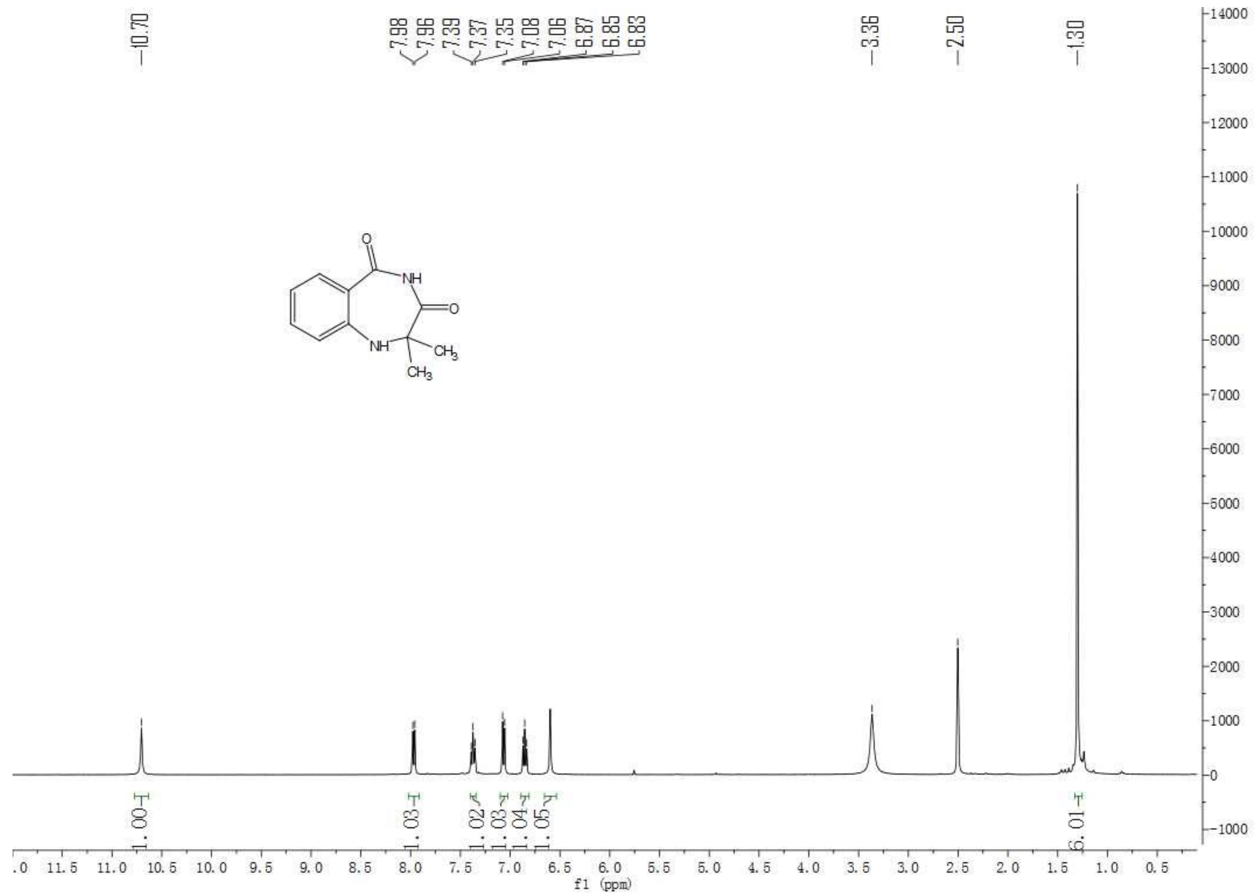

^{1}H NMR spectra (400 MHz, DMSO-d_6) **of 9**

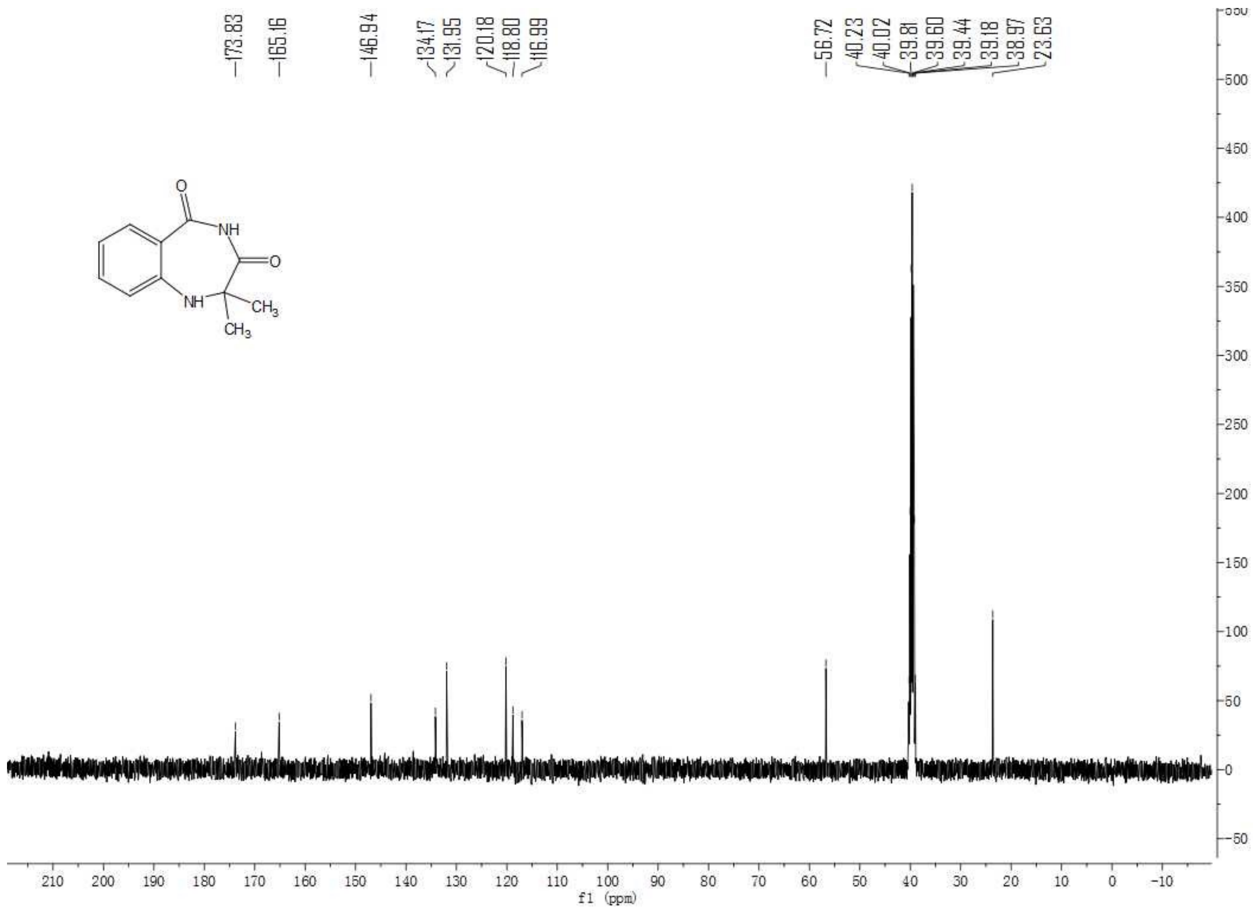

^{13}C NMR spectra (100 MHz, DMSO-d_6) **of 9**

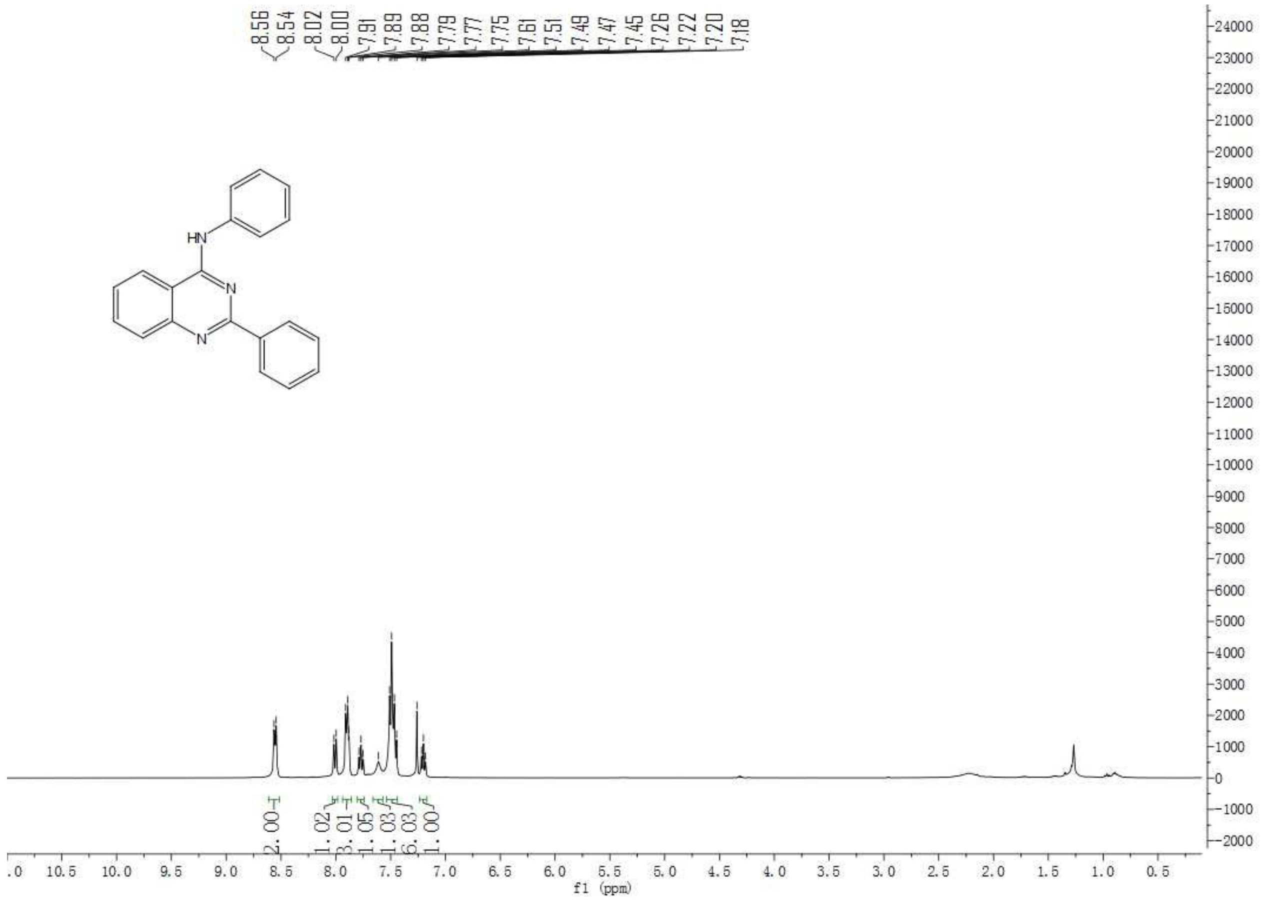

^{1}H NMR spectra (400 MHz, $CDCl_3$) of 10a

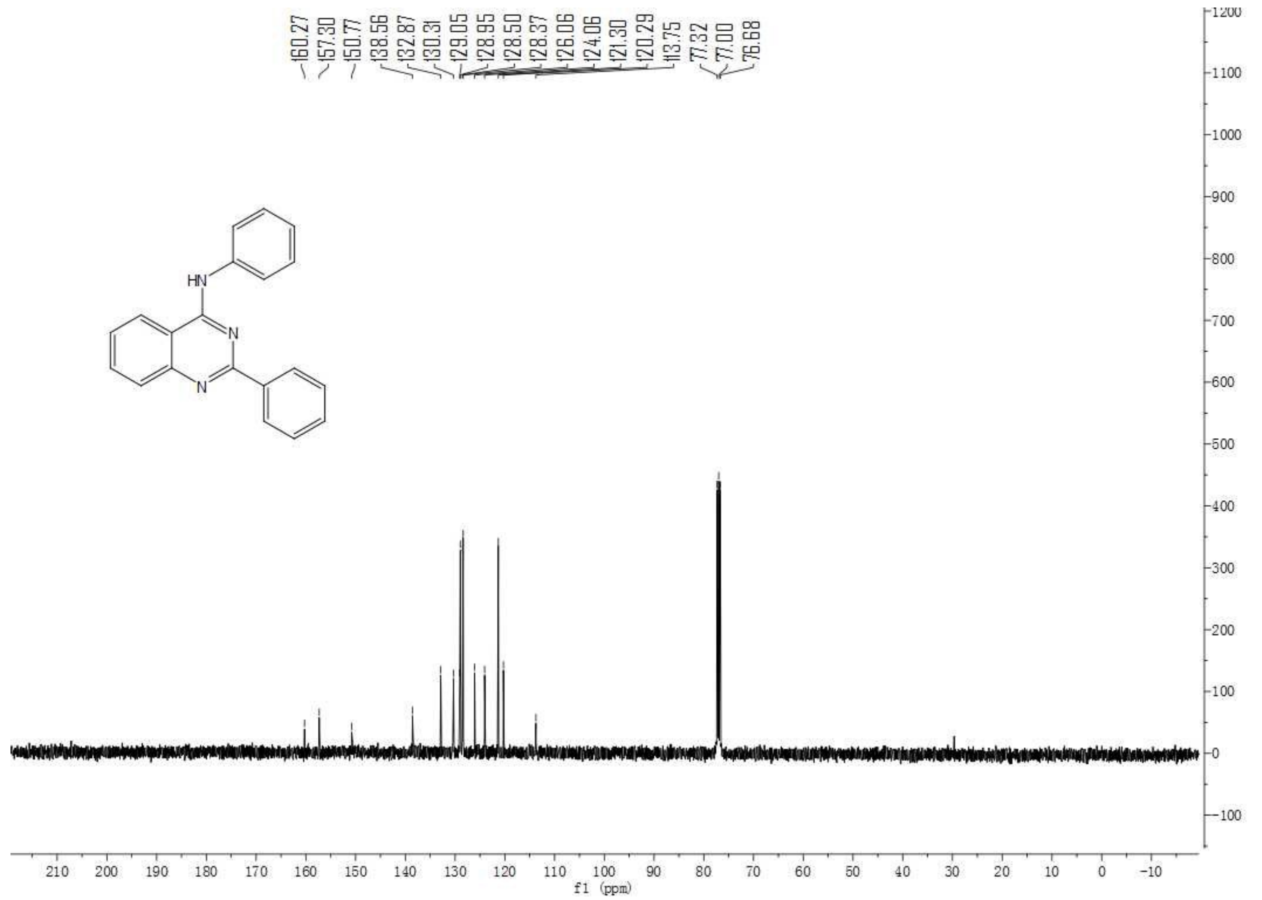

^{13}C NMR spectra (100 MHz, $CDCl_3$) of 10a

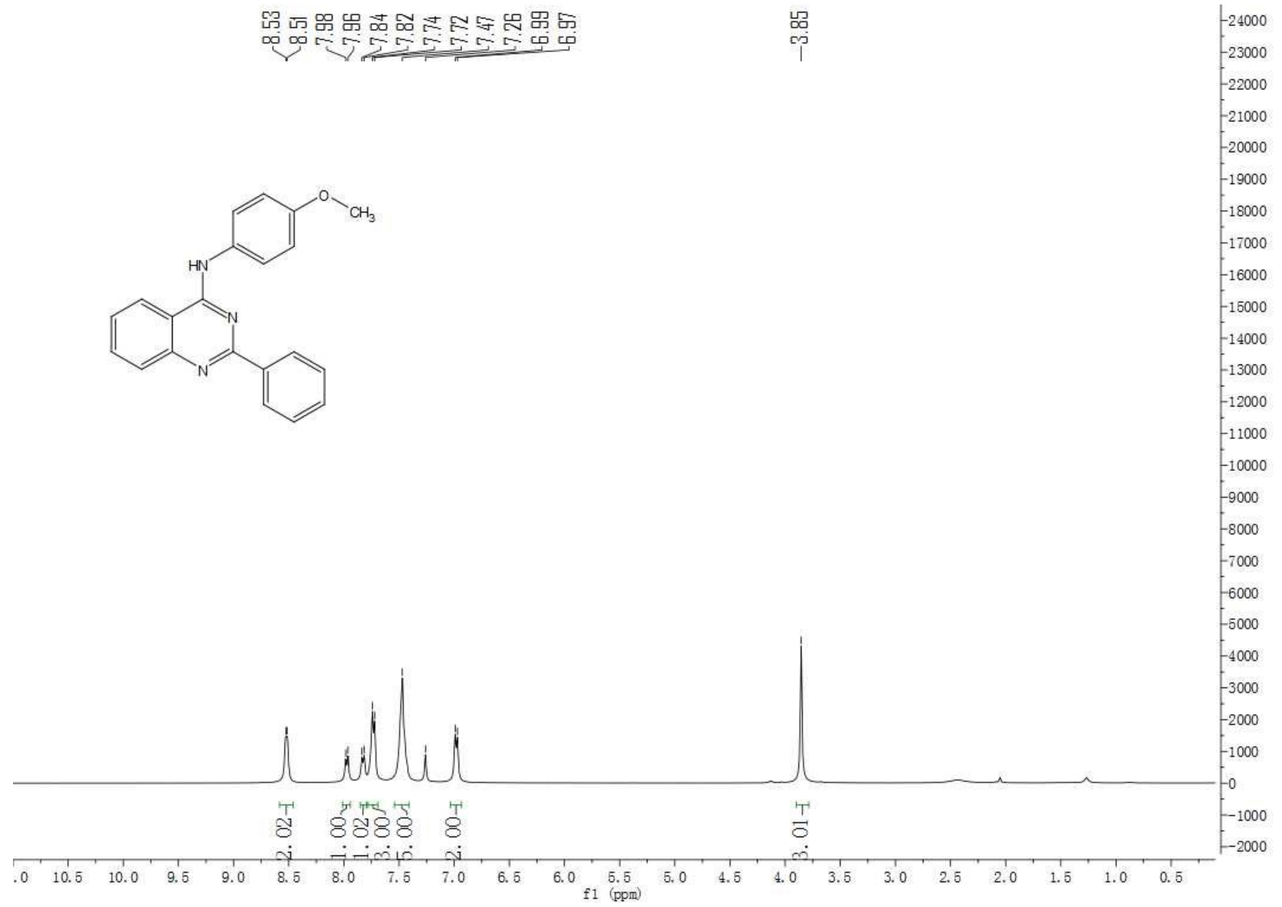

^{1}H NMR spectra (400 MHz, $CDCl_3$) of 10b

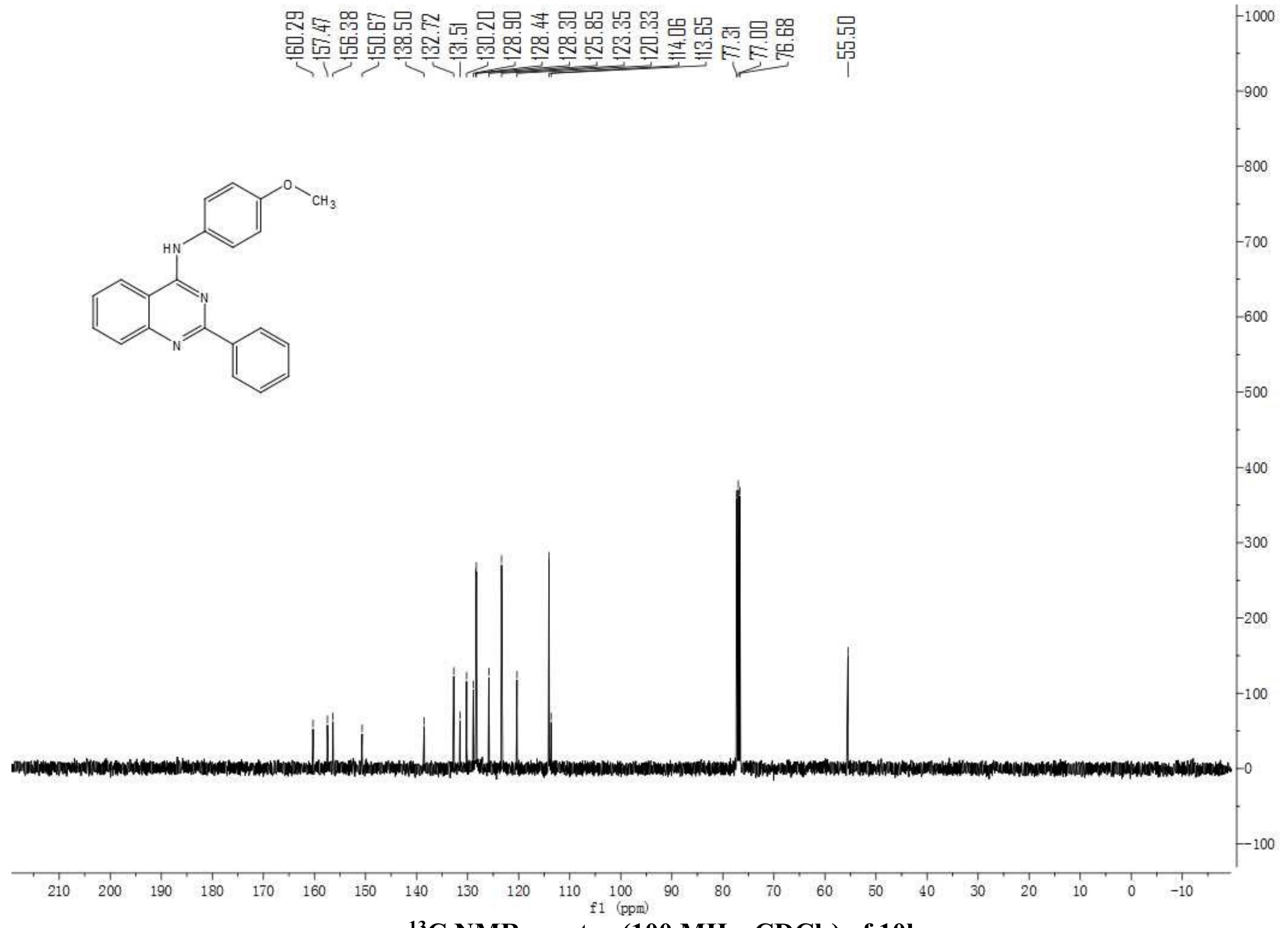

^{13}C NMR spectra (100 MHz, $CDCl_3$) of 10b

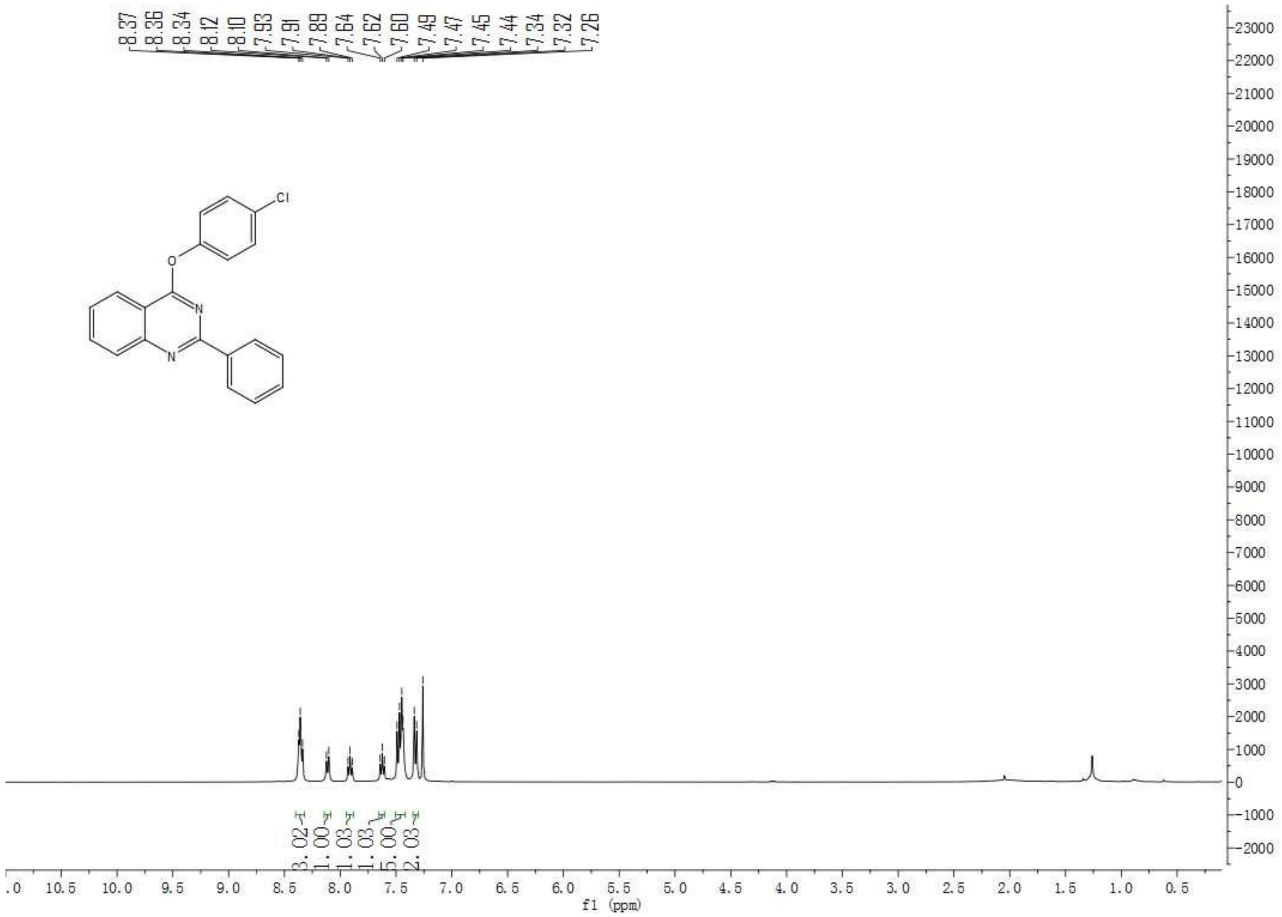

^{1}H NMR spectra (400 MHz, $CDCl_3$) of 11a

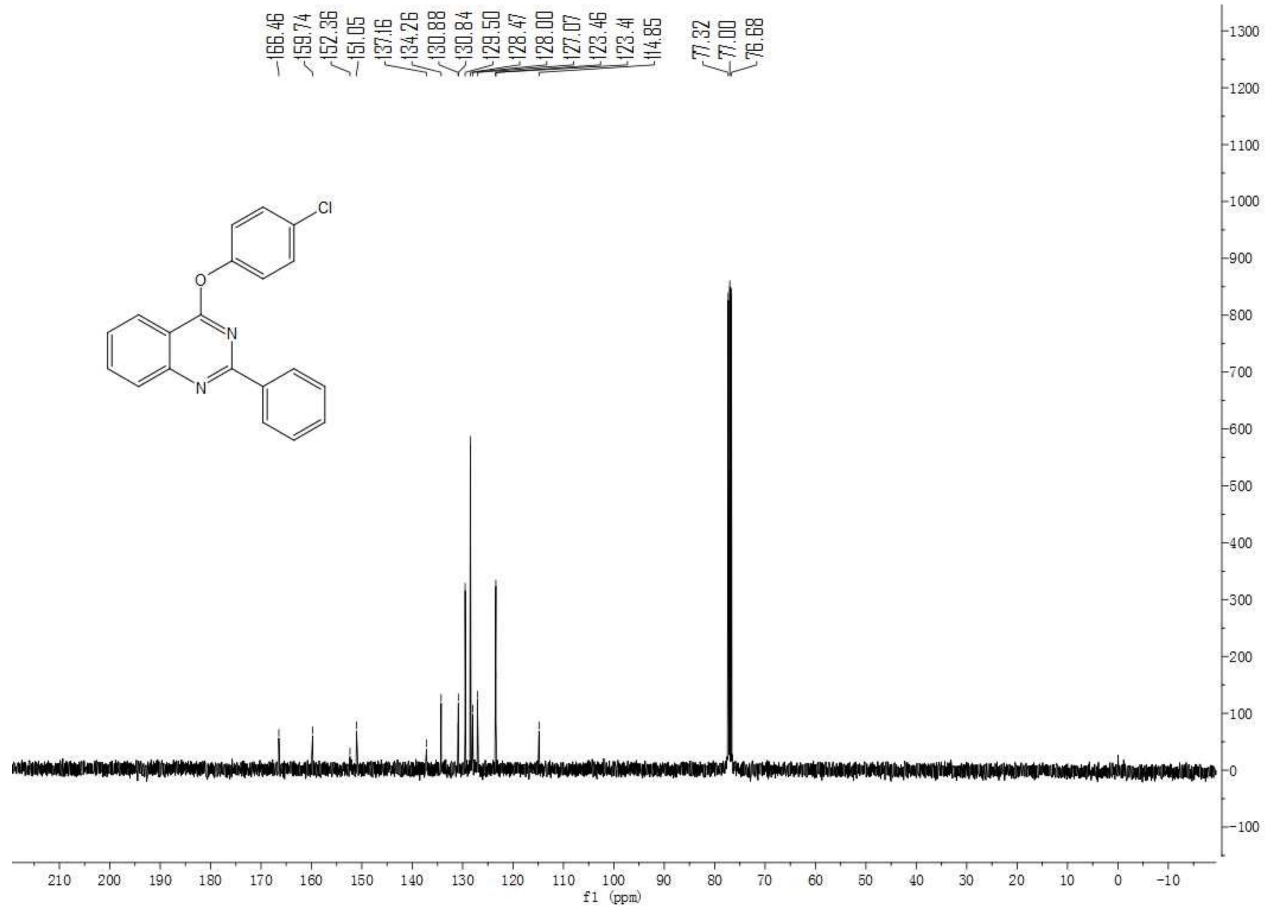

^{13}C NMR spectra (100 MHz, $CDCl_3$) of 11a

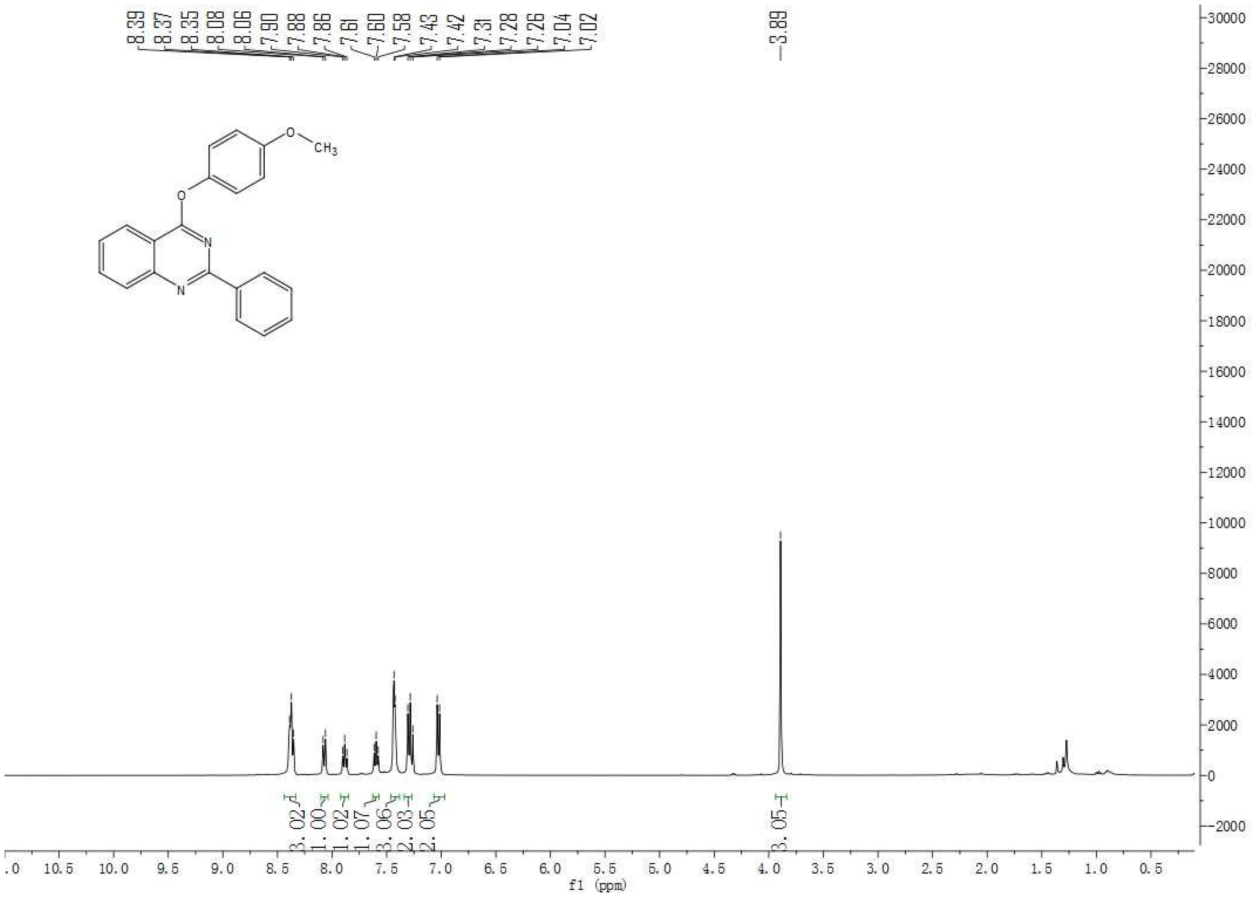

^{1}H NMR spectra (400 MHz, $CDCl_3$) of 11b

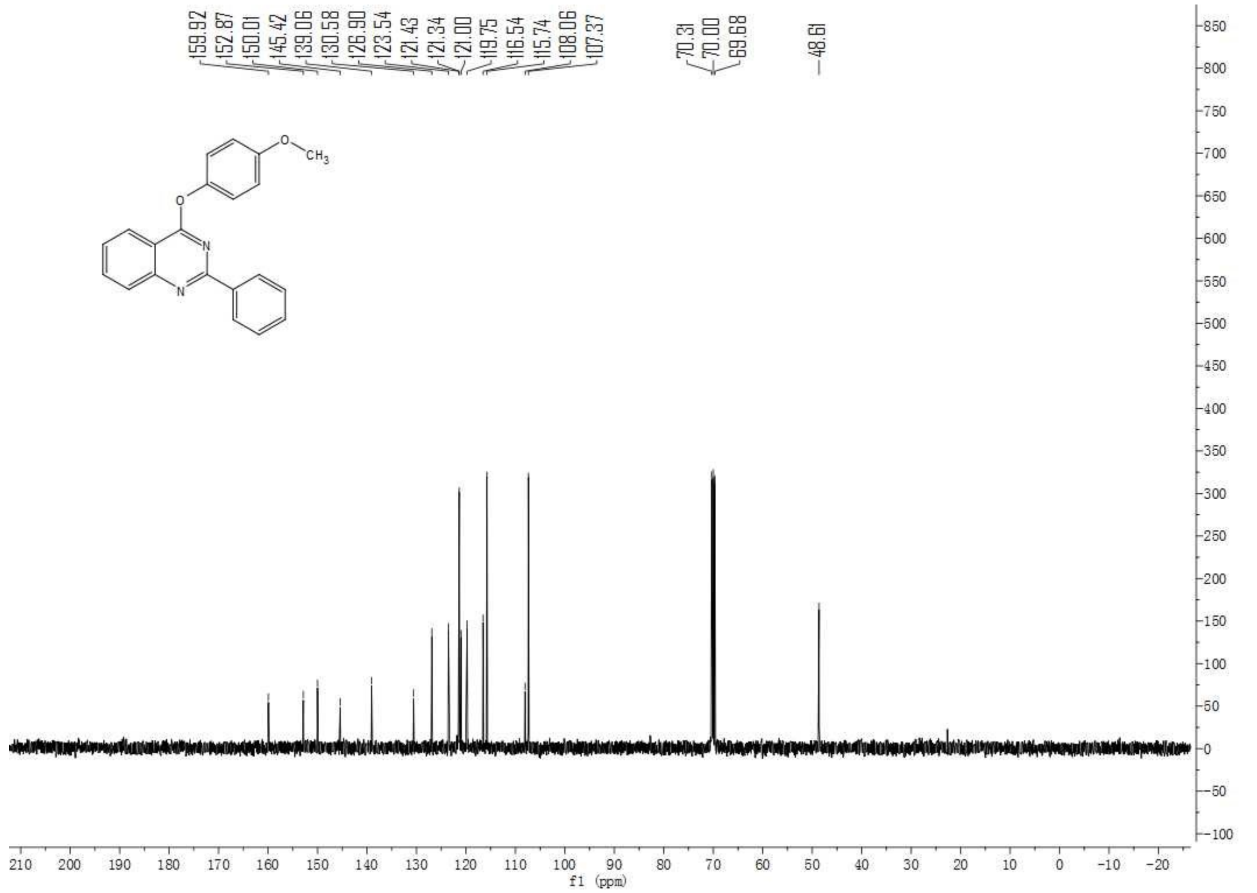

^{13}C NMR spectra (100 MHz, $CDCl_3$) of 11b
